# Electrochemical Synthesis of New Isoxazoles and Triazoles Tethered with Thiouracil Base as Inhibitors of Histone Deacetylases in Human Breast Cancer Cells

**DOI:** 10.3390/molecules28135254

**Published:** 2023-07-06

**Authors:** Divakar Vishwanath, Zhang Xi, Akshay Ravish, Arunkumar Mohan, Shreeja Basappa, Niranjan Pattehalli Krishnamurthy, Santosh L. Gaonkar, Vijay Pandey, Peter E. Lobie, Basappa Basappa

**Affiliations:** 1 Laboratory of Chemical Biology, Department of Studies in Organic Chemistry, University of Mysore, Mysore 570006, Karnataka, India; divakardivi166@gmail.com (D.V.); akshayrv533@gmail.com (A.R.); arunmysore3@gmail.com (A.M.); 2 Shenzhen Bay Laboratory, Shenzhen 518055, China; zhangxi@szbl.ac.cn; 3 Department of Chemistry, BITS–Pilani Hyderabad Campus, Jawaharnagar 500078, Medchal, Telangana, India; f20210833@hyderabad.bits-pilani.ac.in; 4 NMR Research Centre, Indian Institute of Science, Bangalore 560012, Karnataka, India; niranjanursa@gmail.com; 5 Department of Chemistry, Manipal Institute of Technology, Manipal Academy of Higher Education, Manipal 576104, Karnataka, India; sl.gaonkar@manipal.edu; 6 Tsinghua Berkeley Shenzhen Institute, Tsinghua Shenzhen International Graduate School, Tsinghua University, Shenzhen 518055, China; vijay.pandey@sz.tsinghua.edu.cn; 7 Institute of Biopharmaceutical and Health Engineering, Tsinghua Shenzhen International Graduate School, Tsinghua University, Shenzhen 518055, China

**Keywords:** breast cancer, HDAC, triazole, isoxazole, thiouracil, drug discovery, cell viability assay, molecular docking

## Abstract

Histone deacetylases (HDACs) are an attractive drug target for the treatment of human breast cancer (BC), and therefore, HDAC inhibitors (HDACis) are being used in preclinical and clinical studies. The need to understand the scope of the mode of action of HDACis, as well as the report of the co-crystal structure of HDAC6/SS-208 at the catalytic site, provoked us to develop an isoxazole-based lead structure called 4-(2-(((1-(3,4-dichlorophenyl)-1H-1,2,3-triazol-4-yl)methyl)thio) pyrimidin-4-yl) morpholine (**5h**) and 1-(2-(((3-(p-tolyl) isoxazol-5-yl)methyl)thio) pyrimidin-4-yl) piperidin-4-one (**6l**) that targets HDACs in human BC cells. We found that the compound **5h** or **6l** could inhibit the proliferation of BC cells with an IC_50_ value of 8.754 and 11.71 µM, respectively. Our detailed *in silico* analysis showed that **5h or 6l** compounds could target HDAC in MCF-7 cells. In conclusion, we identified a new structure bearing triazole, isoxazole, and thiouracil moiety, which could target HDAC in MCF-7 cells and serve as a base to make new drugs against cancer.

## 1. Introduction

Histone deacetylases (HDACs) remove acetyl groups from lysine residues on acetylated histones, leading to changes in chromatin structure and gene expression. In breast cancer (BC), HDACs were found to be overexpressed and regulate the expression of a tumor-suppressor gene called BRCA1, which was involved in DNA repair, cell-cycle control, and cancer-cell progression [1,2,3,4]. In addition, HDACs play an essential role in oncogenic pathways, such as the estrogen-receptor (ER) signaling pathway, which leads to ER-positive BC [5]. Structurally, the catalytic site of HDACs contains four regions of highly conserved amino acids, where the first region is referred to as a zinc-binding domain, which is coordinated by two histidines and one aspartic acid residue [6,7,8]. The second region was known as the catalytic triad (His-Asp-Tyr) domain, which consists of three amino acid residues involved in the deacetylation reaction [9]. Moreover, the active site of HDACs contains a hydrophobic pocket region with phenylalanine and tyrosine-rich amino acids that bind to the histone substrate’s acetyl group [10]. Finally, the catalytical site of HDACs has a lysine binding domain composed of asparagine, glutamic acid, and leucine amino acids, which were found to interact with the ε-amino group of the lysine residue on the histone substrate [11].

In recent years, HDACs have emerged as promising drug targets for treating cancer and other neurological diseases [12]. HDAC inhibitors mainly modulate gene expressions and induce apoptosis and cell differentiation; therefore, HDACs are promising drug targets. The HDAC inhibitors (HDACis) were broadly subclassified into four classes called hydroxamic acids, short-chain fatty acids, cyclic peptides, and benzamides, and the US FDA approved some of them for the treatment of patients. Vorinostat, Belinostat, Panobinostat, Givinostat, Trichostatin A, Scriptaid, JNJ-26481585, MC1568, MC-1293, and ACY-1215 are the drugs bearing hydroxamic acid in their chemical structure [13,14,15,16,17,18,19,20,21]. HDACis, like valproic acid and sodium butyrate, fall under the short-chain fatty acids class, while Romidepsin, Apicidin, and Lagazole Thiol fall under cyclic peptides [13,22]. Mocetinostat, CI-994, RGFP966, Tubastatin A, and TMP195 are a few HDACis from the benzamide category [13,23,24,25].

In this regard, we previously demonstrated that the lead compound 3-(4-(4-phenoxy phenyl)-1*H*-1,2,3-triazol-1-yl)benzo[*d*]isoxazole (**1**) targeted histone deacetylases in cancer cells, which displayed a high degree of shape complementarily to the binding site of the enzyme [26]. Triazole-based carboxamides, such as the SAHA analog (**2**), exhibited potent inhibitory activity towards HDAC8 and were shown to be a potential anticancer agent and induce antitumor effects in preclinical studies [27].

The potent molecule with a thiouracil core, like (*E*)-*N*-hydroxy-3-(4-(((6-oxo-4-phenyl- 1,6-dihydropyrimidin-2-yl)thio)methyl)phenyl)acrylamide (**3**), selectively inhibited HDAC1 (class I) activity in a cell-based assay [28]. Additionally, the studies on thiouracil-based compounds such as compound (**4**) were found to be the most potent and class I-selective HDACi of the tested series, exhibiting anticancer activity [29].

In addition, the co-crystal structure of HDAC6 and an anticancer molecule called isoxazole-3-hydroxamate (**5**) complex revealed that it is bound to the zinc-binding domain and a nearby hydrophobic region [30]. Another isoxazole-based derivative 5-(3-bromo-4-hydroxyphenyl)-3-(thiophene-2-yl) isoxazole (**6**) showed an anticancer effect by acting as an HDAC inhibitor [31].

**Figure 1:**
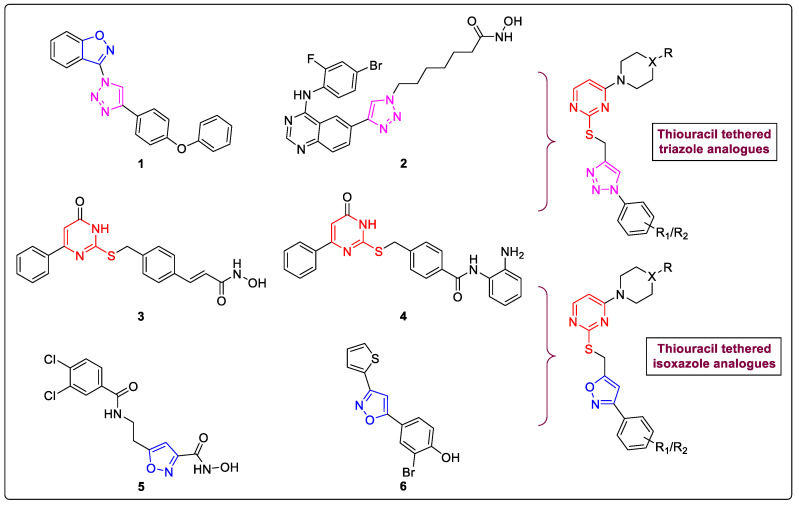
Evolution of HDACis based on a hybrid structure bearing triazole or isoxazole and thiouracil-based core heterocycles; Structures **1** and **2** represents 1,2,3-triazoles (pink), **3** and **4** represents thiouracil (red) analogues also **5** and **6** represents isoxazole (blue) derivatives as HDACis inhibitors.

**Figure 2:**
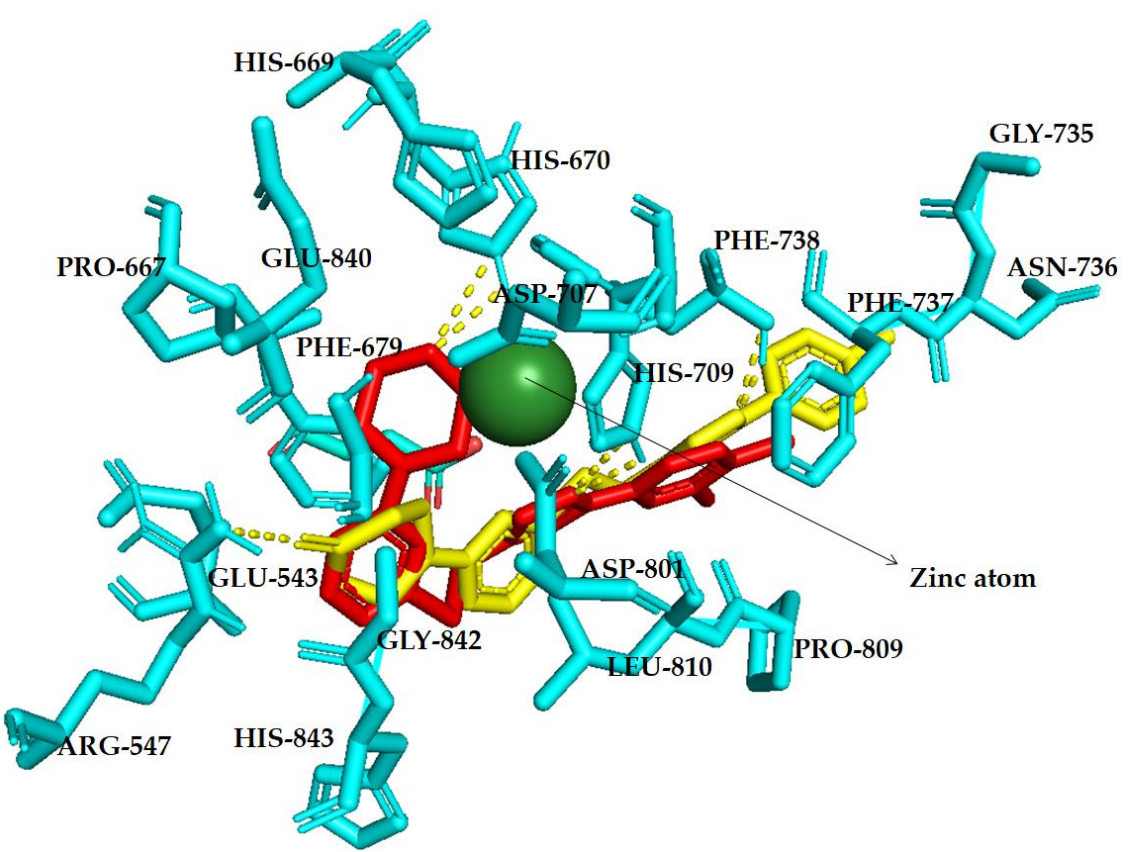
The docked lead triazole (**5h**; red) and lead isoxazole (**6l**; yellow) were visually represented in a detailed stick form, showing their evolution as HDACis on the isoxazole and triazole scaffold. The HDAC (cyan-HDAC7) active site, which contains zinc (Zn^2+^, green), was the target of these compounds. Within the active site is a zinc-binding site and two Asp residues (Asp801–Asp707). The walls of the pocket are lined with hydrophobic residues (Pro667, Phe679, Phe737, Phe738, Pro809 and Leu810), while other residues present include His709, His670, ARG547, Gly842 and others. Doted lines (yellow) represents hydrogen bondings.

Based on our laboratory results on the triazole–isoxazole hybrid structure and the role of substitute thiouracil on HDACis role, we designed a novel structure and developed an extensive library of compounds that could serve as a base to develop new drugs that target HDAC in BC (Figure 1 and Figure 2).

## 2. Results and Discussion

### 2.1. Synthesis of Thiouracil Tethered Triazole/Isoxazole Analogues

**Scheme 1:**
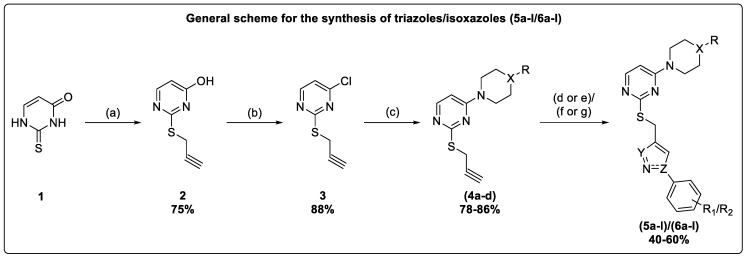
Reagents and conditions: (**a**) propargyl bromide, KOH, EtOH:H_2_O (1:1), reflux at 50 °C, 45 min; (**b**) POCl_3_, reflux, 20 min; (**c**) substituted piperazines/morpholine/4-piperidinone, acetone, Et_3_N, reflux, 8–10 h; (**d**) substituted phenyl azides, CuI, THF, Et_3_N, r.t., 3–5 h; (**e**) Cu foil and Pt electrodes, *tert*-butyl alcohol/water (1:1), tetrabutylammonium tetrafluoroborate, electrolysis; (**f**) *N*-hydroxy-(substituted) benzimidoyl chloride, DMF, Et_3_N, 0-r.t.; (**g**) Pt and Fe electrodes, MeOH, electrolysis.

The present work synthesized novel thiouracil-tethered triazole/isoxazole derivatives and studied their anticancer activity. Triazole analogues (**5a**–**l**) were obtained by adding CuI to a stirred solution of substituted azides and intermediates (**4a**–**d**) in THF under basic conditions. Additionally, the synthesis of 1,2,3-triazoles was performed via electrochemical oxidation of copper foil cells as the working electrode and platinum as the counter electrode in tertiary butanol:water medium (1:1). The reaction gave a good yield in the presence of an analytical amount of tetrabutylammonium tetrafluoroborate (TBATFB) within 60 min. This method was highly useful for synthesizing 1,2,3-triazoles tethered with a bioactive scaffold (Scheme 1).

Isoxazole analogues (**6a**–**l**) were obtained via a cyclization reaction between *N*-hydroxy-(substituted) benzimidoyl chloride and intermediates (**4a**–**d**) in DMF under basic conditions. Moreover, the synthesis of isoxazole was conducted using the electrolysis method. Under constant current supplying with a density of 5.0 mA/cm^−2^ into a beaker-type undivided cell, *N*-hydroxy-(substituted) benzimidoyl chlorides and alkynes (**4a**–**d**) were reacted by using Pt-plate as the working electrode and an iron rod as the cathode in methanolic medium, and the desired isoxazoles were obtained in good yield when compared to the abovementioned conventional method (Scheme 1).

**Scheme 2:**
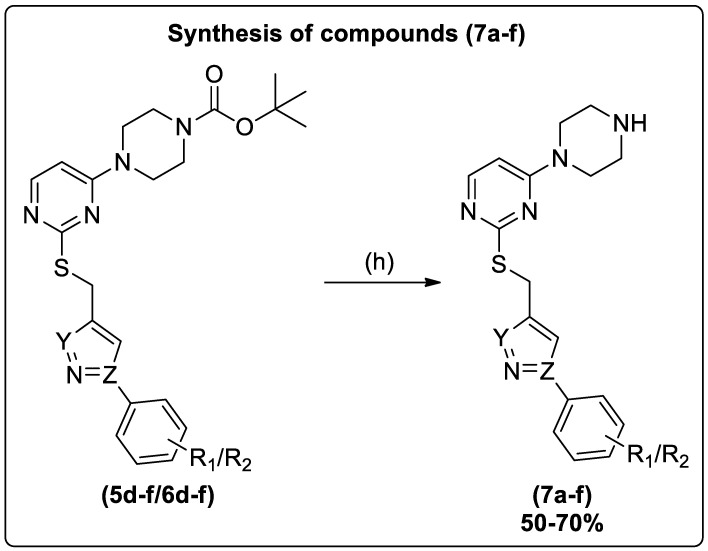
Reagents and conditions: (**h**) (**5d**–**f**/**6d**–**f**), DCM, CF_3_COOH, r.t., 1 h.

**Scheme 3:**
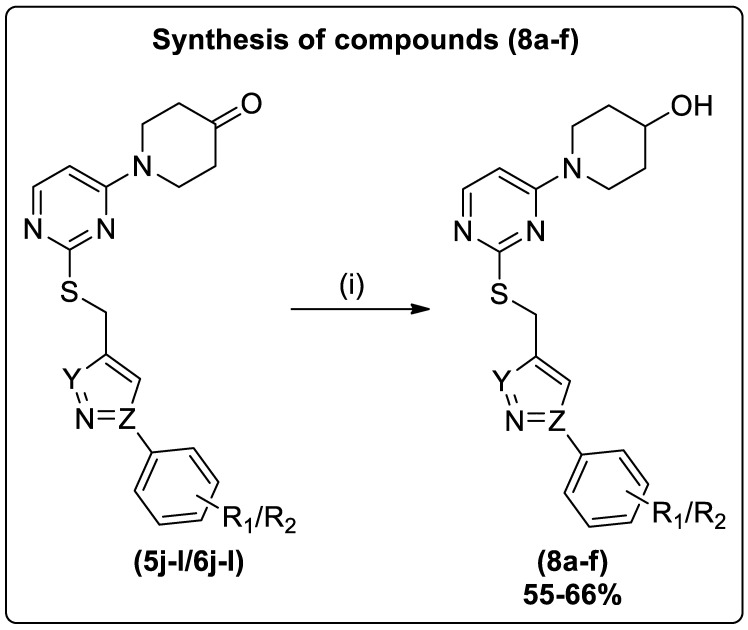
Reagents and conditions: (**i**) (**5j**–**l**/**6j**–**l**), THF, NaBH_4_, r.t., 1.5 h.

Furthermore, the *N*-boc deprotection of compounds (**5d**–**f/6d**–**f**) was accomplished upon treatment with trifluoroacetic acid in DCM to yield (**7a**–**f**) (Scheme 2). The ketone group reduction in compounds (**5j**–**l/6j**–**l**) was made on treatment with sodium borohydride in THF to yield (**8a**–**f**) (Scheme 3).

### 2.2. Cytotoxicity of Thiouracil Tethered Triazole/Isoxazole Analogues on MCF-7 Cells

**Figure 3:**
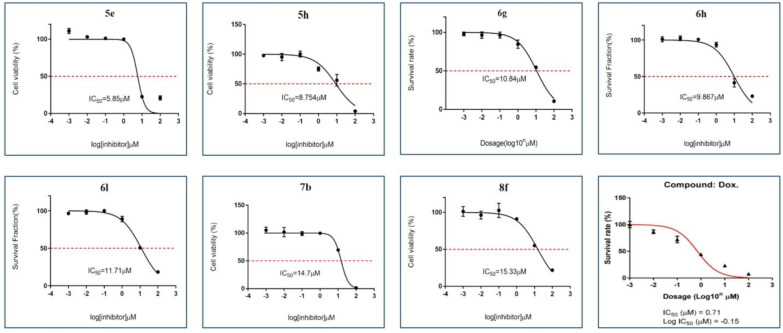
Effect of lead compounds **5e**, **5h**, **6g**, **6h**, **6l**, **7b**, and **8f** on MCF7 cell viability (black line), as determined by the Alamar Blue cell viability assay, with Doxorubicin as positive control (red line), as described in the Materials and Methods section. Error bars represent SD, and points (0, 0.01, 0.1, 1, 10, or 100 µM) represent the mean of three independent measurements after 72 h incubation of compounds. Error bars indicate an SD of 2.3. In Silico molecular interaction studies of compounds bearing isoxazole (**6l**) and triazole (**5h**) on HDAC7.

Initially, the novel thiouracil tethered triazole/isoxazole analogues were subjected to cell viability studies against human breast cancer (BC) (MCF-7) cells (Table 1), using the Alamar Blue assay [32,33,34]. For over four decades, numerous research groups have extensively utilized MCF-7, a BC cell line exhibiting a well-characterized molecular profile and dependence on estrogen, in drug development research [35,36]. The assay was performed at different concentrations (0, 0.01, 0.1, 1, 10, or 100 µM) for 72 h, and the positive control Doxorubicin inhibited the viability of MCF-7 cells with an IC_50_ value of 0.71 µM. The results of the assay displayed that compounds **5e**, **5h**, **6g**, **6h**, **6l**, **7b**, or **8f** produced loss of viability of MCF-7 cells with varied IC_50_ values of 5.85, 8.754, 10.84, 9.867, 11.71, 14.70, or 15.33 µM, respectively (Figure 3). In addition, the active compounds are non-toxic to immortalized breast cancer cells (MCF-10A) (Supplementary Materials). Among the tested isoxazole and triazole-tethered thiouracil molecules, triazole-based compounds bearing 3,4-dichloro phenyl group in **5c**, **5e**, **5h**, or **7b** inhibited the human BC cells with an IC_50_ value of 11.31, 5.85, 8.754, or 14.7 uM, respectively, when compared to less active compounds that belonged to methoxy, methyl, or hydroxy-phenyl groups. Moreover, in isoxazoles molecules, those bearing methoxyphenyl-substitution in compounds **6d**, **6h**, **6g**, or **7d** were found to be cytotoxic to human BC cells, with IC_50_ values of 15.96, 10.84, 9.86, or 14.8 μM, respectively. On the other hand, methylphenyl-substitution-to-isoxazole-based compounds such as **6i**, **6l**, **7f**, or **8f** showed considerable IC_50_ values of 43.27, 11.71, 14.83, or 15.33 μM, respectively. So, the SAR data revealed that the compounds having triazole and 3,4-dichloro phenyl substitutions favored higher cytotoxicity. In contrast, the isoxazole compound and methoxy or methyl-phenyl-substitution showed better bioactivity against human BC cells.

We previously reported that the isoxazole-and-triazole-bearing group targets HDAC, which showed a similar binding pattern of HDACis at the active site of an enzyme and formed multiple molecular interactions in the hydrophobic region [26]. In continuation of development of new HDACis, we carried out the molecular docking analysis of lead structures (**6l**) 1-(2-(((3-(*p*-tolyl) isoxazol-5-yl)methyl)thio) pyrimidin-4-yl) piperidin-4-one and (**5h**) 4-(2-(((1-(3,4-dichlorophenyl)-1*H*-1,2,3-triazol-4-yl)methyl)thio) pyrimidin-4-yl) morpholine, using the co-crystal structure of HDAC7 (PDB ID: 3ZNR), which bound to the isoxazole and triazole ligands, respectively.

We initially retrieved HDAC7 co-crystal structure from RCBS PDB (PDB ID: 3ZNR) and used it for docking purposes [37]. The Scripps Research Institute’s Auto-Dock4 tools (ADT)v1.5.6 was used for docking. The protein structure HDAC7, compounds **6l** and **5h**, and co-crystal ligand (TMP269) were prepared using discovery studio BIOVIA Discovery Studio 2021 client and Avogadro, and later it was docked using AutoDock tools (ADT).

**Figure 4:**
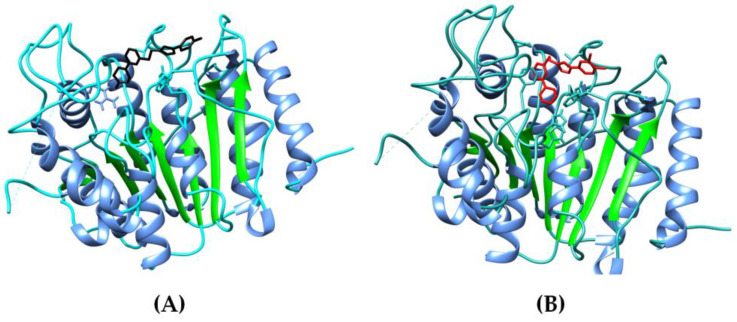
Cartoon representation of docked compounds **6l** (black) (**A**) and **5h** (red) (**B**) towards the active site of HDAC7.

The isoxazole derivative (**6l**) exhibited a binding energy of −6.60 kcal/mol, indicating a strong affinity for the HDAC7 binding site. Similarly, the triazole derivative (**5h**) demonstrated a slightly higher binding energy of −6.42 kcal/mol, suggesting an even more robust interaction with the target protein. Both compounds displayed favourable binding energies, indicating their potential as HDAC7 inhibitors, and cartoon representations of docked compounds are depicted in Figure 4A,B. In comparison, the co-crystal ligand (TMP269) exhibited a binding energy of −6.19 kcal/mol, which falls within the range of the isoxazole and triazole compounds. This suggests that the co-crystal ligand also possesses a significant binding affinity for HDAC7.

**Figure 5:**
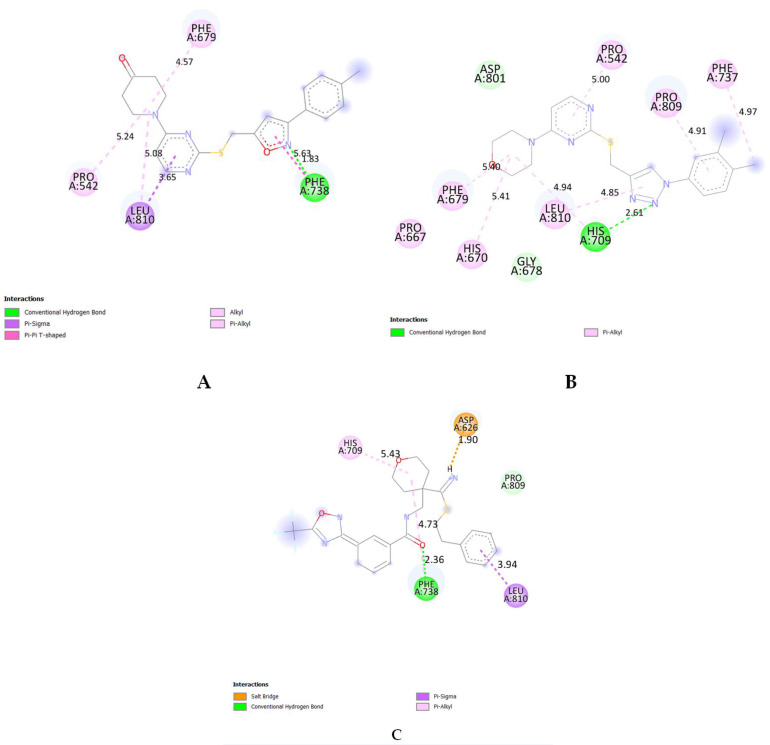
Representation of 2D structures of compounds **6l** (**A**), **5h** (**B**), and co-crystal ligand (TMP269) (**C**) interacting with the active site of HDAC7 (PDB ID: 3ZNR). Doted lines represents bond distances.

An analysis of the binding pocket revealed critical interactions for the isoxazole, triazole, and co-crystal ligands. The isoxazole compound (**6l**) formed hydrogen bonds with residue PHE-738 with a bond distance of 1.83 Å, stabilizing its binding within the pocket (Figure 5A). Moreover, **6l** had π- sigma bond formation with the residue LEU-810 with a bond distance of 3.65 Å, and π-π T-shaped bonds formed with residue PHE-738 with a bond distance of 5.63 Å, and alkyl bond formed with residues LEU-810 and PRO-542, respectively. The triazole compound (**5h**) displayed hydrogen bond interactions with residue HIS-709 having a bond distance of 2.69 Å, and π-alkyl bonds were formed with residues PRO-242, HIS-670, PHE-679, PHE-737, PRO-809, and LEU-810, contributing to its strong binding (Figure 5B). The co-crystal ligand (TMP269) demonstrated hydrogen bond interactions with residues PHE-738 with a bond distance of 2.36 Å, and a π-alkyl bond formed with residues HIS-709 and PHE-738 with bond distances of 5.43 Å and 4.73 Å, respectively. Furthermore, TMP269 had a π-sigma bond and salt bridge interactions with residues LEU-810 and ASP-626, respectively (Figure 5C). These interactions play a crucial role in maintaining the stability and specificity of the ligand–protein complex.

The results of the molecular docking study highlight the good binding affinity of both the isoxazole (**6l**) and triazole (**5h**) compounds towards the HDAC7 binding site, with binding energies of −6.60 and −6.42 kcal/mol, respectively. These findings suggest that both compounds have the potential to be effective inhibitors of HDAC7. Furthermore, the co-crystal ligand (TMP269) exhibited a favourable binding energy of −6.19 kcal/mol, indicating its potency as an HDAC7 inhibitor. The critical interactions observed within the binding pocket emphasize the role of specific residues in facilitating ligand–protein interactions for all three compounds.

**Figure 6:**
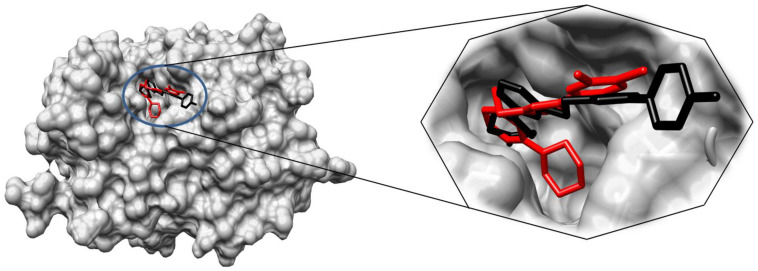
Three-dimensional surface image and its enlarged view of docked compounds **6l** (black) and **5h** (represented in red color) with active site groove of HDAC7.

These results provide valuable insights into the potential therapeutic utility of the isoxazole (**6l**) and triazole (**5h**) compounds as HDAC7 inhibitors—also, the 3D surface view of **6l** and **5h** is represented in Figure 6.

## 3. Materials and Methods

All chemicals and solvents were purchased from Sigma-Aldrich (Bangalore, India). Pre-coated silica gel TLC plates monitored the completion of the reaction. An Agilent mass spectrophotometer was used to record the mass of the synthesized compounds. ^1^H and ^13^C NMR were recorded on Bruker (400 MHz) and Jeol NMR spectrophotometers (500 MHz). TMS was used as an internal standard, and deuterated DMSO and chloroform were used as solvents. Chemical shifts are expressed as ppm.

### 3.1. General Procedure for the Synthesis of Intermediates *(**4a**–**d**)*

Potassium hydroxide (1.4 mmol) was dissolved in ethanol:water (1:1) in an RB flask, and 2-thiouracil (**1**) (1 mmol) was added. After 5 min, propargyl bromide (1.5 mmol) was added and refluxed at 50 °C for 45 min. The completion of the reaction was monitored by TLC (EtOAc:Hexane) (3:7), and ethanol was evaporated under high vacuum pressure. The crude was washed with 10% bicarbonate solution, ethanol, and diethyl ether to yield pure 2-(prop-2-yn-1-ylthio) pyrimidin-4-ol (**2**) [38]. Reactant (**2**) was refluxed in POCl_3_ for 20 min after completion of the reaction [TLC: (EtOAc:Hexane) (1:9)]; the reaction mixture was quenched in ice-cold water and neutralized by potassium carbonate [39]. Then, the reaction mass was extracted to the ethyl acetate layer and concentrated using a rotary evaporator to afford 4-chloro-2-(prop-2-yn-1-ylthio)pyrimidine (**3**). For Compound (**3**) (1 mmol), substituted piperazines/morpholine/4-piperidinone (1 mmol) were dissolved in acetone and refluxed in the presence of base triethylamine (Et_3_N) (2 mmol) for 8–10 h [40]. TLC was monitored for the completion of the reaction, and the crude intermediates (**4a**–**d**) were purified by column chromatography (EtOAc:Hexane) (2:8) (Scheme 1).

### 3.2. General Procedure for the Synthesis of Thiouracil Tethered Triazoles *(**5a**–**l**)*

A. Conventional method: To a stirred solution of substituted azides (1.2 mmol), (**4a**–**d**) (1 mmol) in tetrahydrofuran (THF), CuI (20 mol%), and triethylamine (2.0 mmol) was added, and the reaction mixture was stirred at room temperature for 3–5 h. After completion of the reaction [TLC: (EtOAc:Hexane) (4:6)], THF was distilled off, and the crude was purified by column chromatography to afford thiouracil tethered triazole derivatives (**5a**–**l**) (Scheme 1) [41].

B. Electrochemical method: In the presence of copper (Cu) foil and platinum (Pt) electrodes, the azides and alkynes dissolved in *tert*-butyl alcohol and water (1:1) medium were subjected to a 0.3 voltage of current in the catalytic amount of TBATFB (0.1 mmol) for 1 h. The reaction was monitored by TLC [(EtOAc:Hexane) (4:6)], and soon after the completion of the reaction, the 1,2,3-triazoles were extracted in ethyl acetate and purified using column chromatography (Scheme 1).

### 3.3. General Procedure for the Synthesis of Thiouracil Tethered Isoxazoles *(**6a**–**l**)*

A. Conventional method: To a stirred solution of *N*-hydroxy-(substituted)benzimidoyl chloride (1 mmol) in dimethylformamide (DMF), triethylamine (2.5 mmol) was added slowly at 0–4 °C. After 10 min, compound (**4a**–**d**) was added and stirred overnight at room temperature. After completion of the reaction TLC: (EtOAc:Hexane) (3:7)], water was added to the above reaction mixture, and the crude obtained was filtered. The crude was purified by column chromatography to yield pure thiouracil tethered isoxazole derivatives (**6a**–**l**) (Scheme 1) [42].

B. Electrochemical method: Beaker-type undivided cells containing platinum (Pt) and iron (Fe) rod as electrodes and methanol as media were kept at room temperature. *N*-hydroxy-(substituted) benzimidoyl chlorides and alkynes were added to the solution, and the current 5.0 mA/cm^−2^ was passed to the reaction mixture. The reaction was stirred for 60 min under stirring, and the reaction was monitored by TLC [(EtOAc:Hexane) (3:7)] and was terminated when the substrate was consumed. The products were isolated as mentioned in the abovementioned procedure (Scheme 1).

### 3.4. General Procedure for the Synthesis of Compounds *(**7a**–**f**)*

To a stirred solution of compounds (**5d**–**f/6d**–**f**) in dichloromethane (DCM), 0.5 mL of trifluoroacetic acid was added slowly at 0–4 °C, and the reaction mixture was stirred at room temperature for 1 h [43]. After completion of the *N*-boc deprotection [TLC: (MeOH: EtOAc) (1:9)], the solvent was distilled off using a rotary evaporator. The residues were neutralized by a 20% K_2_CO_3_ solution, extracted with an ethyl acetate layer, and dried over Na_2_SO_4_. Then, ethyl acetate was concentrated in a rotary evaporator and recrystallized with the appropriate solvent to afford the target compounds (**7a**–**f**) (Scheme 2).

### 3.5. General Procedure for the Synthesis of Compounds *(**8a**–**f**)*

To a stirred solution of compounds (**5j**–**l/6j**–**l**) (1 mmol) in THF, sodium borohydride (1.2 mmol) was added, and the reaction mass was stirred at room temperature for 1.5 h. After completion of ketone group reduction [TLC: (EtOAc: Hexane) (7:3)], the solvent was distilled off using a rotary evaporator. The residues were neutralized by the slow addition of water, extracted with an ethyl acetate layer, and dried over Na_2_SO_4_. Then, ethyl acetate was distilled off, and the crude was purified through column chromatography or recrystallized with the appropriate solvents to afford the final compounds (**8a**–**f**) (Scheme 3).

### 3.6. 4-Chloro-2-(prop-2-yn-1-ylthio)pyrimidine *(**3**)*

LCMS (ESI): *m*/*z* calcd. for C_7_H_5_ClN_2_S: 184.6460, found = 185.0446 [M + H]^+^, 187.0405 [M + 2H]^+^.

### 3.7. 4-(4-(2,3-Dichlorophenyl) piperazin-1-yl)-2-(((1-(4-methoxyphenyl)-1H-1,2,3-triazol-4-yl)methyl)thio) pyrimidine *(**5a**)*

Off-white solid; 55% yield; mp: 154–156 °C; ^1^H NMR (CDCl_3_, 500 MHz): *δ* 8.05 (d, *J* = 6.0 Hz, 1H, pyrimidine-H), 7.87 (s, 1H, triazole-H), 7.56 (d, *J* = 9.0 Hz, 2H, -C_6_H_4_), 7.18 (dd, *J* = 8.0, 1.5 Hz, 1H, -C_6_H_3_), 7.13 (t, *J* = 8.0 Hz, 1H, -C_6_H_3_), 6.97 (d, *J* = 9.0 Hz, 2H, -C_6_H_4_), 6.88 (dd, *J* = 8.0, 1.5 Hz, 1H, pyrimidine-H), 6.24 (d, *J* = 6.0 Hz, 1H, -C_6_H_3_), 4.49 (s, 2H, -CH_2_-), 3.83 (s, 3H, -OCH_3_), 3.79 (s, 4H, piperazine-H), 3.04 (t, *J* = 5.0 Hz, 4H, piperazine-H); ^13^C NMR (CDCl_3_, 126 MHz): *δ* 169.9, 161.2, 159.8, 156.1, 150.7, 146.7, 134.3, 130.6, 127.8, 127.7, 125.3, 122.1, 120.7, 118.8, 114.8, 99.1 (Ar-C), 55.7 (-OCH_3_), 51.1, 44.1 (piperazine-C), 25.8 (-CH_2_-).

### 3.8. 2-(((1-(3,4-Dichlorophenyl)-1H-1,2,3-triazol-4-yl)methyl)thio)-4-(4-(2,3-dichlorophenyl)piperazin-1-yl) pyrimidine *(**5b**)*

Light yellow solid; 44% yield; mp: 172–174 °C; ^1^H NMR (CDCl_3_, 500 MHz): *δ* 8.08 (d, *J* = 6.0 Hz, 1H, pyrimidine-H), 7.97 (s, 1H, triazole-H), 7.91–7.81 (m, 1H, -C_6_H_3_-triazole), 7.58 (d, *J* = 1.0 Hz, 2H, -C_6_H_3_-triazole), 7.21 (dd, *J* = 8.0, 1.5 Hz, 1H, -C_6_H_3_-piperazine), 7.16 (t, *J* = 8.0 Hz, 1H, -C_6_H_3_-piperazine), 6.91 (dd, *J* = 8.0, 1.5 Hz, 1H, pyrimidine-H), 6.27 (d, *J* = 6.0 Hz, 1H, -C_6_H_3_-piperazine), 4.50 (s, 2H, -CH_2_-), 3.81 (s, 4H, piperazine-H), 3.06 (t, *J* = 5.0 Hz, 4H, piperazine-H); ^13^C NMR (CDCl_3_, 126 MHz): *δ* 169.5, 161.1, 156.0, 150.5, 147.6, 136.0, 134.1, 133.8, 132.6, 131.4, 127.6, 127.5, 125.2, 122.0, 120.2, 119.2, 118.6, 99.0 (Ar-C), 50.9, 44.0 (piperazine-C), 25.5 (-CH_2_-).

### 3.9. 4-(4-(((4-(4-(2,3-Dichlorophenyl) piperazin-1-yl) pyrimidin-2-yl)thio)methyl)-1H-1,2,3-triazol-1-yl)phenol *(**5c**)*

Brown solid; 48% yield; ^1^H NMR (DMSO, 500 MHz): 9.40 (s, 1H, -OH-C_6_H_4_), 8.27 (s, 1H, triazole-H), 8.08 (d, *J* = 6.0 Hz, 1H, pyrimidine-H), 7.57 (d, *J* = 8.5 Hz, 2H, -C_6_H_4_), 7.34–7.23 (m, 2H, -C_6_H_3_), 7.18–7.06 (m, 1H, -C_6_H_3_), 6.91 (d, *J* = 9.0 Hz, 2H, -C_6_H_4_), 6.55 (d, *J* = 6.0 Hz, 1H, pyrimidine-H), 4.46 (s, 2H, -CH_2_-), 3.86–3.74 (m, 4H, piperazine-H), 3.15–3.03 (m, 4H, piperazine-H); ^13^C NMR (DMSO, 126 MHz): *δ* 168.5, 160.7, 157.3, 155.4, 150.4, 144.4, 132.4, 128.6, 127.9, 126.0, 124.3, 121.5, 120.7, 119.4, 115.6, 99.2 (Ar-C), 50.2, 43.4 (piperazine-C), 24.6 (-CH_2_-); LCMS (ESI): *m*/*z* calcd. for C_23_H_21_Cl_2_N_7_OS: 514.4301, found = 514.1631 [M + H]^+^, 516.1606 [M + 2H]^+^.

### 3.10. Tert-butyl 4-(2-(((1-(4-methoxyphenyl)-1H-1,2,3-triazol-4-yl)methyl)thio)pyrimidin-4-yl) piperazine-1-carboxylate *(**5d**)*

Yellow solid; 60% yield; mp: 94–96 °C; ^1^H NMR (CDCl_3_, 500 MHz): *δ* 8.02 (d, *J* = 6.0 Hz, 1H, pyrimidine-H), 7.84 (s, 1H, triazole-H), 7.55 (d, *J* = 9.0 Hz, 2H, -C_6_H_4_), 6.96 (d, *J* = 9.0 Hz, 2H, -C_6_H_4_), 6.17 (d, *J* = 6.5 Hz, 1H, pyrimidine-H), 4.45 (s, 2H, -CH_2_-), 3.82 (s, 3H, -OCH_3_), 3.59 (s, 4H, piperazine-H), 3.45 (s, 4H, piperazine-H), 1.44 (s, 9H, -(CH_3_)_3_); ^13^C NMR (CDCl_3_, 126 MHz): *δ* 169.8, 161.1, 159.8, 156.1, 154.7 (-COO), 146.6, 130.6, 122.2, 120.6, 114.8, 99.0 (Ar-C), 80.4 (-C-(CH_3_)_3_), 55.7 (-OCH_3_), 43.6 (piperazine-C), 28.5 (CH_3_)_3_, 25.8 (-CH_2_-).

### 3.11. Tert-butyl 4-(2-(((1-(3,4-dichlorophenyl)-1H-1,2,3-triazol-4-yl)methyl)thio)pyrimidin-4-yl) piperazine-1-carboxylate *(**5e**)*

Yellow solid; 54% yield; mp: 153–155 °C; ^1^H NMR (CDCl_3_, 500 MHz): *δ* 8.04 (d, *J* = 6.5 Hz, 1H, pyrimidine-H), 7.93 (s, 1H, triazole-H), 7.84 (d, *J* = 1.0 Hz, 1H, -C_6_H_3_), 7.56 (s, 2H, -C_6_H_3_), 6.19 (d, *J* = 6.0 Hz, 1H, pyrimidine-H), 4.46 (s, 2H, -CH_2_-), 3.60 (s, 4H, piperazine-H), 3.47 (d, *J* = 4.5 Hz, 4H, piperazine-H), 1.46 (s, 9H, -(CH_3_)_3_); ^13^C NMR (CDCl_3_, 126 MHz): *δ* 169.6, 161.1, 156.1, 154.7 (-COO), 147.7, 136.1, 134.0, 132.8, 131.5, 122.2, 120.3, 119.4, 99.1 (Ar-C), 80.5 (-C-(CH_3_)_3_), 43.6 (piperazine-C), 28.5 (CH_3_)_3_, 25.6 (-CH_2_-).

### 3.12. Tert-butyl 4-(2-(((1-(4-hydroxyphenyl)-1H-1,2,3-triazol-4-yl)methyl)thio)pyrimidin-4-yl) piperazine-1-carboxylate *(**5f**)*

Pink solid; 52% yield; mp: 96–98 °C; ^1^H NMR (CDCl_3_, 500 MHz): *δ* 7.84 (d, *J* = 6.5 Hz, 1H, pyrimidine-H), 7.64 (s, 1H, OH, -OH-C_6_H_4_), 7.29 (d, *J* = 9.0 Hz, 2H, -C_6_H_4_), 7.07 (s, 1H, triazole-H), 6.76 (d, *J* = 9.0 Hz, 2H, -C_6_H_4_), 6.01 (d, *J* = 6.0 Hz, 1H, pyrimidine-H), 4.28 (s, 2H, (-CH_2_-)), 3.44 (s, 4H, piperazine-H), 3.36–3.24 (m, 4H, piperazine-H), 1.28 (s, 9H, -(CH_3_)_3_); ^13^C NMR (CDCl_3_, 126 MHz): *δ* 169.2, 160.7, 156.9, 155.4, 154.5 (-COO), 146.3, 129.7, 122.0, 120.4, 116.2, 98.8 (Ar-C), 80.3 (-C-(CH_3_)_3_), 43.4 (piperazine-C), 28.2 (CH_3_)_3_, 25.4 (-CH_2_-).

### 3.13. 4-(2-(((1-(4-Methoxyphenyl)-1H-1,2,3-triazol-4-yl)methyl)thio)pyrimidin-4-yl)morpholine *(**5g**)*

Yellow solid; 60% yield; mp: 140–142 °C; ^1^H NMR (CDCl_3_, 500 MHz): *δ* 8.10–7.97 (m, 1H, pyrimidine-H), 7.85 (s, 1H, triazole-H), 7.62–7.50 (m, 2H, -C_6_H_4_), 6.97 (d, *J* = 8.5 Hz, 2H, -C_6_H_4_), 6.17 (d, *J* = 6.0 Hz, 1H, pyrimidine-H), 4.47 (s, 2H, -CH_2_-), 3.84 (d, *J* = 0.5 Hz, 3H, -OCH_3_), 3.78–3.66 (m, 4H, morpholine-H), 3.58 (s, 4H, morpholine-H); ^13^C NMR (CDCl_3_, 126 MHz): *δ* 169.9, 161.3, 159.8, 156.1, 146.7, 130.6, 122.2, 120.6, 114.8, 98.9 (Ar-C), 66.5 (morpholine-C), 55.7 (-OCH_3_), 44.1 (morpholine-C), 25.8 (-CH_2_-).

### 3.14. 4-(2-(((1-(3,4-Dichlorophenyl)-1H-1,2,3-triazol-4-yl)methyl)thio) pyrimidin-4-yl)morpholine *(**5h**)*

Off-white solid; 58% yield; mp: 158–160 °C; ^1^H NMR (CDCl_3_, 500 MHz): *δ* 7.80 (d, *J* = 6.5 Hz, 1H, pyrimidine-H), 7.68 (s, 1H, triazole-H), 7.64–7.53 (m, 1H, -C_6_H_3_), 7.36–7.26 (m, 1H, -C_6_H_3_), 7.00 (s, 1H, -C_6_H_3_), 5.93 (d, *J* = 6.5 Hz, 1H), pyrimidine-H, 4.21 (d, *J* = 0.5 Hz, 2H, -CH_2_-), 3.53–3.42 (m, 4H, morpholine-H), 3.33 (s, 4H, morpholine-H); ^13^C NMR (CDCl_3_, 126 MHz): *δ* 169.5, 161.2, 155.9, 147.5, 136.0, 133.8, 132.6, 131.3, 122.0, 120.1, 119.2, 98.8 (Ar-C), 66.3, 43.9 (morpholine-C), 25.4 (-CH_2_-).

### 3.15. 4-(4-(((4-Morpholinopyrimidin-2-yl)thio)methyl)-1H-1,2,3-triazol-1-yl) phenol *(**5i**)*

Brown solid; 55% yield; mp: 216–218 °C; ^1^H NMR (DMSO, 500 MHz): *δ* 9.89 (s, 1H, pyrimidine-H), 8.44 (s, 1H, triazole-H), 7.59 (d, *J* = 9.0 Hz, 2H, -C_6_H_4_), 6.86 (d, *J* = 9.0 Hz, 2H, -C_6_H_4_), 6.53 (d, *J* = 6.0 Hz, 1H, pyrimidine-H), 4.37 (s, 2H, -CH_2_-), 3.65–3.54 (m, 4H, morpholine-H), 3.55 (d, *J* = 2.5 Hz, 4H, morpholine-H); ^13^C NMR (DMSO, 126 MHz): *δ* 169.2, 161.5, 158.2, 156.3, 145.4, 129.3, 122.4, 121.7, 116.5, 100.1 (Ar-C), 66.3, 44.2 (morpholine-C), 25.4 (-CH_2_-).

### 3.16. 1-(2-(((1-(4-Methoxyphenyl)-1H-1,2,3-triazol-4-yl)methyl)thio)pyrimidin-4-yl)piperidin-4-one *(**5j**)*

Brown solid; 58% yield; mp: 99–101 °C; ^1^H NMR (DMSO, 400 MHz): *δ* 8.58 (s, 1H, triazole-H), 8.11 (d, *J* = 6.0 Hz, 1H, pyrimidine-H), 7.78 (d, *J* = 8.8 Hz, 2H, -C_6_H_4_), 7.11 (d, *J* = 9.2 Hz, 2H, -C_6_H_4_), 6.66 (d, *J* = 6.0 Hz, 1H, pyrimidine-H), 4.46 (s, 2H, -CH_2_-), 3.92 (s, 4H, piperidone-H), 3.82 (s, 3H, -OCH_3_), 2.50–2.37 (m, 4H, piperidone-H); ^13^C NMR (DMSO, 101 MHz): *δ* 207.9 (-CO), 169.2, 160.9, 159.6, 156.4, 145.6, 130.5, 122.1, 121.7, 115.3, 100.0 (Ar-C), 56.0 (-OCH_3_), 42.3 (piperidone-C), 25.4 (-CH_2_-).

### 3.17. 1-(2-(((1-(3,4-Dichlorophenyl)-1H-1,2,3-triazol-4-yl)methyl)thio)pyrimidin-4-yl)piperidin-4-one *(**5k**)*

Yellow solid; 56% yield; mp: 148–150 °C; ^1^H NMR (DMSO, 400 MHz): *δ* 8.80 (s, 1H, triazole-H), 8.25 (d, *J* = 2.0 Hz, 1H, -C_6_H_3_), 8.11 (d, *J* = 6.0 Hz, 1H, pyrimidine-H), 7.95 (dd, *J* = 8.8, 2.4 Hz, 1H, -C_6_H_3_), 7.84 (d, *J* = 8.8 Hz, 1H, -C_6_H_3_), 6.66 (d, *J* = 6.0 Hz, 1H, pyrimidine-H), 4.47 (s, 2H, -CH_2_-), 3.91 (s, 4H, piperidone-H), 2.43 (t, *J* = 6.0 Hz, 4H, piperidone-H); ^13^C NMR (DMSO, 101 MHz): *δ* 207.8 (-CO), 169.1, 160.9, 156.4, 146.5, 136.6, 132.8, 132.2, 131.3, 122.1, 122.0, 120.4, 100.1 (Ar-C), 42.3 (piperidone-C), 25.2 (-CH_2_-).

### 3.18. 1-(2-(((1-(4-Hydroxyphenyl)-1H-1,2,3-triazol-4-yl)methyl)thio)pyrimidin-4-yl)piperidin-4-one *(**5l**)*

Yellow solid; 55% yield; ^1^H NMR (DMSO, 400 MHz): *δ* 9.96 (s, 1H, -OH-C_6_H_4_), 8.49 (s, 1H, triazole-H), 8.11 (d, *J* = 6.0 Hz, 1H, pyrimidine-H), 7.63 (d, *J* = 8.8 Hz, 2H, -C_6_H_4_), 6.91 (d, *J* = 8.8 Hz, 2H, -C_6_H_4_), 6.66 (d, *J* = 6.0 Hz, 1H, pyrimidine-H), 4.44 (s, 2H, -CH_2_-), 3.92 (s, 4H, piperidone-H), 2.44 (t, *J* = 6.0 Hz, 4H, piperidone-H); ^13^C NMR (DMSO, 101 MHz): *δ* 207.9 (-CO), 169.3, 160.9, 158.1, 156.4, 145.4, 129.2, 122.3, 121.6, 116.5, 100.1 (Ar-C), 42.3 (piperidone-C), 25.4 (-CH_2_-).

### 3.19. 5-(((4-(4-(2,3-Dichlorophenyl) piperazin-1-yl) pyrimidin-2-yl)thio)methyl)-3-(4-methoxyphenyl) isoxazole *(**6a**)*

Yellow solid; 48% yield; mp: 134–136 °C; ^1^H NMR (CDCl_3_, 500 MHz): *δ* 8.04 (d, *J* = 6.5 Hz, 1H, pyrimidine-H), 7.68 (d, *J* = 9.0 Hz, 2H, -C_6_H_4_), 7.17 (dd, *J* = 8.0, 1.0 Hz, 1H, -C_6_H_3_), 7.10 (t, *J* = 8.0 Hz, 1H, -C_6_H_3_), 6.92 (d, *J* = 9.0 Hz, 2H, -C_6_H_4_), 6.83 (dd, *J* = 8.0, 1.0 Hz, 1H, -C_6_H_3_), 6.49 (s, 1H, isoxazole-H), 6.24 (d, *J* = 6.0 Hz, 1H, pyrimidine-H), 4.39 (s, 2H, -CH_2_-), 3.82 (s, 3H, -OCH_3_), 3.77 (s, 4H, piperazine-H), 3.08–2.96 (m, 4H, piperazine-H); ^13^C NMR (CDCl_3_, 126 MHz): *δ* 170.9, 168.9, 162.2, 161.2, 161.0, 156.1, 150.6, 134.2, 128.2, 127.8, 127.6, 125.3, 121.7, 118.8, 114.3, 100.2, 99.3 (Ar-C), 55.4 (-OCH_3_), 51.0, 44.2 (piperazine-C), 26.0 (-CH_2_-); LCMS (ESI): *m*/*z* calcd. for C_25_H_23_Cl_2_N_5_O_2_S: 528.4534, found = 528.2081 [M + H]^+^, 530.2043 [M + 2H]^+^.

### 3.20. 5-(((4-(4-(2,3-Dichlorophenyl) piperazin-1-yl) pyrimidin-2-yl)thio)methyl)-3-(3,4-dimethoxy phenyl)isoxazole *(**6b**)*

Yellow solid; 40% yield; mp: 126–128 °C; ^1^H NMR (CDCl_3_, 500 MHz): *δ* 8.04 (d, *J* = 6.0 Hz, 1H, pyrimidine-H), 7.36 (d, *J* = 1.5 Hz, 1H, -C_6_H_3_-isoxazole), 7.17 (d, *J* = 8.0 Hz, 1H, -C_6_H_3_-isoxazole), 7.09 (t, *J* = 8.0 Hz, 1H, -C_6_H_3_-piperazine), 6.95–6.82 (m, 2H, -C_6_H_3_-piperazine), 6.82 (d, *J* = 8.0 Hz, 1H, -C_6_H_3_-isoxazole), 6.51 (s, 1H, isoxazole-H), 6.24 (d, *J* = 6.5 Hz, 1H, pyrimidine-H), 4.39 (s, 2H, -CH_2_-), 3.90 (s, 6H, (OCH_3_)_2_), 3.77 (s, 4H, piperazine-H), 3.02 (t, *J* = 4.5 Hz, 4H, piperazine-H); ^13^C NMR (CDCl_3_, 126 MHz): *δ* 171.0, 168.9, 162.3, 161.2, 156.1, 150.6, 149.3, 134.3, 127.6, 125.3, 121.8, 120.0, 118.7, 113.5, 112.5, 111.0, 109.2, 100.3, 99.3 (Ar-C), 56.1, 56.0 (-OCH_3_), 51.0, 44.2 (piperazine-C), 26.0(-CH_2_-); LCMS (ESI): *m*/*z* calcd. for C_26_H_25_Cl_2_N_5_O_3_S: 558.4794, found = 558.2141 [M + H]^+^, 560.2081 [M + 2H]^+^.

### 3.21. 5-(((4-(4-(2,3-Dichlorophenyl) piperazin-1-yl) pyrimidin-2-yl)thio)methyl)-3-(p-tolyl) isoxazole *(**6c**)*

Yellow solid; 52% yield; mp: 98–100 °C; ^1^H NMR (CDCl_3_, 500 MHz): *δ* 7.78 (d, *J* = 6.0 Hz, 1H, pyrimidine-H), 7.38 (d, *J* = 8.0 Hz, 2H, -C_6_H_4_), 6.95 (d, *J* = 7.5 Hz, 2H, -C_6_H_4_), 6.97–6.86 (m, 1H, -C_6_H_3_), 6.84 (t, *J* = 8.0 Hz, 1H, -C_6_H_3_), 6.57 (dd, *J* = 8.0, 1.5 Hz, 1H, -C_6_H_3_), 6.26 (s, 1H, isoxazole-H), 5.98 (d, *J* = 6.0 Hz, 1H, pyrimidine-H), 4.14 (s, 2H, -CH_2_-), 3.51 (s, 4H, piperazine-H), 2.76 (t, *J* = 5.0 Hz, 4H, piperazine-H), 2.10 (s, 3H, -CH_3_); ^13^C NMR (CDCl_3_, 126 MHz): *δ* 170.7, 168.6, 162.3, 160.9, 155.9, 150.4, 139.9, 134.0, 129.4, 127.5, 127.3, 126.4, 126.0, 125.0, 118.5, 100.1, 99.0 (Ar-C), 50.7, 43.9 (piperazine-C), 25.7 (-CH_2_-), 21.2 (-CH_3_).

### 3.22. Tert-butyl4-(2-(((3-(4-methoxyphenyl) isoxazol-5-yl)methyl)thio) pyrimidin-4-yl)piperazine-1-carboxylate *(**6d**)*

Yellow solid; 55% yield; mp: 120–122 °C; ^1^H NMR (CDCl_3_, 500 MHz): *δ* 8.03 (d, *J* = 6.5 Hz, 1H, pyrimidine-H), 7.68 (d, *J* = 8.5 Hz, 2H, -C_6_H_4_), 6.93 (d, *J* = 8.5 Hz, 2H, -C_6_H_4_), 6.47 (s, 1H, isoxazole-H), 6.19 (d, *J* = 6.5 Hz, 1H, pyrimidine-H), 4.38 (s, 2H, -CH_2_-), 3.82 (s, 3H, -OCH_3_), 3.59 (s, 4H, piperazine-H), 3.47 (s, 4H, piperazine-H), 1.46 (s, 9H, -(CH_3_)_3_); ^13^C NMR (CDCl_3_, 126 MHz): *δ* 170.9, 168.9, 162.2, 161.1, 161.0, 156.2, 154.7 (-COO), 128.2, 121.7, 114.3, 100.3, 99.2 (Ar-C), 80.4 (-C-(CH_3_)_3_), 55.4 (-OCH_3_), 43.6 (piperazine-C), 28.5 (CH_3_)_3_, 25.9 (-CH_2_-); LCMS (ESI): *m*/*z* calcd. for C_24_H_29_N_5_O_4_S: 483.5832, found = 484.1700 [M + H]^+^.

### 3.23. Tert-butyl 4-(2-(((3-(3,4-dimethoxyphenyl) isoxazol-5-yl)methyl)thio)pyrimidin-4-yl) pipera zine-1-carboxylate *(**6e**)*

Brown solid; 52% yield; mp: 85–87 °C; ^1^H NMR (DMSO, 400 MHz): *δ* 8.07 (d, *J* = 6.4 Hz, 1H, pyrimidine-H), 7.46–7.32 (m, 2H, -C_6_H_3_), 7.04 (d, *J* = 8.4 Hz, 1H, -C_6_H_3_), 6.92 (s, 1H, isoxazole-H), 6.58 (d, *J* = 6.0 Hz, 1H, pyrimidine-H), 4.51 (s, 2H, -CH_2_-), 3.82 (s, 3H, -OCH_3_), 3.80 (s, 3H, -OCH_3_), 3.61 (s, 4H, piperazine-H), 3.44–3.32 (s, 4H, piperazine-H), 1.41 (s, 9H, -(CH_3_)_3_); ^13^C NMR (DMSO, 101 MHz): *δ* 171.1, 168.3, 162.3, 161.2, 156.4, 154.3, 150.8, 149.5, 121.5, 120.0, 112.3, 110.0, 100.9, 100.3 (Ar-C), 79.6 (-C-(CH_3_)_3_), 56.0, 56.0 (-OCH_3_), 43.5 (piperazine-C), 28.5 (CH_3_)_3_, 25.5 (-CH_2_-); LCMS (ESI): *m*/*z* calcd. for C_25_H_31_N_5_O_5_S: 513.6091, found = 514.2142 [M + H]^+^.

### 3.24. Tert-butyl 4-(2-(((3-(p-tolyl) isoxazol-5-yl)methyl)thio) pyrimidin-4-yl)piperazine-1-carbox-ylate *(**6f**)*

Off-white solid; 60% yield; mp: 141–143 °C; ^1^H NMR (CDCl_3_, 500 MHz): *δ* 7.83 (d, *J* = 6.5 Hz, 1H, pyrimidine-H), 7.43 (d, *J* = 8.0 Hz, 2H, -C_6_H_4_), 7.01 (d, *J* = 8.0 Hz, 2H, -C_6_H_4_), 6.30 (s, 1H, isoxazole-H), 5.99 (d, *J* = 6.5 Hz, 1H, pyrimidine-H), 4.19 (s, 2H, -CH_2_-), 3.39 (s, 4H, piperazine-H), 3.27 (s, 4H, piperazine-H), 2.16 (s, 3H, -CH_3_), 1.26 (s, 9H, -(CH_3_)_3_); ^13^C NMR (CDCl_3_, 126 MHz): *δ* 170.7, 168.7, 162.4, 160.9, 156.0, 154.5 (-COO), 140.0, 129.4, 126.5, 126.1, 100.2, 99.0 (Ar-C), 80.2 (-C-(CH_3_)_3_), 43.4 (piperazine-C), 28.3 (CH_3_)_3_, 25.7 (-CH_2_-), 21.3 (-CH_3_).

### 3.25. 4-(2-(((3-(4-Methoxyphenyl) isoxazol-5-yl)methyl)thio) pyrimidin-4-yl)morpholine *(**6g**)*

Yellow solid; 54% yield; mp: 104–106 °C; ^1^H NMR (CDCl_3_, 500 MHz): *δ* 8.03 (d, *J* = 6.5 Hz, 1H, pyrimidine-H), 7.68 (d, *J* = 9.0 Hz, 2H, -C_6_H_4_), 6.93 (d, *J* = 9.0 Hz, 2H, -C_6_H_4_), 6.47 (s, 1H, isoxazole-H), 6.17 (d, *J* = 6.0 Hz, 1H, pyrimidine-H), 4.38 (s, 2H, -CH_2_-), 3.82 (s, 3H, -OCH_3_), 3.78–3.66 (m, 4H, morpholine-H), 3.57 (s, 4H, morpholine-H); ^13^C NMR (CDCl_3_, 126 MHz): *δ* 170.8, 168.9, 162.2, 161.3, 161.0, 156.1, 128.2, 121.7, 114.3, 100.3, 99.1 (Ar-C), 66.5 (morpholine-C), 55.4 (-OCH_3_), 44.1 (morpholine-C), 25.9 (-CH_2_-); LCMS (ESI): *m*/*z* calcd. for C_19_H_20_N_4_O_3_S: 384.4521, found = 385.1061 [M + H]^+^.

### 3.26. 4-(2-(((3-(3,4-Dimethoxyphenyl) isoxazol-5-yl)methyl)thio) pyrimidin-4-yl)morpholine *(**6h**)*

Yellow solid; 48% yield; mp: 118–120 °C; ^1^H NMR (CDCl_3_, 500 MHz): *δ* 8.03 (d, *J* = 6.0 Hz, 1H, pyrimidine-H), 7.35 (d, *J* = 1.0 Hz, 1H, -C_6_H_3_), 7.23 (dd, *J* = 8.5, 1.0 Hz, 1H, -C_6_H_3_), 6.88 (d, *J* = 8.5 Hz, 1H, -C_6_H_3_), 6.49 (s, 1H, isoxazole-H), 6.18 (d, *J* = 6.0 Hz, 1H, pyrimidine-H), 4.38 (s, 2H, -CH_2_-), 3.91 (s, 3H, -OCH_3_), 3.89 (s, 3H, -OCH_3_), 3.78–3.66 (m, 4H, morpholine-H), 3.57 (s, 4H, morpholine-H); ^13^C NMR (CDCl_3_, 126 MHz): *δ* 171.0, 168.9, 162.3, 161.4, 156.1, 150.6, 149.3, 121.8, 119.9, 111.1, 109.3, 100.3, 99.1 (Ar-C), 66.5 (morpholine-C), 56.1, 56.0 (-OCH_3_), 44.1 (morpholine-C), 25.9 (-CH_2_-); LCMS (ESI): *m*/*z* calcd. for C_20_H_22_N_4_O_4_S: 414.4781, found = 415.1629 [M + H]^+^.

### 3.27. 4-(2-(((3-(p-tolyl) isoxazol-5-yl)methyl)thio) pyrimidin-4-yl)morpholine *(**6i**)*

Off-white solid; 58% yield; mp: 128–130 °C; ^1^H NMR (CDCl_3_, 500 MHz): *δ* 8.04 (d, *J* = 6.0 Hz, 1H, pyrimidine-H), 7.63 (d, *J* = 8.0 Hz, 2H, -C_6_H_4_), 7.22 (d, *J* = 8.0 Hz, 2H, -C_6_H_4_), 6.50 (s, 1H, isoxazole-H), 6.18 (d, *J* = 6.0 Hz, 1H, pyrimidine-H), 4.39 (s, 2H, -CH_2_-), 3.78–3.66 (m, 4H, morpholine-H), 3.57 (s, 4H, morpholine-H), 2.37 (s, 3H, -CH_3_); ^13^C NMR (CDCl_3_, 126 MHz): *δ* 170.9, 168.9, 162.6, 161.3, 156.1, 140.2, 129.6, 126.7, 126.3, 100.4, 99.1 (Ar-C), 66.5, 44.1 (morpholine-C), 25.9 (-CH_2_-), 21.5 (-CH_3_).

### 3.28. 1-(2-(((3-(4-methoxyphenyl) isoxazol-5-yl)methyl)thio) pyrimidin-4-yl) piperidin-4-one *(**6j**)*

Yellow solid; 50% yield; mp: 125–127 °C; ^1^H NMR (DMSO, 400 MHz): *δ* 8.11 (d, *J* = 6.4 Hz, 1H, pyrimidine-H), 7.78 (d, *J* = 8.8 Hz, 2H, -C_6_H_4_), 7.03 (d, *J* = 8.8 Hz, 2H, -C_6_H_4_), 6.87 (s, 1H, isoxazole-H), 6.68 (d, *J* = 6.4 Hz, 1H, pyrimidine-H), 4.53 (s, 2H, -CH_2_-), 3.91 (s, 4H, piperidone-H), 3.81 (s, 3H, -OCH_3_), 2.49–2.36 (m, 4H, piperidone-H); ^13^C NMR (DMSO, 101 MHz): *δ* 207.8 (-CO), 171.2, 168.3, 162.0, 161.1, 160.9, 156.5, 128.5, 121.4, 114.9, 100.7, 100.3 (Ar-C), 55.7 (-OCH_3_), 42.2 (piperidone-C), 25.4 (-CH_2_-).

### 3.29. 1-(2-(((3-(3,4-dimethoxyphenyl) isoxazol-5-yl)methyl)thio) pyrimidin-4-yl) piperidin-4-one *(**6k**)*

Yellow solid; 44% yield; mp: 104–106 °C; ^1^H NMR (DMSO, 400 MHz): *δ* 8.11 (d, *J* = 6.0 Hz, 1H, pyrimidine-H), 7.39 (d, *J* = 8.4 Hz, 2H, -C_6_H_3_), 7.04 (d, *J* = 8.4 Hz, 1H, -C_6_H_3_), 6.92 (s, 1H, isoxazole-H), 6.67 (d, *J* = 6.4 Hz, 1H, pyrimidine-H), 4.53 (s, 2H, -CH_2_-), 3.91 (s, 4H, piperidone-H), 3.82 (s, 3H, -OCH_3_), 3.80 (s, 3H, -OCH_3_), 2.43 (t, *J* = 6.0 Hz, 4H, piperidone-H); ^13^C NMR (DMSO, 101 MHz): *δ* 207.8 (-CO), 171.2, 168.4, 162.3, 160.9, 156.5, 150.8, 149.5, 121.5, 120.0, 112.3, 110.0, 100.9, 100.3 (Ar-C), 56.1, 56.0 (-OCH_3_), 42.2 (piperidone-C), 25.5 (-CH_2_-); LCMS (ESI): *m*/*z* calcd. for C_21_H_22_N_4_O_4_S: 426.4888, found = 427.2091 [M + H]^+^.

### 3.30. 1-(2-(((3-(p-tolyl) isoxazol-5-yl)methyl)thio) pyrimidin-4-yl) piperidin-4-one *(**6l**)*

Light yellow solid; 56% yield; mp: 122–124 °C; ^1^H NMR (DMSO, 400 MHz): *δ* 8.11 (d, *J* = 6.0 Hz, 1H, pyrimidine-H), 7.73 (d, *J* = 8.0 Hz, 2H, -C_6_H_4_), 7.30 (d, *J* = 8.0 Hz, 2H, -C_6_H_4_), 6.89 (s, 1H, isoxazole-H), 6.68 (d, *J* = 6.0 Hz, 1H, pyrimidine-H), 4.54 (s, 2H, -CH_2_-), 3.91 (s, 4H, piperidone-H), 2.43 (t, *J* = 6.0 Hz, 4H, piperidone-H), 2.35 (s, 3H, -CH_3_); ^13^C NMR (DMSO, 101 MHz): *δ* 207.8 (-CO), 171.4, 168.3, 162.3, 160.9, 156.5, 140.4, 130.1, 126.9, 126.2, 100.9, 100.3 (Ar-C), 42.2 (piperidone-C), 25.4 (-CH_2_-), 21.4 (-CH_3_).

### 3.31. 2-(((1-(4-Methoxyphenyl)-1H-1,2,3-triazol-4-yl) methyl)thio)-4-(piperazin-1-yl) pyrimidine *(**7a**)*

Thick brown mass; 70% yield; ^1^H NMR (CDCl_3_, 500 MHz): *δ* 8.00 (d, *J* = 6.0 Hz, 1H, pyrimidine-H), 7.84 (s, 1H, triazole-H), 7.60–7.49 (m, 2H, -C_6_H_4_), 7.02–6.90 (m, 2H, -C_6_H_4_), 6.16 (d, *J* = 6.5 Hz, 1H, pyrimidine-H), 4.45 (s, 2H, -CH_2_-), 3.82 (s, 3H, -OCH_3_), 3.59 (s, 4H, piperazine-H), 2.95–2.83 (m, 4H, piperazine-H); ^13^C NMR (CDCl_3_, 126 MHz): *δ* 169.7, 161.1, 159.8, 155.9, 146.7, 130.6, 122.2, 120.7, 114.8, 98.9 (Ar-C), 55.7 (-OCH_3_), 45.5, 44.6 (piperazine-C), 25.7 (-CH_2_-).

### 3.32. 2-(((1-(3,4-Dichlorophenyl)-1H-1,2,3-triazol-4-yl)methyl)thio)-4-(piperazin-1-yl)pyrimidine *(**7b**)*

Thick brown mass; 50% yield; ^1^H NMR (CDCl_3_, 500 MHz): *δ* 7.79 (d, *J* = 6.0 Hz, 1H, pyrimidine-H), 7.71 (s, 1H, triazole-H), 7.67–7.57 (m, 1H, -C_6_H_3_), 7.33 (d, *J* = 1.0 Hz, 2H, -C_6_H_3_), 5.96 (d, *J* = 6.0 Hz, 1H, pyrimidine-H), 4.24 (s, 2H, -CH_2_-), 3.37 (s, 4H, piperazine-H), 2.73–2.61 (m, 4H, piperazine-H); ^13^C NMR (CDCl_3_, 126 MHz): *δ* 169.3, 161.0, 155.7, 147.5, 135.9, 133.8, 132.5, 131.3, 122.0, 120.1, 119.2, 98.8 (Ar-C), 45.5, 44.6 (piperazine-C), 25.4 (-CH_2_-).

### 3.33. 4-(4-(((4-(Piperazin-1-yl)pyrimidin-2-yl)thio)methyl)-1H-1,2,3-triazo1-1-yl)phenol *(**7c**)*

Yellow solid; 58% yield; mp: 204–206 °C; ^1^H NMR (DMSO, 500 MHz): *δ* 8.41 (s, 1H, triazole-H), 7.97 (d, *J* = 6.0 Hz, 1H, pyrimidine-H), 7.56 (d, *J* = 8.5 Hz, 2H, -C_6_H_4_), 6.87 (d, *J* = 9.0 Hz, 2H, -C_6_H_4_), 6.48 (d, *J* = 6.0 Hz, 1H, pyrimidine-H), 4.35 (s, 2H, -CH_2_-), 3.47 (s, 4H, piperazine-H), 2.65 (s, 4H, piperazine-H); ^13^C NMR (DMSO, 126 MHz): *δ* 169.1, 161.2, 158.8, 156.1, 145.4, 128.9, 122.3, 121.7, 116.6, 99.9 (Ar-C), 45.8, 45.2 (piperazine-C), 25.3 (-CH_2_-).

### 3.34. 3-(4-Methoxyphenyl)-5-(((4-(piperazin-1-yl)pyrimidin-2-yl)thio)methyl)isoxazole *(**7d**)*

Yellow solid; 60% yield; mp: 88–90 °C; ^1^H NMR (CDCl_3_, 500 MHz): *δ* 8.02 (d, *J* = 4.5 Hz, 1H, pyrimidine-H), 7.70 (d, *J* = 7.5 Hz, 2H, -C_6_H_4_), 6.94 (d, *J* = 7.5 Hz, 2H, -C_6_H_4_), 6.49 (s, 1H, isoxazole-H), 6.20 (d, *J* = 4.5 Hz, 1H, pyrimidine-H), 4.40 (s, 2H, -CH_2_-), 3.84 (s, 3H, -OCH_3_), 3.60 (s, 4H, piperazine-H), 2.91 (s, 4H, piperazine-H); ^13^C NMR (CDCl_3_, 126 MHz): *δ* 172.9, 170.7, 164.2, 163.1, 162.9, 157.9, 130.2, 123.6, 116.3, 102.2, 101.0 (Ar-C), 57.4 (-OCH_3_), 47.6, 46.8 (piperazine-C), 27.8 (-CH_2_-); LCMS (ESI): *m*/*z* calcd. for C_19_H_21_N_5_O_2_S: 383.4673, found = 384.2190 [M + H]^+^.

### 3.35. 3-(3,4-Dimethoxyphenyl)-5-(((4-(piperazin-1-yl)pyrimidin-2-yl)thio)methyl)isoxazole *(**7e**)*

Thick brown mass; 54% yield; ^1^H NMR (CDCl_3_, 500 MHz): *δ* 8.02 (d, *J* = 6.5 Hz, 1H, pyrimidine-H), 7.37 (d, *J* = 2.0 Hz, 1H, -C_6_H_3_), 7.25 (dd, *J* = 8.0, 2.0 Hz, 1H, -C_6_H_3_), 6.90 (d, *J* = 8.0 Hz, 1H, -C_6_H_3_), 6.51 (s, 1H, isoxazole-H), 6.20 (d, *J* = 6.5 Hz, 1H, pyrimidine-H), 4.41 (s, 2H, -CH_2_-), 3.94 (s, 3H, -OCH_3_), 3.92 (s, 3H, -OCH_3_), 3.59 (s, 4H, piperazine-H), 2.90 (t, *J* = 5.0 Hz, 4H, piperazine-H); ^13^C NMR (CDCl_3_, 126 MHz): *δ* 171.0, 168.7, 162.3, 161.2, 155.9, 150.6, 149.3, 121.9, 119.9, 111.1, 109.3, 100.3, 99.1 (Ar-C), 56.1, 56.0 (-OCH_3_), 45.7, 44.9 (piperazine-C), 25.9 (-CH_2_-).

### 3.36. 5-(((4-(Piperazin-1-yl)pyrimidin-2-yl)thio)methyl)-3-(p-tolyl)isoxazole *(**7f**)*

Thick light brown mass; 64% yield; ^1^H NMR (CDCl_3_, 500 MHz): *δ* 8.02 (d, *J* = 6.5 Hz, 1H, pyrimidine-H), 7.65 (d, *J* = 8.0 Hz, 2H, -C_6_H_4_), 7.23 (d, *J* = 8.0 Hz, 2H, -C_6_H_4_), 6.52 (s, 1H, isoxazole-H), 6.19 (d, *J* = 6.0 Hz, 1H, pyrimidine-H), 4.40 (s, 2H, -CH_2_-), 3.61 (s, 4H, piperazine-H), 2.92 (s, 4H, piperazine-H), 2.38 (s, 3H, -CH_3_); ^13^C NMR (CDCl_3_, 126 MHz): *δ* 171.0, 168.8, 162.5, 161.1, 156.0, 140.1, 129.6, 126.7, 126.3, 100.4, 99.1 (Ar-C), 45.6, 44.7 (piperazine-C), 25.9 (-CH_2_-), 21.5 (-CH_3_).

### 3.37. 1-(2-(((1-(4-Methoxyphenyl)-1H-1,2,3-triazol-4-yl)methyl)thio)pyrimidin-4-yl)piperidin-4-ol *(**8a**)*

Thick off-white mass; 60% yield; ^1^H NMR (CDCl_3_, 500 MHz): *δ* 7.99 (d, *J* = 6.5 Hz, 1H, pyrimidine-H), 7.85 (s, 1H, triazole-H), 7.56 (d, *J* = 9.0 Hz, 2H, -C_6_H_4_), 6.97 (d, *J* = 9.0 Hz, 2H, -C_6_H_4_), 6.21 (d, *J* = 6.5 Hz, 1H, pyrimidine-H), 4.47 (s, 2H, -CH_2_-), 4.06–3.88 (m, 4H, piperidine-H (3H) and OH (1H)), 3.83 (s, 3H, -OCH_3_), 3.35–3.20 (m, 2H, piperidine-H), 1.96–1.82 (m, 2H, piperidine-H), 1.58–1.41 (m, 2H, piperidine-H); ^13^C NMR (CDCl_3_, 126 MHz): *δ* 169.7, 160.8, 159.8, 155.9, 146.7, 130.6, 122.2, 120.7, 114.8, 98.9 (Ar-C), 67.3 (piperidine-C (quaternary)), 55.7 (-OCH_3_), 41.3, 33.7 (piperidine-C), 25.8 (-CH_2_-).

### 3.38. 1-(2-(((1-(3,4-Dichlorophenyl)-1H-1,2,3-triazol-4-yl)methyl)thio)pyrimidin-4-yl)piperidin-4-ol *(**8b**)*

Thick pink mass; 55% yield; ^1^H NMR (CDCl_3_, 500 MHz): *δ* 7.97 (d, *J* = 6.5 Hz, 1H, pyrimidine-H), 7.94 (s, 1H, triazole-H), 7.83 (t, *J* = 1.5 Hz, 1H, -C_6_H_3_), 7.54 (d, *J* = 1.5 Hz, 2H, -C_6_H_3_), 6.21 (d, *J* = 6.5 Hz, 1H, pyrimidine-H), 4.45 (s, 2H, -CH_2_-), 4.16–3.88 (m, 4H, piperidine-H (3H) and OH (1H)), 3.34–3.19 (m, 2H, piperidine-H), 1.95–1.81 (m, 2H, piperidine-H), 1.58–1.41 (m, 2H, piperidine-H); ^13^C NMR (CDCl_3_, 126 MHz): *δ* 169.4, 160.8, 155.8, 147.7, 136.1, 134.0, 132.8, 131.5, 122.2, 120.4, 119.4, 99.0 (Ar-C), 67.1 (piperidine-C (quaternary)), 41.4, 33.7 (piperidine-C), 25.6 (-CH_2_-); LCMS (ESI): *m*/*z* calcd. for C_18_H_18_Cl_2_N_6_OS: 437.3461, found = 437.1480 [M + H]^+^, 439.1444 [M + 2H]^+^.

### 3.39. 1-(2-(((1-(4-Hydroxyphenyl)-1H-1,2,3-triazol-4-yl)methyl)thio) pyrimidin-4-yl) piperidin-4-ol *(**8c**)*

Yellow solid; 58% yield; mp: 104–106 °C; ^1^H NMR (DMSO, 400 MHz): *δ* 9.94 (s, 1H, -OH-C_6_H_4_), 8.47 (s, 1H, triazole-H), 8.01 (d, *J* = 6.0 Hz, 1H, pyrimidine-H), 7.63 (d, *J* = 8.8 Hz, 2H, -C_6_H_4_), 6.91 (d, *J* = 8.4 Hz, 2H, -C_6_H_4_), 6.57 (d, *J* = 6.0 Hz, 1H, pyrimidine-H), 4.77 (d, *J* = 3.2 Hz, 1H, piperidine-H), 4.41 (s, 2H, -CH_2_-), 4.01 (d, *J* = 6.8 Hz, 2H, piperidine-H (1H) and OH (1H)), 3.74 (d, *J* = 3.2 Hz, 1H, piperidine-H), 3.31–3.16 (m, 2H, piperidine-H), 1.75 (d, *J* = 9.2 Hz, 2H, piperidine-H), 1.31 (d, *J* = 9.2 Hz, 2H, piperidine-H); ^13^C NMR (DMSO, 101 MHz): *δ* 169.1, 160.9, 158.1, 156.1, 145.4, 129.2, 122.3, 121.6, 116.5, 99.9 (Ar-C), 66.0 (piperidine-C (quaternary)), 41.6, 34.1 (piperidine-C), 25.3 (-CH_2_-); LCMS (ESI): *m*/*z* calcd. for C_18_H_20_N_6_O_2_S: 384.4554, found = 385.1543 [M + H]^+^.

### 3.40. 1-(2-(((3-(4-Methoxyphenyl) isoxazol-5-yl)methyl)thio) pyrimidin-4-yl) piperidin-4-ol *(**8d**)*

Yellow solid; 64% yield; mp: 98–100 °C; ^1^H NMR (CDCl_3_, 500 MHz): *δ* 7.98 (d, *J* = 6.5 Hz, 1H, pyrimidine-H), 7.67 (d, *J* = 9.0 Hz, 2H, -C_6_H_4_), 6.92 (d, *J* = 8.5 Hz, 2H, -C_6_H_4_), 6.47 (s, 1H isoxazole-H), 6.21 (d, *J* = 6.0 Hz, 1H, pyrimidine-H), 4.38 (s, 2H, -CH_2_-), 4.02–3.86 (m, 4H piperidine-H (3H) and OH (1H)), 3.82 (s, 3H, -OCH_3_), 3.35–3.20 (m, 2H, piperidine-H), 1.96–1.81 (m, 2H, piperidine-H), 1.58–1.41 (m, 2H, piperidine-H); ^13^C NMR (CDCl_3_, 126 MHz): *δ* 171.0, 168.8, 162.2, 161.0, 160.8, 155.9, 128.2, 121.7, 114.3, 100.3, 99.1 (Ar-C), 67.2 (piperidine-C (quaternary)), 55.4 (-OCH_3_), 41.3, 33.7 (piperidine-C), 25.9 (-CH_2_-); LCMS (ESI): *m*/*z* calcd. for C_20_H_22_N_4_O_3_S: 398.4787, found = 399.1363 [M + H]^+^.

### 3.41. 1-(2-(((3-(3,4-Dimethoxyphenyl) isoxazol-5-yl)methyl)thio) pyrimidin-4-yl) piperidin-4-ol *(**8e**)*

Thick off-white mass; 58% yield; ^1^H NMR (CDCl_3_, 500 MHz): *δ* 8.00 (d, *J* = 6.5 Hz, 1H pyrimidine-H), 7.37 (d, *J* = 1.5 Hz, 1H, -C_6_H_3_), 7.25 (dd, *J* = 8.5, 1.5 Hz, 1H, -C_6_H_3_), 6.90 (d, *J* = 8.5 Hz, 1H, -C_6_H_3_), 6.52 (s, 1H, isoxazole-H), 6.24 (d, *J* = 6.5 Hz, 1H, pyrimidine-H), 4.41 (s, 2H, -CH_2_-), 4.07–3.91 (m, 4H, piperidine-H (3H) and OH (1H)), 3.94 (s, 3H, -OCH_3_), 3.92 (s, 3H, -OCH_3_), 3.37–3.23 (m, 2H, piperidine-H), 1.99–1.85 (m, 2H, piperidine-H), 1.61–1.44 (m, 2H, piperidine-H); ^13^C NMR (CDCl_3_, 126 MHz): *δ* 171.1, 168.8, 162.3, 160.8, 155.9, 150.6, 149.3, 121.9, 119.9, 111.1, 109.3, 100.3, 99.1 (Ar-C), 67.2 (piperidine-C (quaternary)), 56.1, 56.0 (-OCH_3_), 41.3, 33.7 (piperidine-C), 25.9 (-CH_2_-).

### 3.42. 1-(2-(((3-(p-tolyl)isoxazol-5-yl)methyl)thio)pyrimidin-4-yl) piperidin-4-ol *(**8f**)*

Thick yellow mass; 66% yield; ^1^H NMR (CDCl_3_, 500 MHz): *δ* 7.98 (d, *J* = 6.0 Hz, 1H, pyrimidine-H), 7.64 (d, *J* = 8.0 Hz, 2H, -C_6_H_4_), 7.23 (d, *J* = 8.0 Hz, 2H, -C_6_H_4_), 6.52 (s, 1H, isoxazole-H), 6.22 (d, *J* = 6.5 Hz, 1H, pyrimidine-H), 4.40 (s, 2H, -CH_2_-), 4.05–3.89 (m, 4H, piperidine-H (3H) and OH (1H)), 3.36–3.22 (m, 2H, piperidine-H), 2.37 (s, 3H, -CH_3_), 1.97–1.83 (m, 2H, piperidine-H), 1.60–1.43 (m, 2H, piperidine-H); ^13^C NMR (CDCl_3_, 126 MHz): *δ* 171.0, 168.7, 162.6, 160.8, 155.9, 140.1, 129.6, 126.7, 126.3, 100.4, 99.1 (Ar-C), 67.2 (piperidine-C (quaternary)), 41.3, 33.7 (piperidine-C), 25.9 (-CH_2_-), 21.5 (-CH_3_); LCMS (ESI): *m*/*z* calcd. for C_20_H_22_N_4_O_2_S: 382.4793, found = 383.1446 [M + H]^+^.

### 3.43. Cell Viability Assay

First, 2 × 10^3^ MCF-7 cells were cultured in MEM, or Leibovitz’s L-15 medium enriched with 2% FBS, and maintained in a humidified atmosphere of 5% CO_2_ at 37 °C [44]. DMSO-dissolved compounds were kept as a stock solution and diluted with a cell culture medium to the desired concentration. Cancer cells (4 × 10^3^) were grown overnight in 96-well plates, cultured, and treated with triazoles/isoxazoles at 0, 0.01, 0.1, 10, 100, and 1000 µM concentrations for 72 h. The inhibitory effect of the compounds was assessed using the Alamar Blue reagent.

### 3.44. Molecular Docking Studies

The Scripps Research Institute’s AutoDock4 tools (ADT) v1.5.6 [45] were used to determine the docking studies. The co-crystal structure of HDAC7 (PDB ID: 3ZNR) was obtained from the Protein Data Bank (PDB), and the structure was downloaded in PDB format for further analysis. Then, ligand structures, including the novel isoxazole (**7d**), triazole (**5h**) analogues, and the co-crystal ligand, were prepared. The ligand structures were built and optimized using molecular modeling software (Discovery Studio & Avogadro) [46] to ensure proper geometry and energy minimization [47,48,49,50,51,52,53]. The ligand structures were saved in the appropriate file format (PDB) for AutoDock4 Tools. Later, the HDAC7 crystal structure (**PDB ID: 3ZNR**) was loaded into AutoDock4 Tools, and water molecules, co-factors, and other non-essential entities were removed to isolate the protein of interest. The protein structure was prepared by adding polar hydrogen atoms and assigning Kollman charges. A grid box was defined around the HDAC7 to guide the docking simulation with dimensions 60Å × 60 Å × 70 Å with a spacing of 1Å. Then, docking was initiated by selecting macromolecule (HDAC7) and ligand (**7d**) with a genetic algorithm as the search parameter was set, and the output file was the Lamarckian default parameter file. Initially, 150 were randomly placed individually with a maximum number of 2.5 × 10^6^ energy evaluations having a mutation rate of 0.02 and crossover rate of 0.80, and 10 docking runs were performed for compound **7d**. Similarly, all steps were performed for the compound **5h** and co-crystal ligand (TMP269). Visualization of docking results was examined using Discovery Studio [54], pymol [55], and UCSF chimera 1.16 [56].

## 4. Conclusions

In conclusion, the novel thiouracil-tethered triazole-based compounds were synthesized by both conventional and electrolytic methods. The synthesis of 1,2,3-triazoles via electrochemical oxidation provided a good yield and was found to be highly useful for synthesizing bioactive molecules based on triazoles. Moreover, the thiouracil-tethered isoxazole-based compounds were synthesized and presented a better procedure to prepare at a high yield. Additionally, we offered a unique green protocol for synthesizing isoxazole- and triazole-based compounds in the presence of the thiouracil group. Herein, in a target-based study, we further examined the discovered compound’s mode of action in silico by considering the reference co-crystal structure of HDAC and its inhibitors and showed that the newly synthesized compounds could mimic HDACis in BC cells. The lead compound, such as 6l or 5h, could effectively inhibit BC cell proliferation with a lower IC_50_ value. Furthermore, we discovered through an in silico investigation that these lead compounds could bind to the catalytic areas of HDAC. In conclusion, we provided a new chemical entity bearing triazole, isoxazole, and thiouracil moiety, which might target HDAC in BC cells.

## Figures and Tables

**Table 1:**
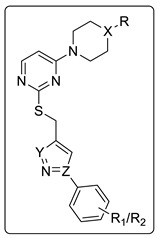
List of compounds synthesized.

Compound Code	X,Y,Z	R	R_1_/R_2_	MCF-7/IC_50_ (μM)
**5a**	N,N,N	2,3-Cl_2_C_6_H_3_	4-OCH_3_	>100
**5b**	N,N,N	2,3-Cl_2_C_6_H_3_	3,4-Cl_2_	>100
**5c**	N,N,N	2,3-Cl_2_C_6_H_3_	4-OH	11.31
**5d**	N,N,N	COOC(CH_3_)_3_	4-OCH_3_	26.59
**5e**	N,N,N	COOC(CH_3_)_3_	3,4-Cl_2_	5.85
**5f**	N,N,N	COOC(CH_3_)_3_	4-OH	24.12
**5g**	O,N,N	-	4-OCH_3_	>100
**5h**	O,N,N	-	3,4-Cl_2_	8.754
**5i**	O,N,N	-	4-OH	>100
**5j**	C,N,N	O	4-OCH_3_	16.14
**5k**	C,N,N	O	3,4-Cl_2_	30.10
**5l**	C,N,N	O	4-OH	23.20
**6a**	N,O,C	2,3-Cl_2_C_6_H_3_	4-OCH_3_	66.09
**6b**	N,O,C	2,3-Cl_2_C_6_H_3_	3,4-(OCH_3_)_2_	28.39
**6c**	N,O,C	2,3-Cl_2_C_6_H_3_	4-CH_3_	>100
**6d**	N,O,C	COOC(CH_3_)_3_	4-OCH_3_	15.96
**6e**	N,O,C	COOC(CH_3_)_3_	3,4-(OCH_3_)_2_	>100
**6f**	N,O,C	COOC(CH_3_)_3_	4-CH_3_	19.84
**6g**	O,O,C	-	4-OCH_3_	10.84
**6h**	O,O,C	-	3,4-(OCH_3_)_2_	9.867
**6i**	O,O,C	-	4-CH_3_	43.27
**6j**	C,O,C	O	4-OCH_3_	26.90
**6k**	C,O,C	O	3,4-(OCH_3_)_2_	33.35
**6l**	C,O,C	O	4-CH_3_	11.71
**7a**	N,N,N	H	4-OCH_3_	>100
**7b**	N,N,N	H	3,4-Cl_2_	14.7
**7c**	N,N,N	H	4-OH	>100
**7d**	N,O,C	H	4-OCH_3_	14.83
**7e**	N,O,C	H	3,4-(OCH_3_)_2_	>100
**7f**	N,O,C	H	4-CH_3_	28.94
**8a**	C,N,N	OH	4-OCH_3_	>100
**8b**	C,N,N	OH	3,4-Cl_2_	30.26
**8c**	C,N,N	OH	4-OH	>100
**8d**	C,O,C	OH	4-OCH_3_	21.87
**8e**	C,O,C	OH	3,4-(OCH_3_)_2_	19.38
**8f**	C,O,C	OH	4-CH_3_	15.33
Doxorubicin	0.71			

## Data Availability

All data are freely available within this article.

## Supplementary Materials

The following supporting information can be downloaded at https://www.mdpi.com/article/10.3390/molecules28135254/s1. Supplementary data contains NMR, LCMS, and IC_50_ values of newly synthesized compounds (**5a**–**l**), (**6a**–**l**), (**7a**–**f**), and (**8a**–**f**).

## Sample Availability

Samples of the compounds are not available from the authors.
