# Supplementary material for: Electrochemical Synthesis of New Isoxazoles and Triazoles Tethered with Thiouracil Base as Inhibitors of Histone Deacetylases in Human Breast Cancer Cells
Source: Molecules. 2023 Jul 6;28(13):5254. doi: 10.3390/molecules28135254 (PMC10343668; doi:10.3390/molecules28135254)
Supplement: Supplementary file 1 [file molecules-28-05254-s001.zip › molecules-2376705-supplementary.pdf]

Electrochemical Synthesis of New Isoxazoles and Triazoles Tethered with Thiouracil Base as Inhibitors of Histone Deacetylases in Human Breast Cancer Cells

Divakar Vishwanath, Zhang Xi, Akshay Ravish, ArunKumar Mohan, Shreeja Basappa, Santosh L. Gaonkar, Niranjan Pattehalli Krishnamurthy, Vijay Pandey, Peter E. Lobie, and Basappa Basappa.

Supplementary data

Spectroscopic data (Mass, ^{1}H, and ^{13}C NMR) of synthesized compounds

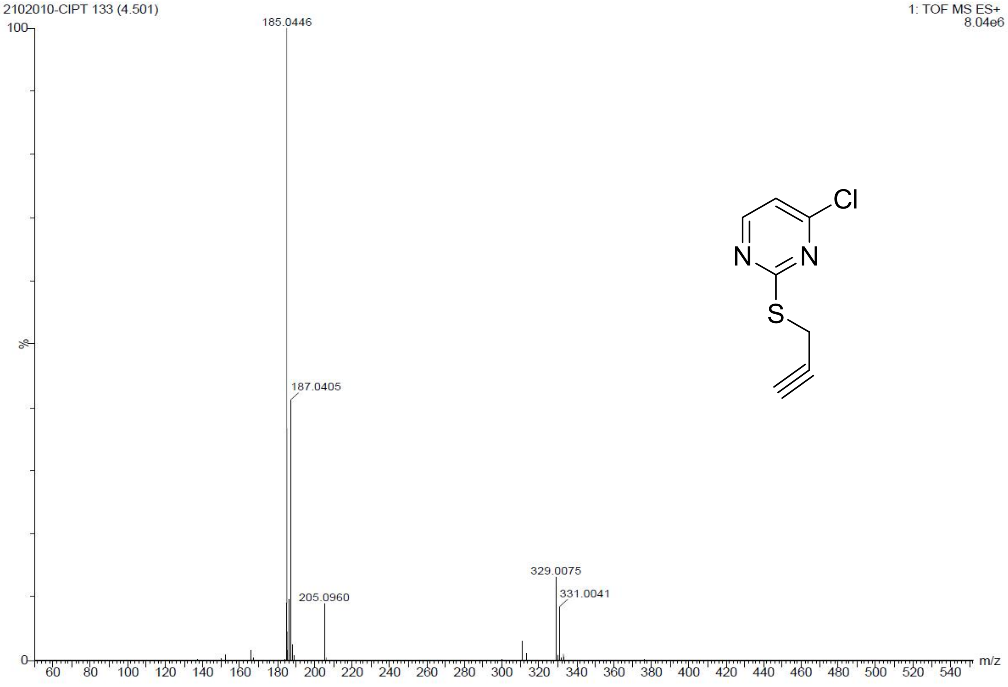

Mass spectrum of 3

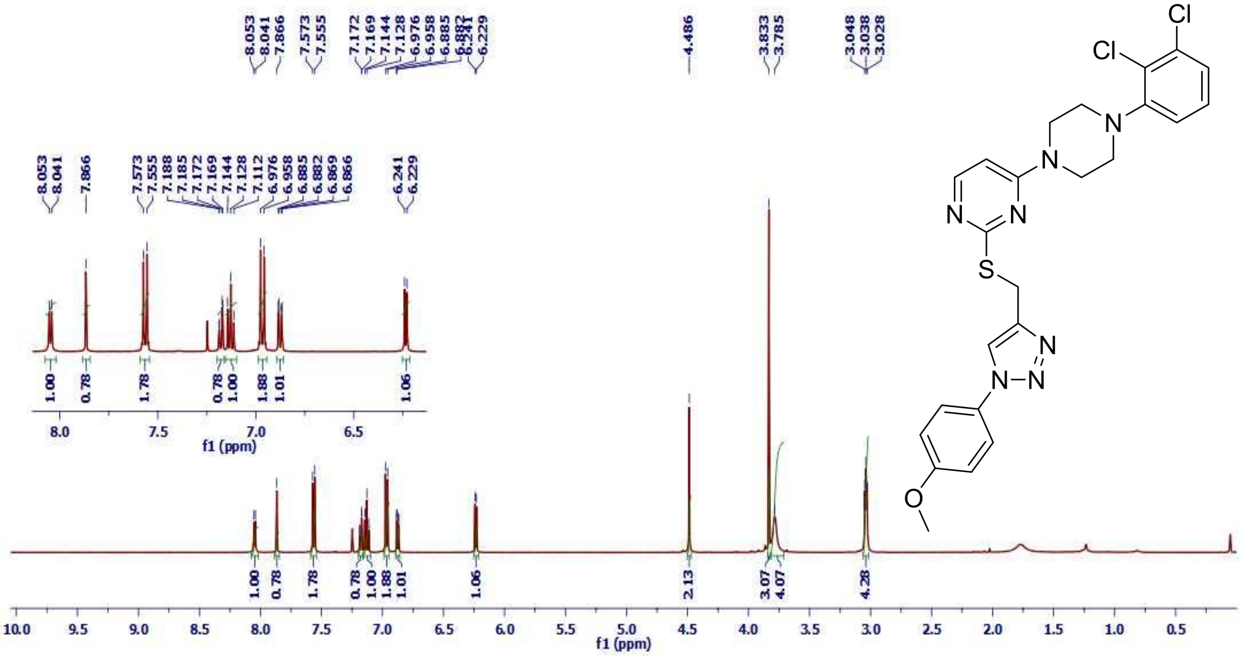

^{1}H NMR of 5a

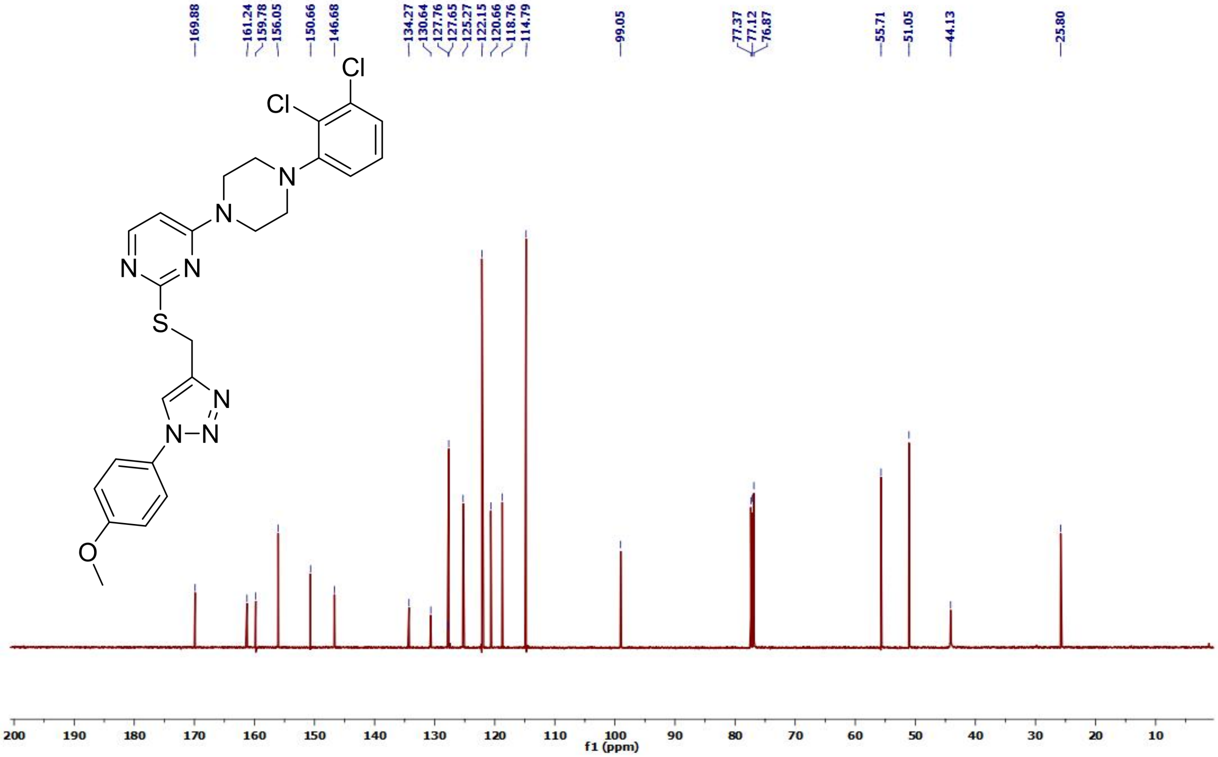

^{13}C NMR of 5a

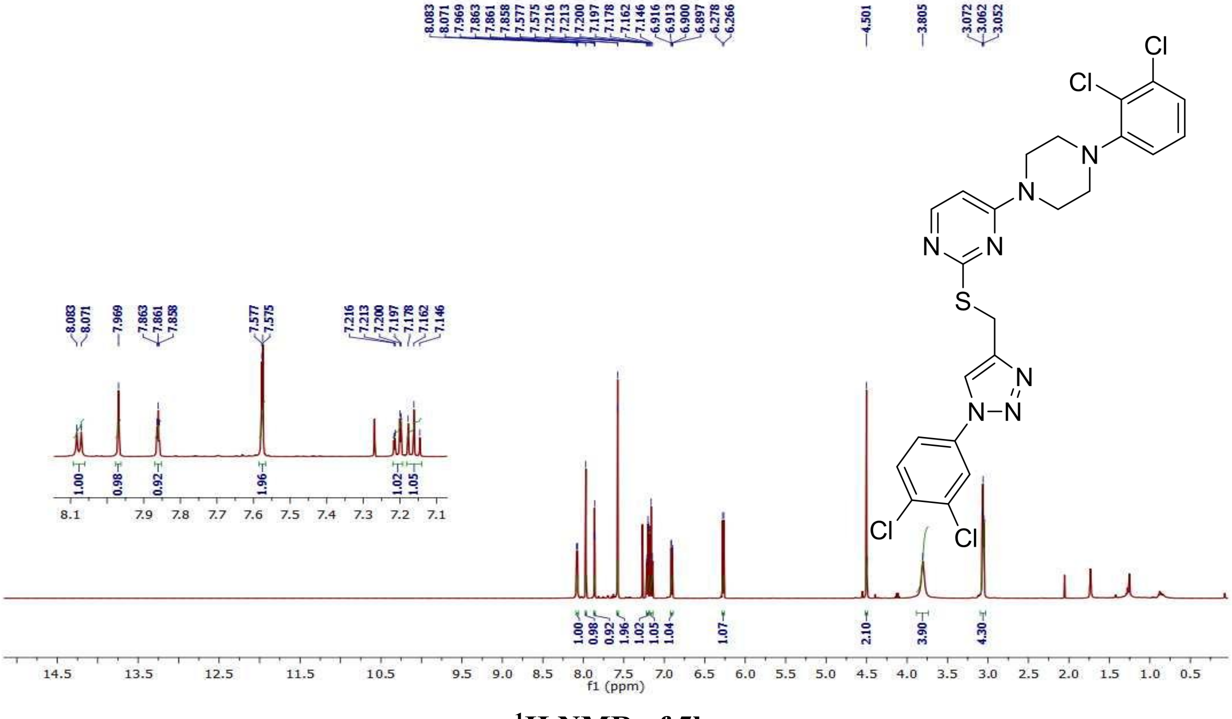

^{1}H NMR of 5b

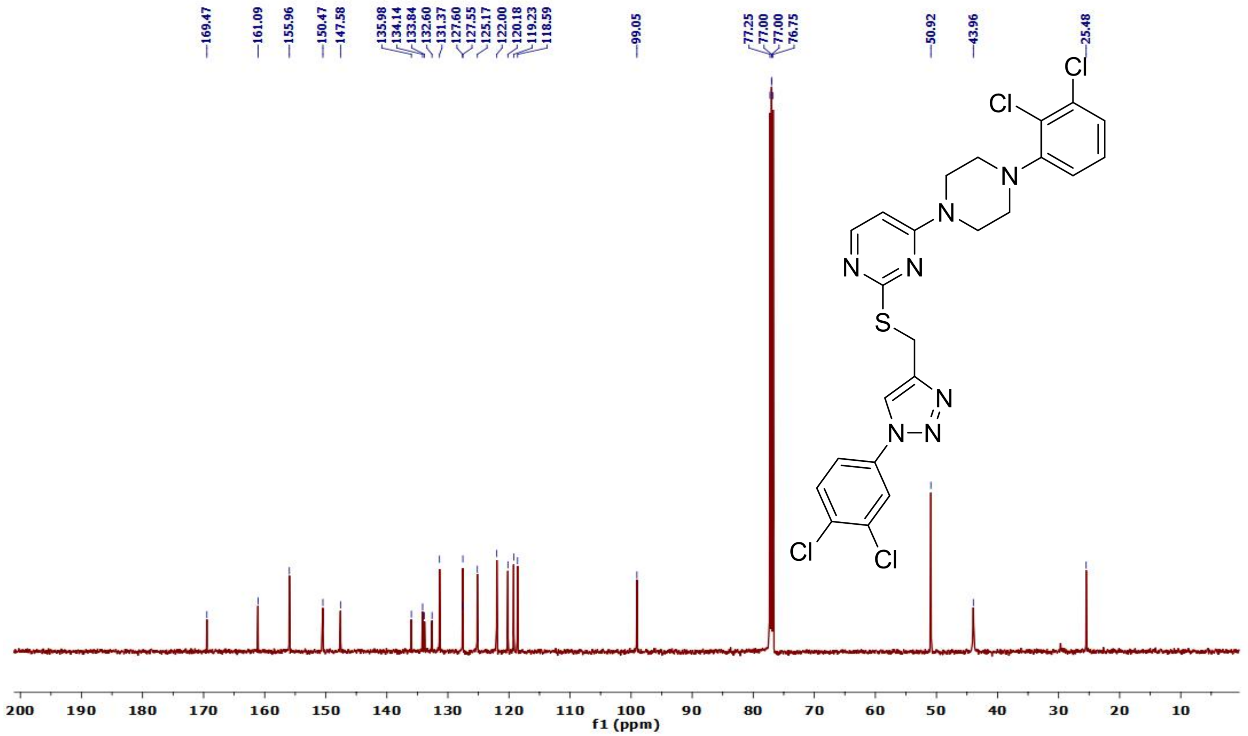

^{13}C NMR of 5b

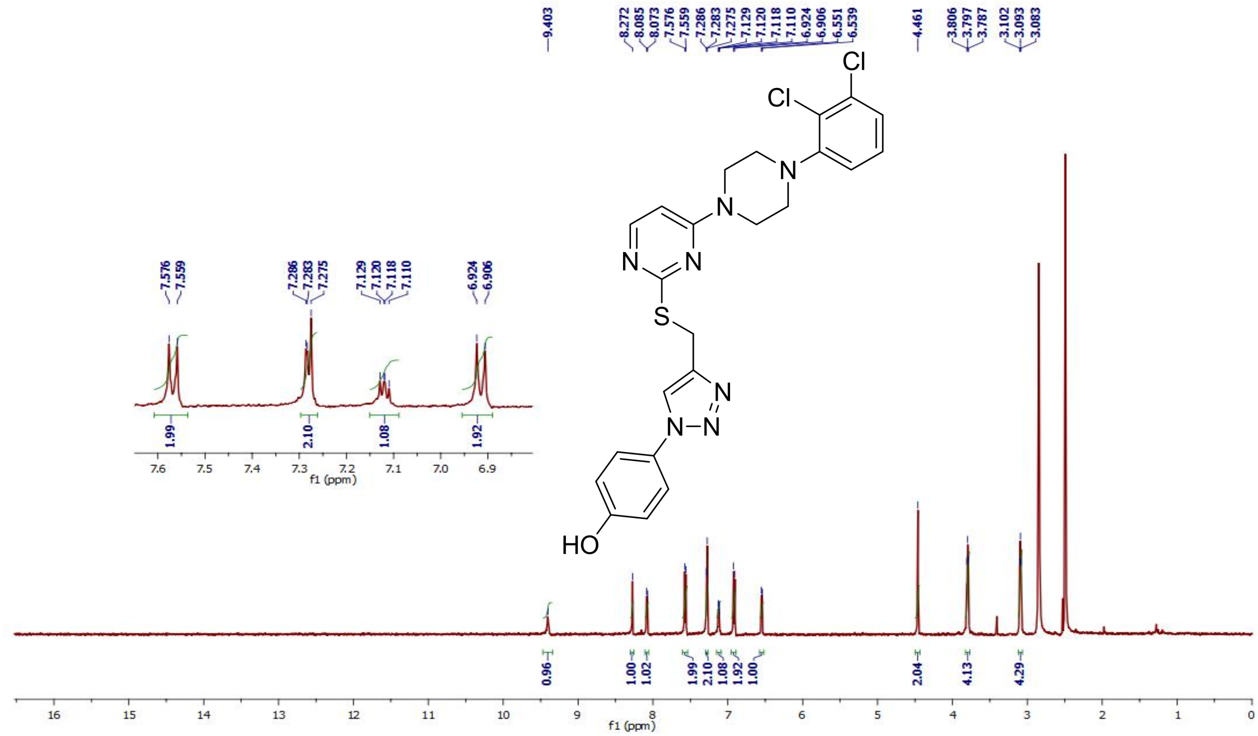

^{1}H NMR of 5c

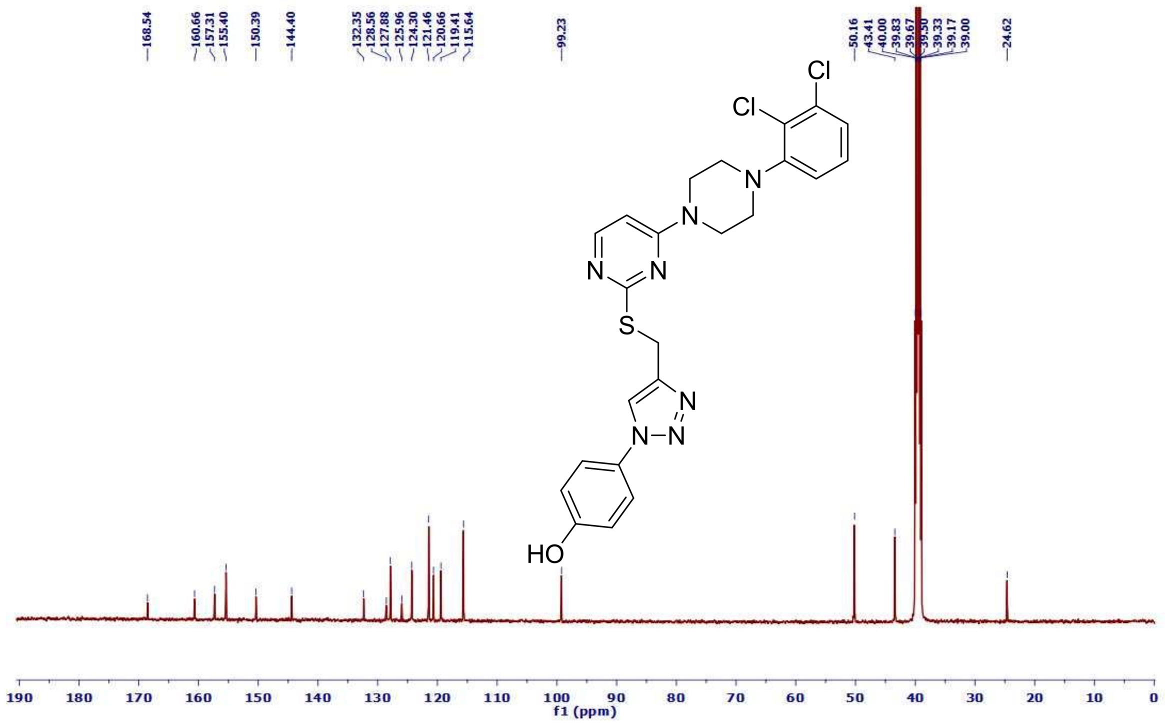

^{13}C NMR of 5c

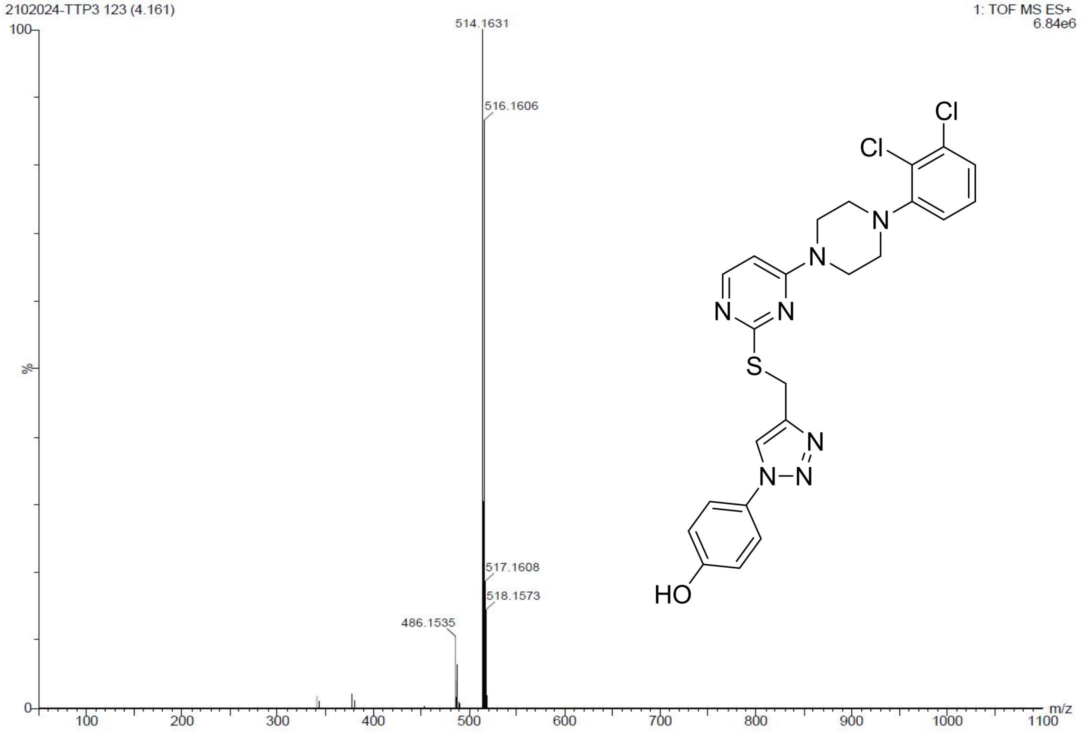

Mass spectrum of 5c

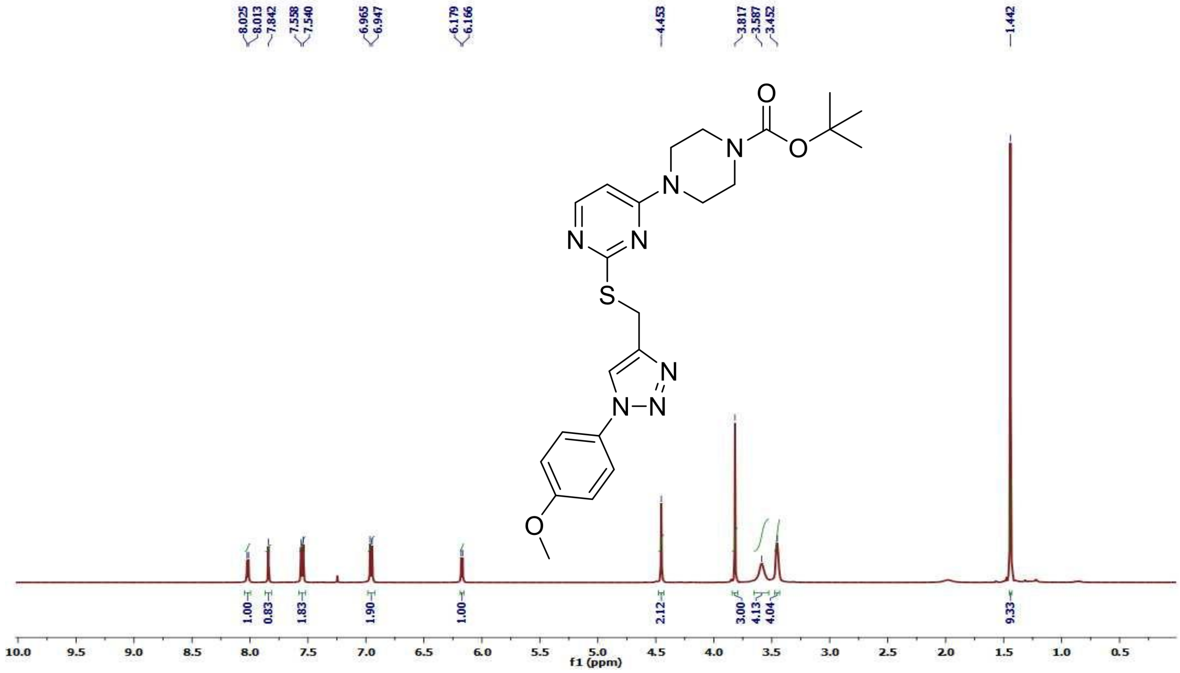

^{1}H NMR of 5d

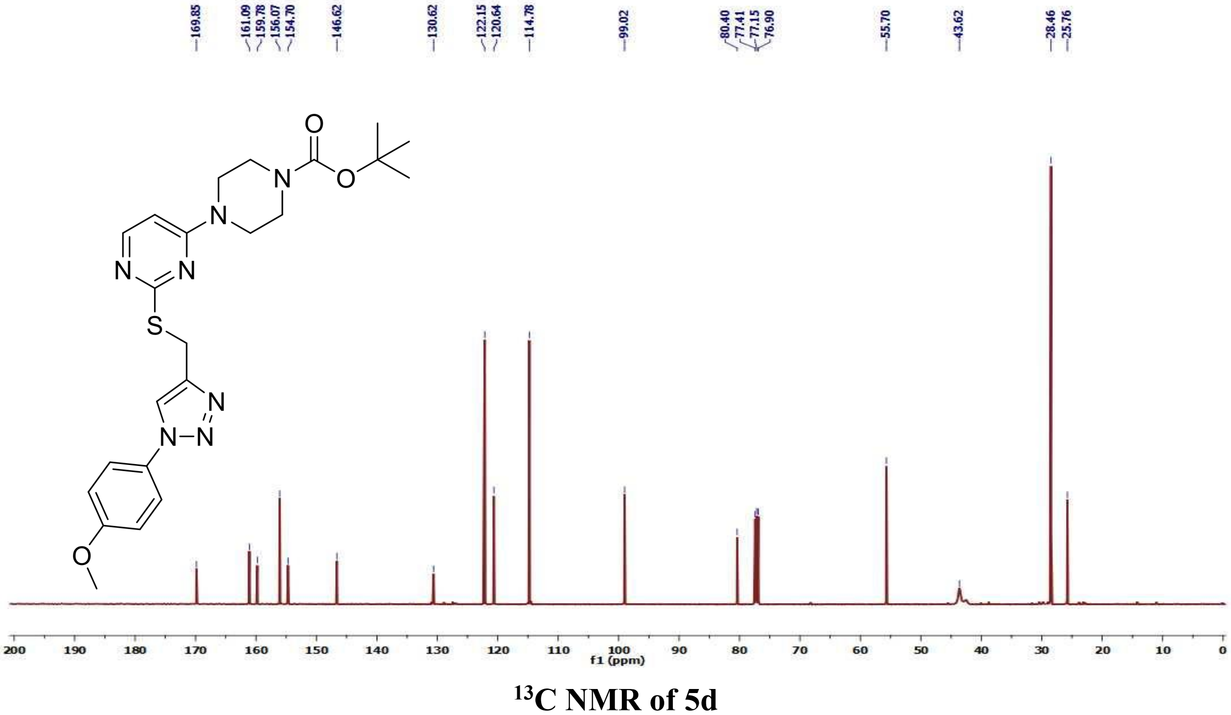

^{13}C NMR of 5d

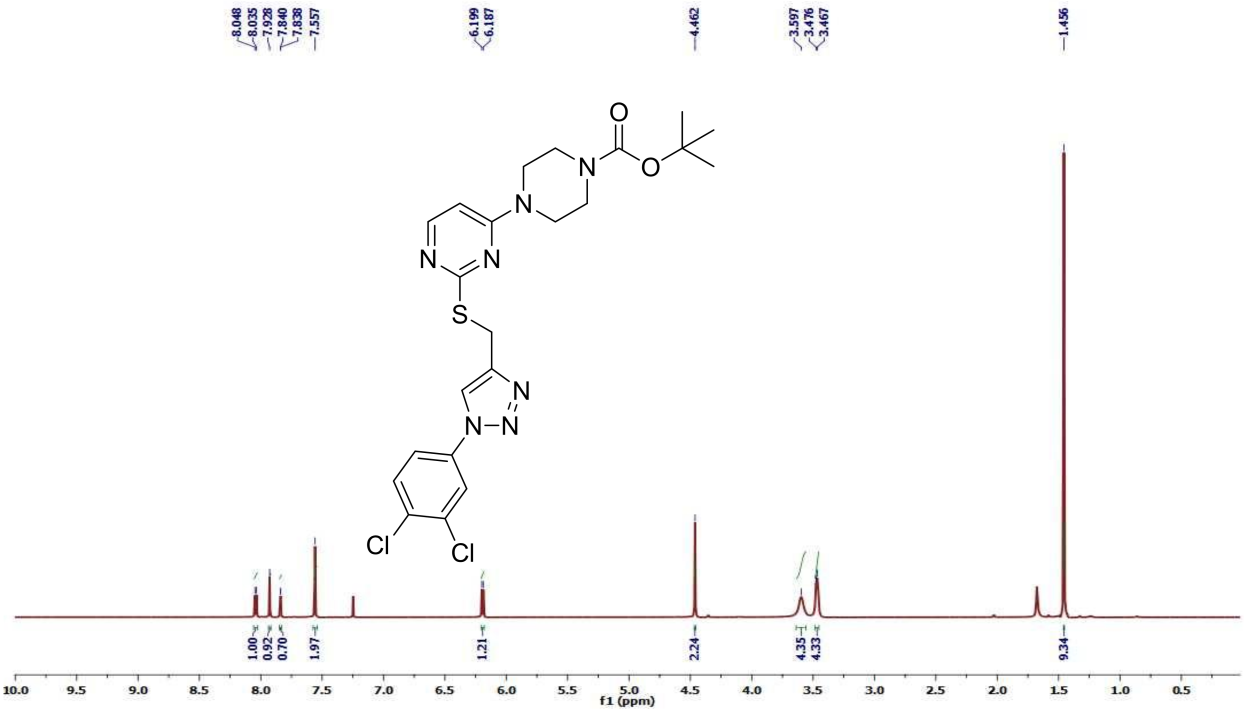

^{1}H NMR of 5e

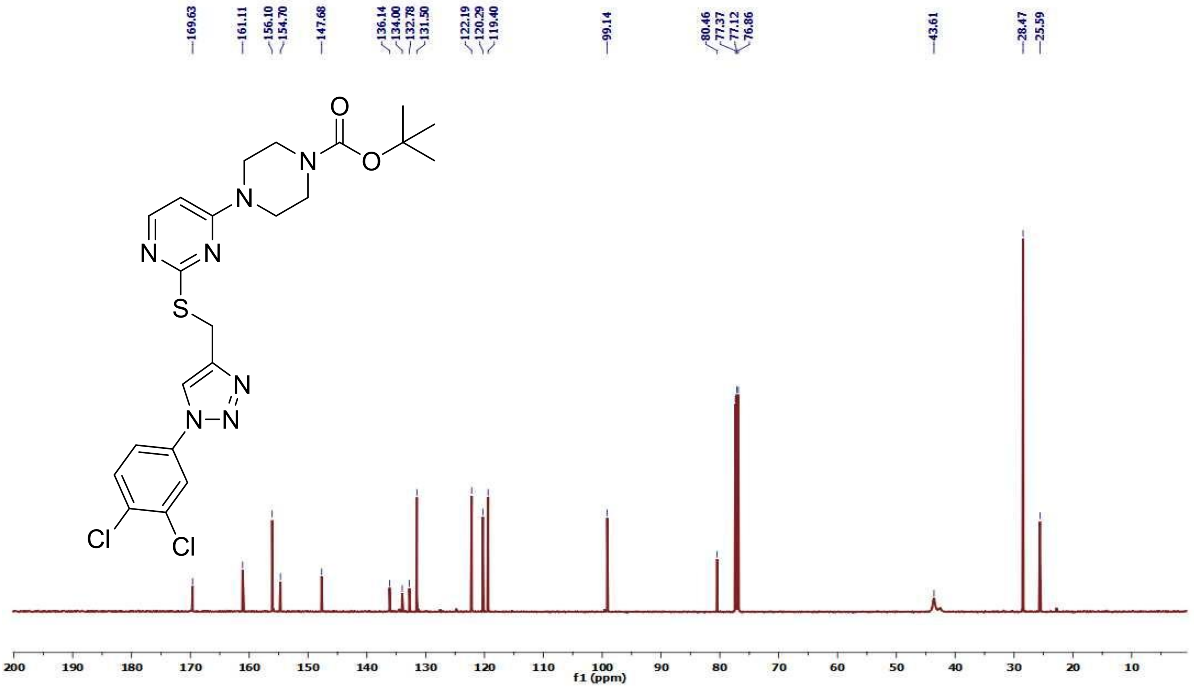

^{13}C NMR of 5e

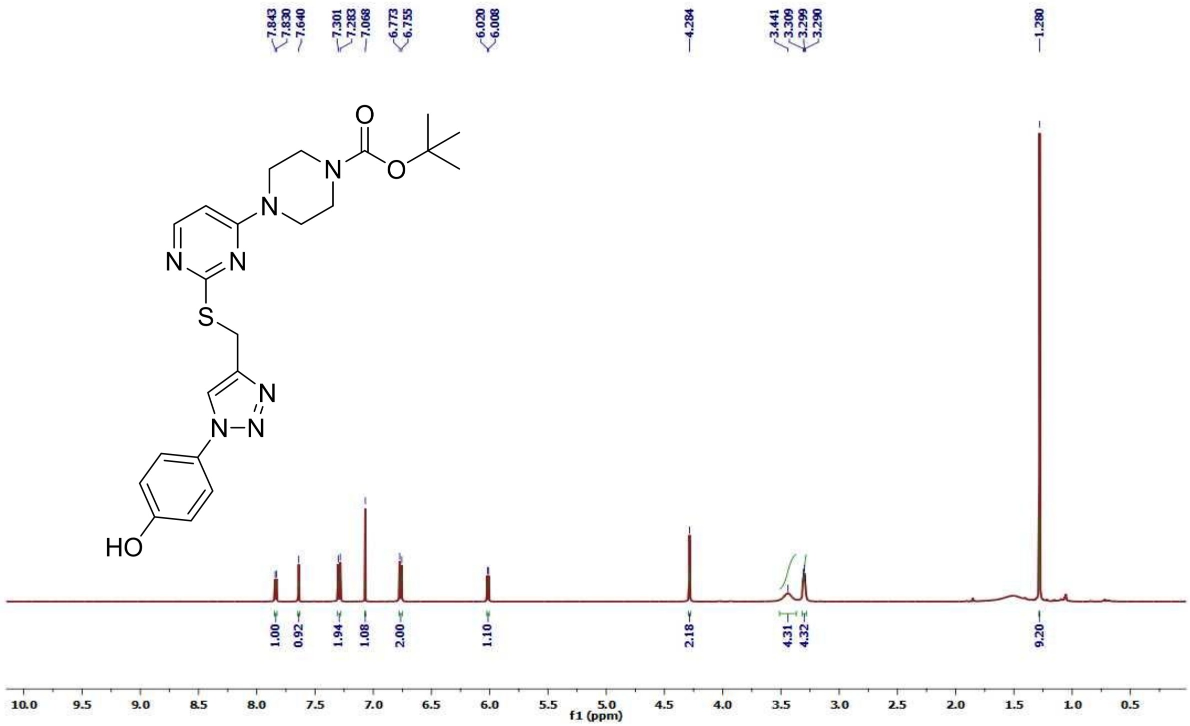

^{1}H NMR of 5f

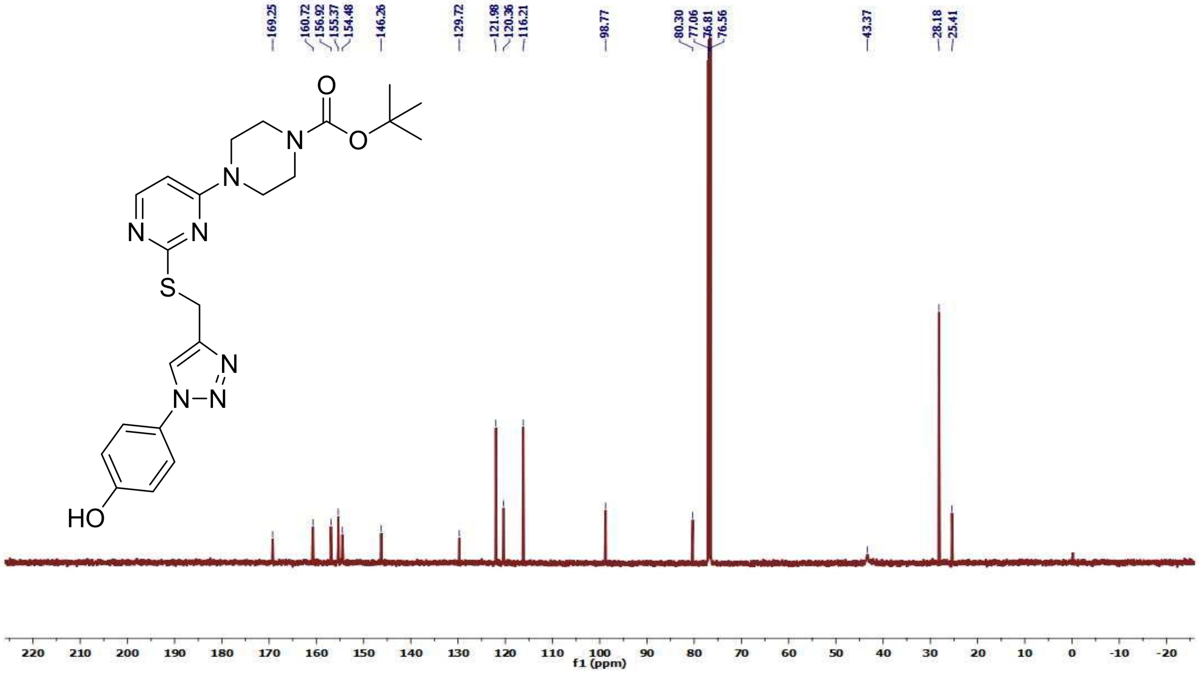

^{13}C NMR of 5f

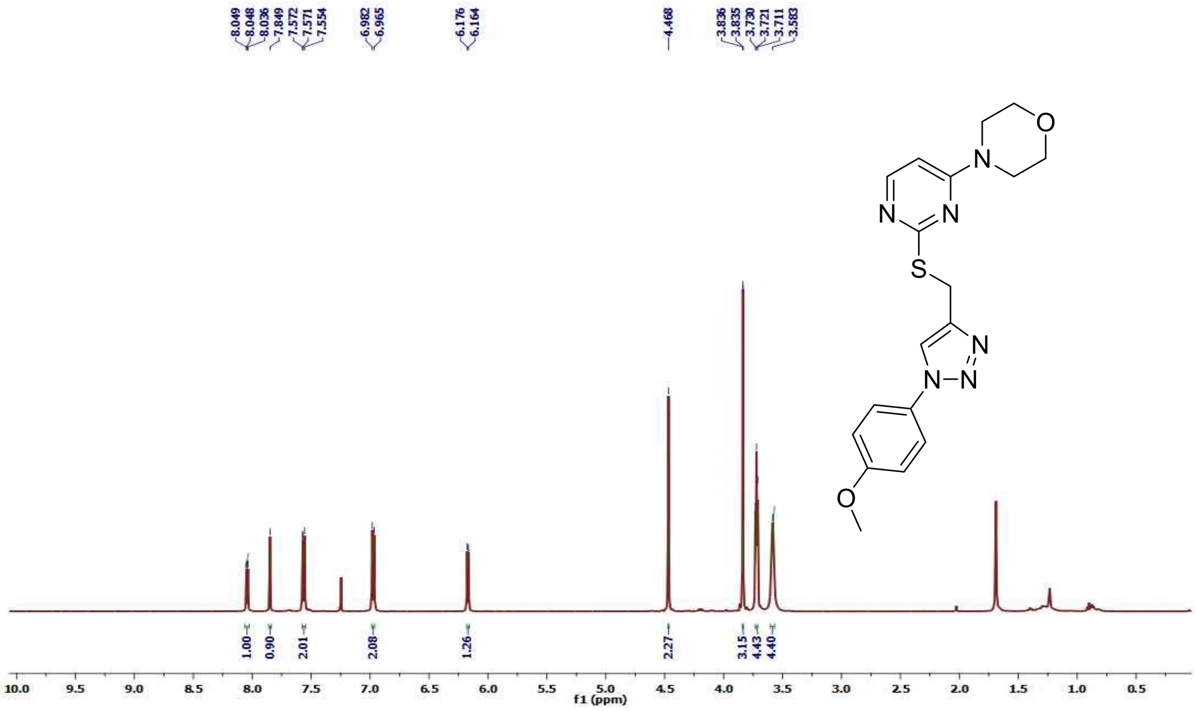

^{1}H NMR of 5g

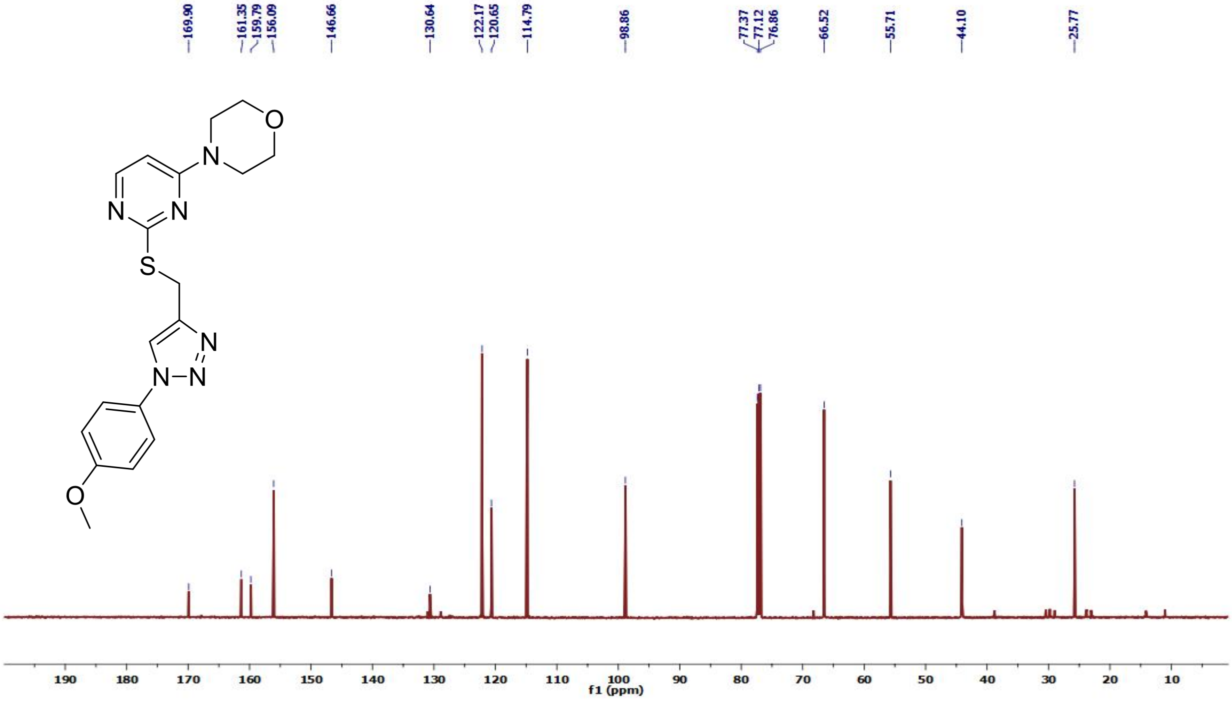

^{13}C NMR of 5g

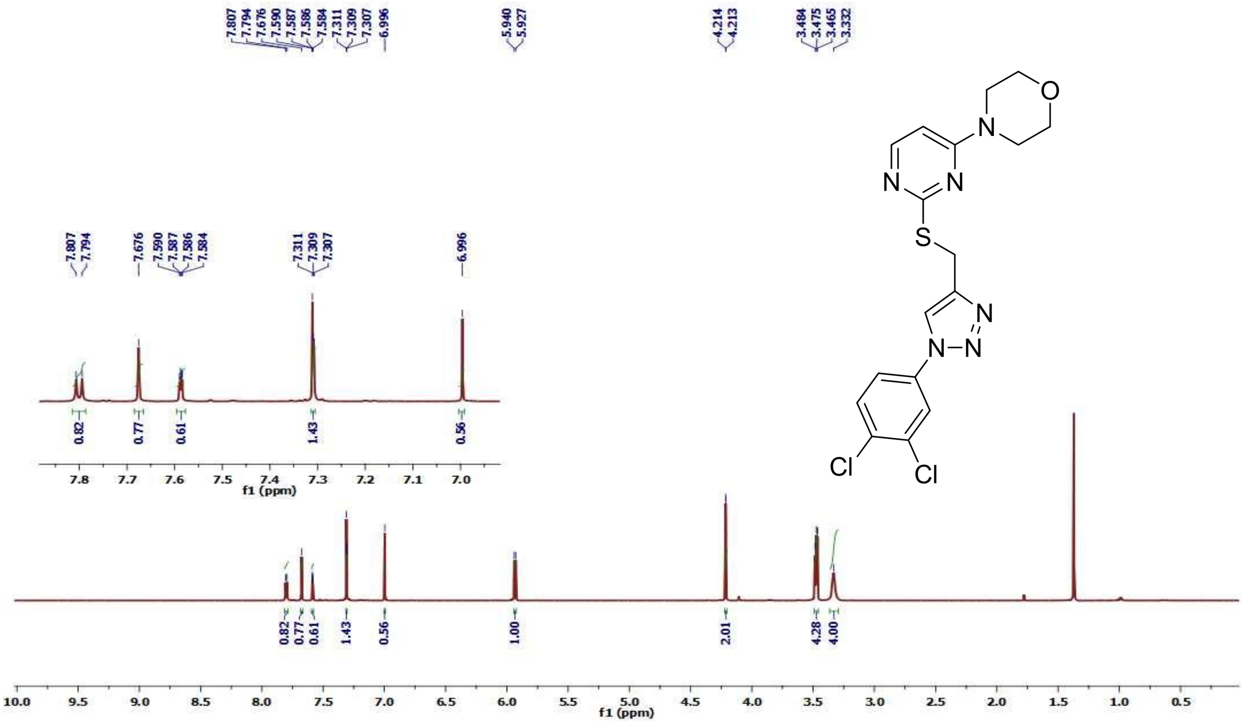

^{1}H NMR of 5h

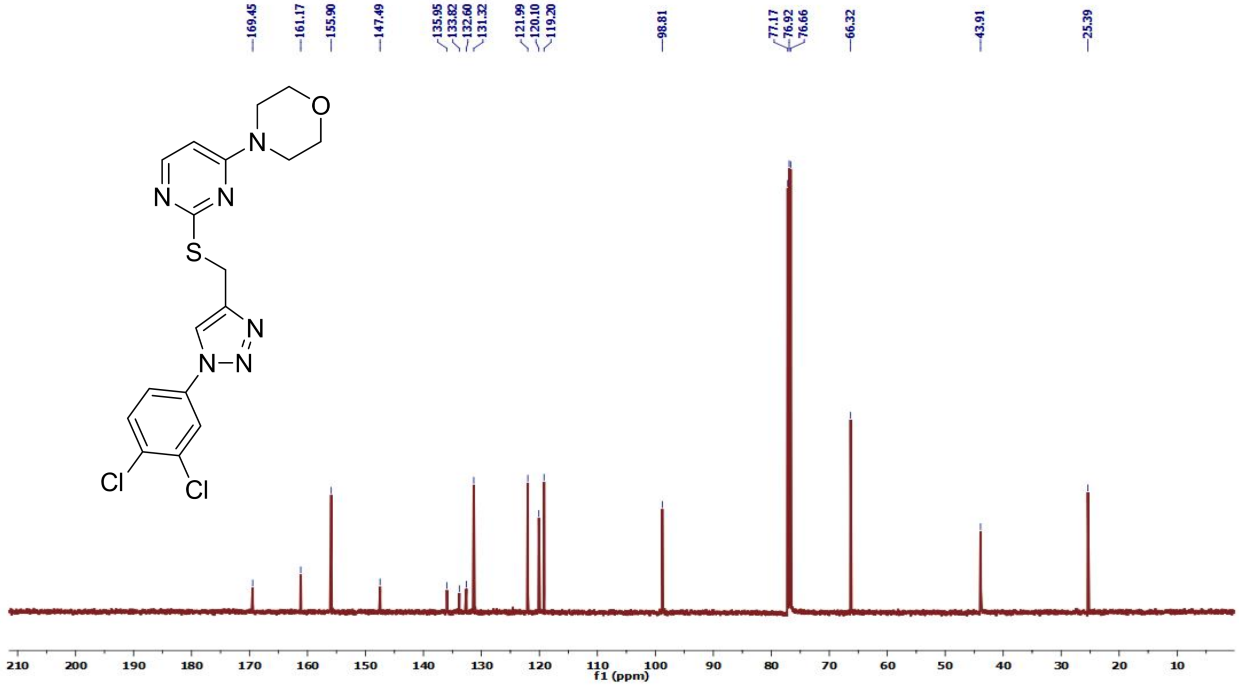

^{13}C NMR of 5h

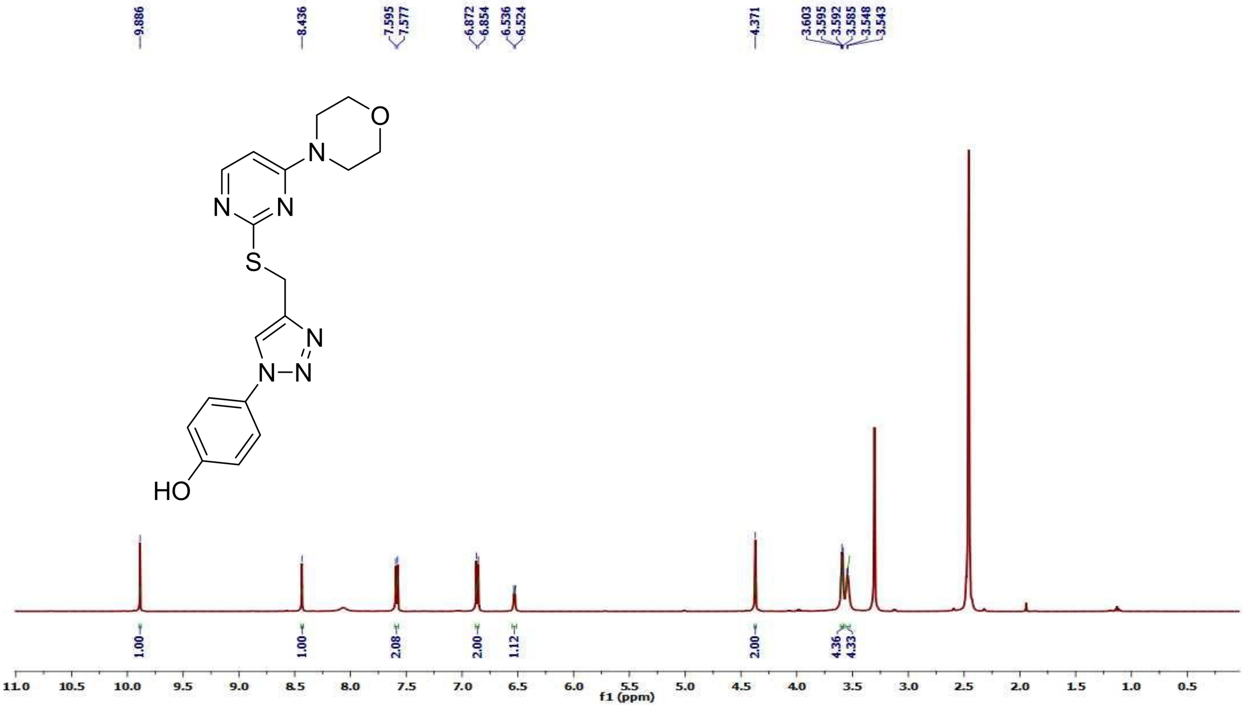

^{1}H NMR of 5i

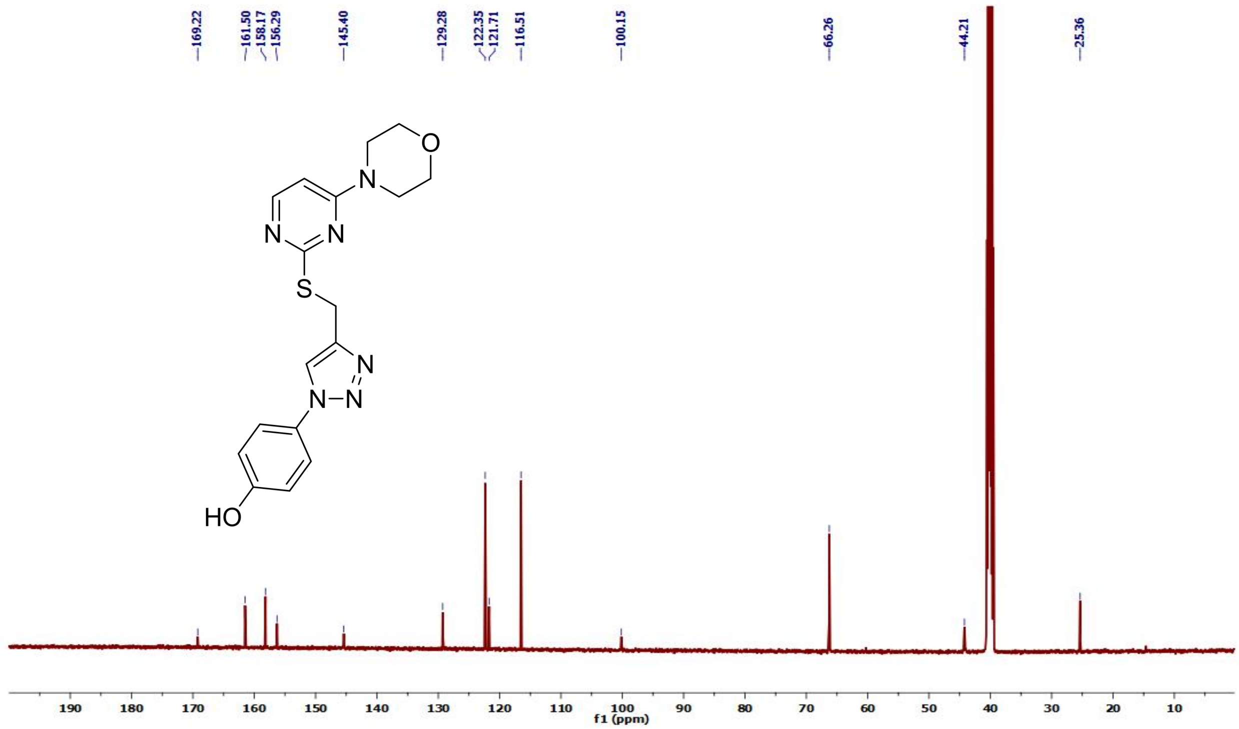

^{13}C NMR of 5i

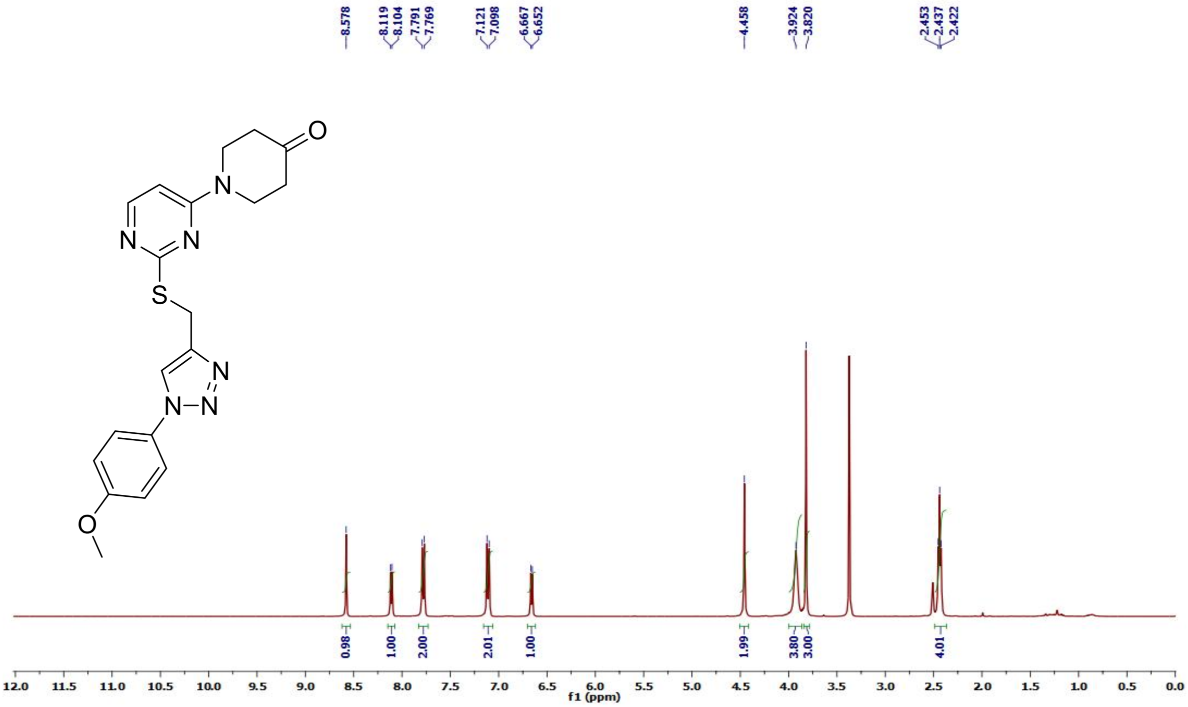

^{1}H NMR of 5j

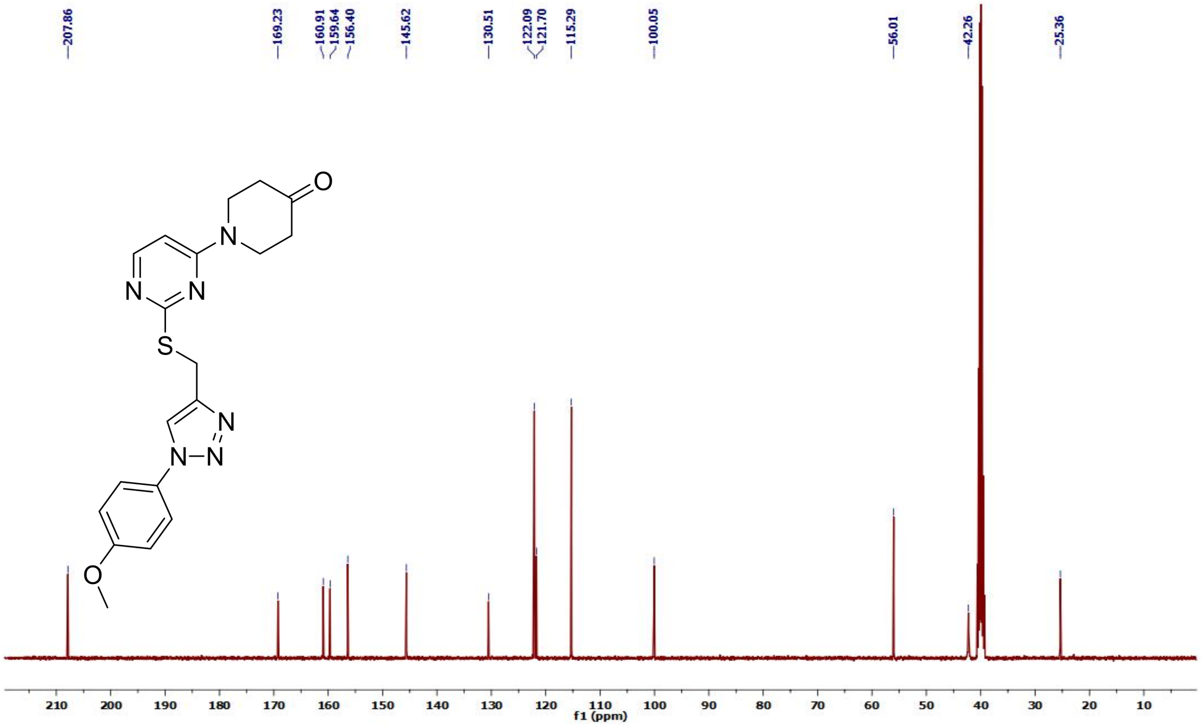

^{13}C NMR of 5j

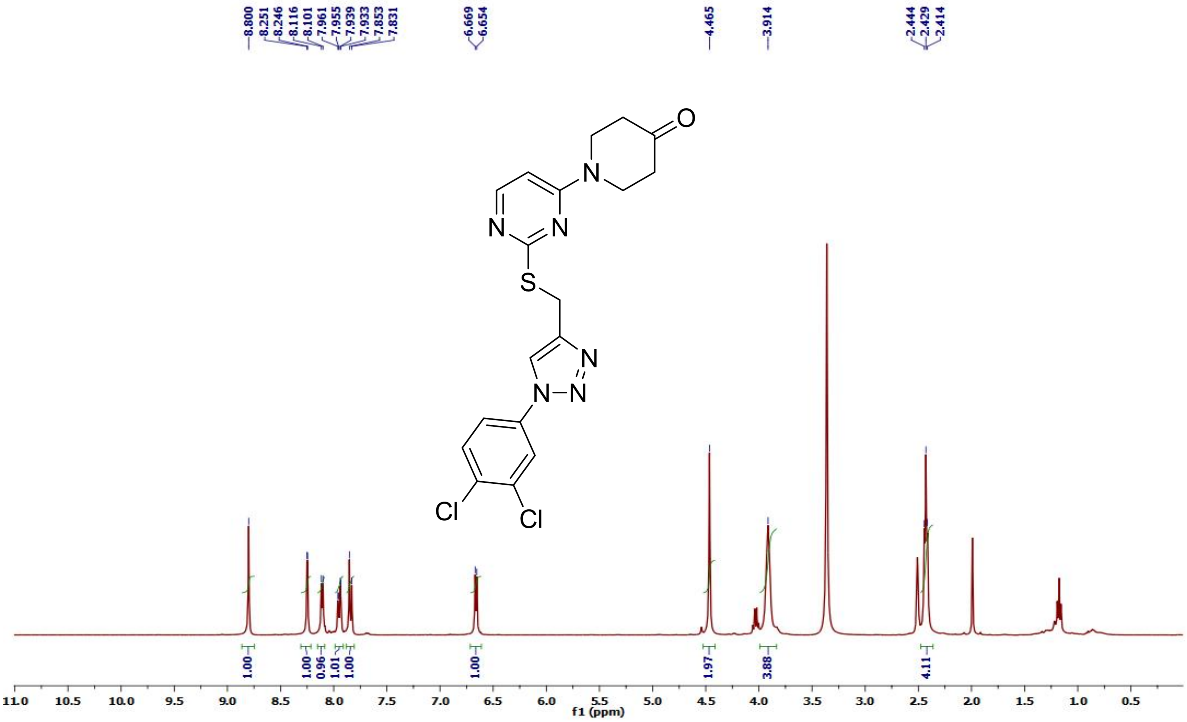

^{1}H NMR of 5k

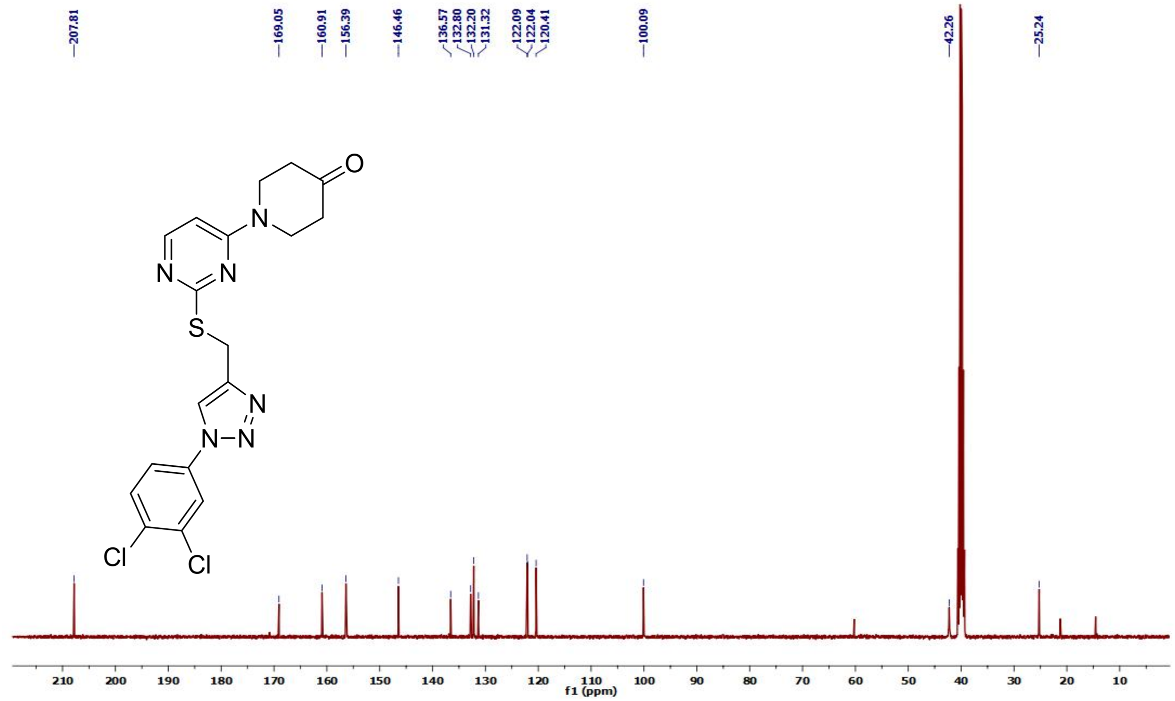

^{13}C NMR of 5k

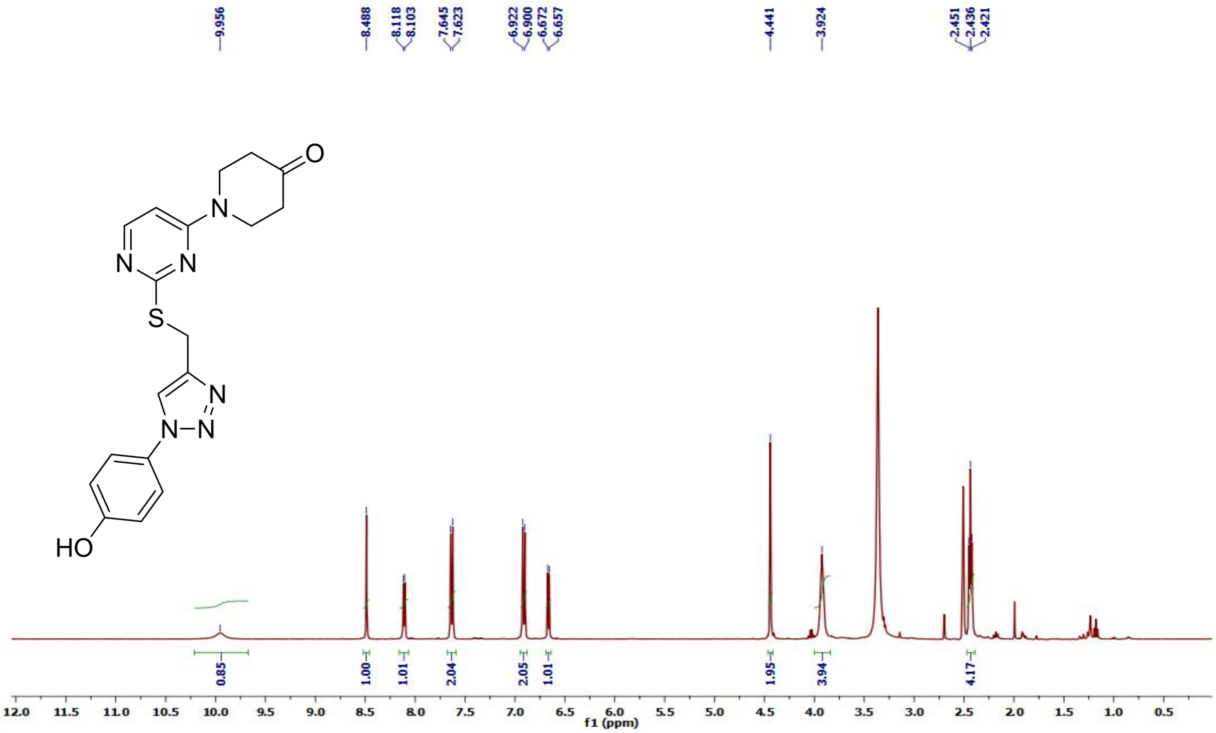

^{1}H NMR of 5l

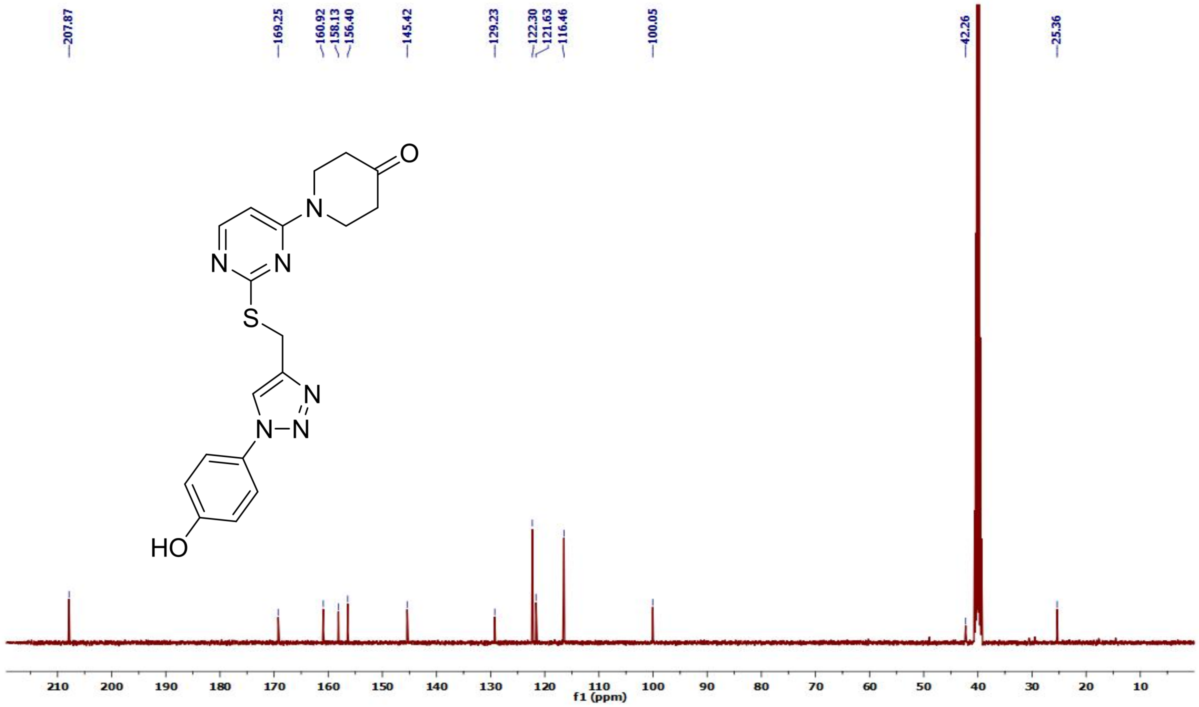

^{13}C NMR of 5l

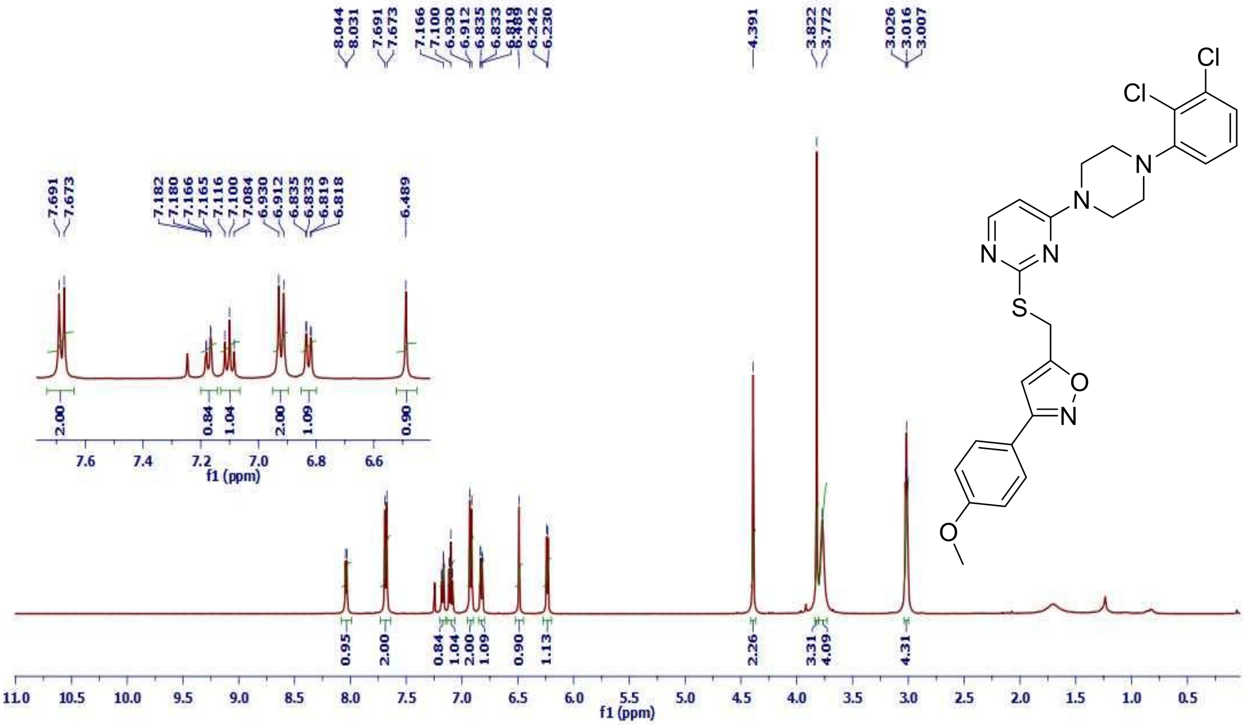

^{1}H NMR of 6a

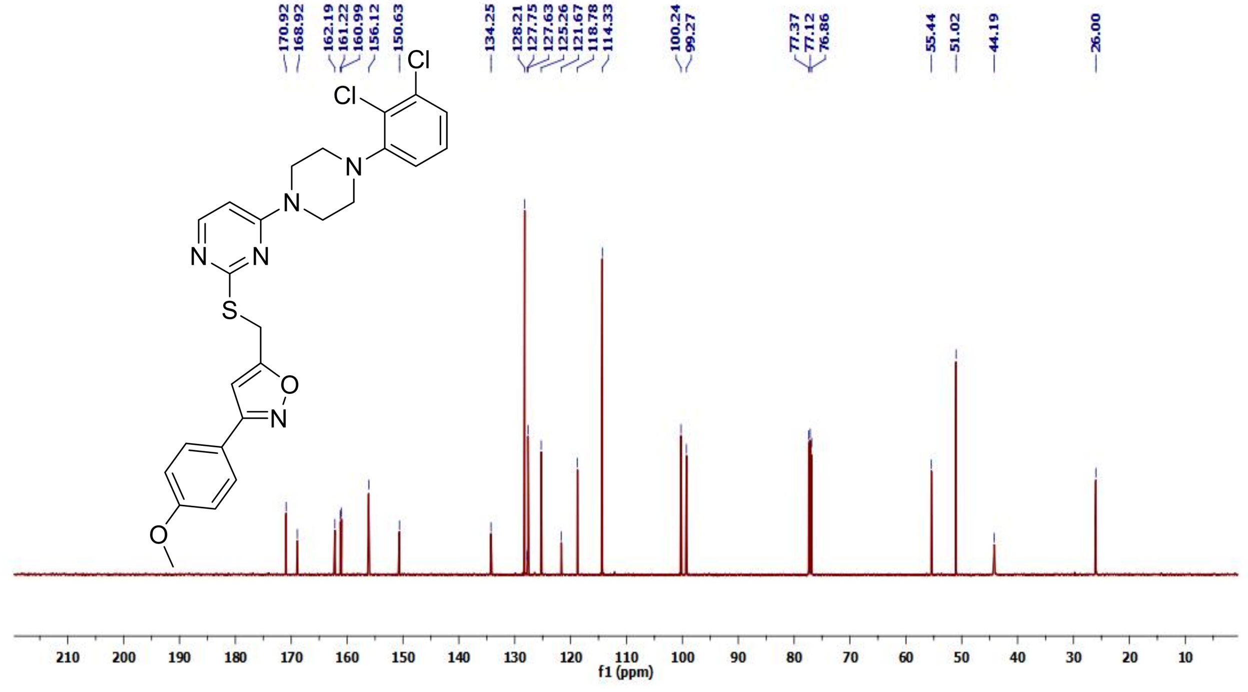

^{13}C NMR of 6a

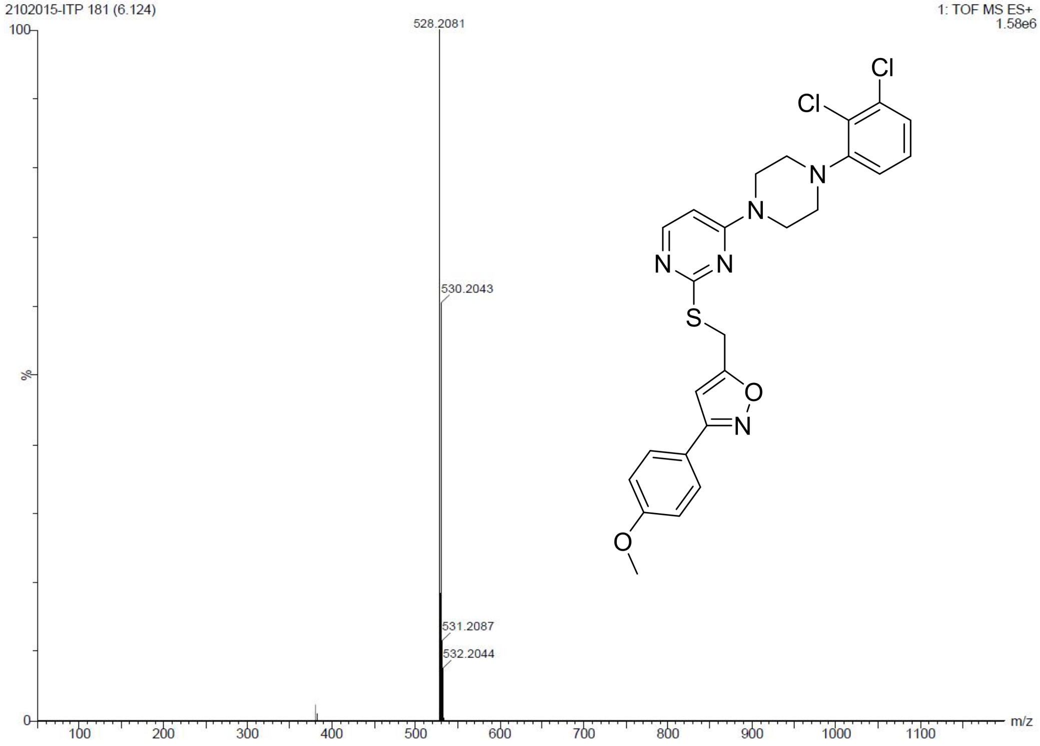

Mass spectrum of 6a

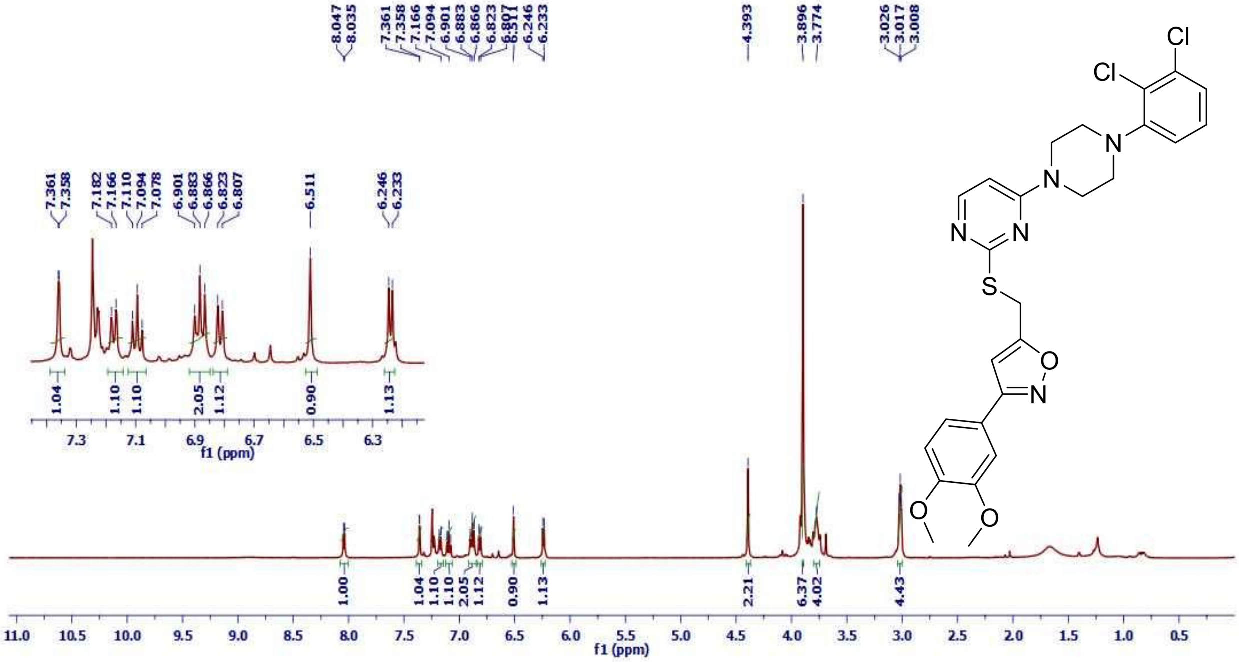

^{1}H NMR of 6b

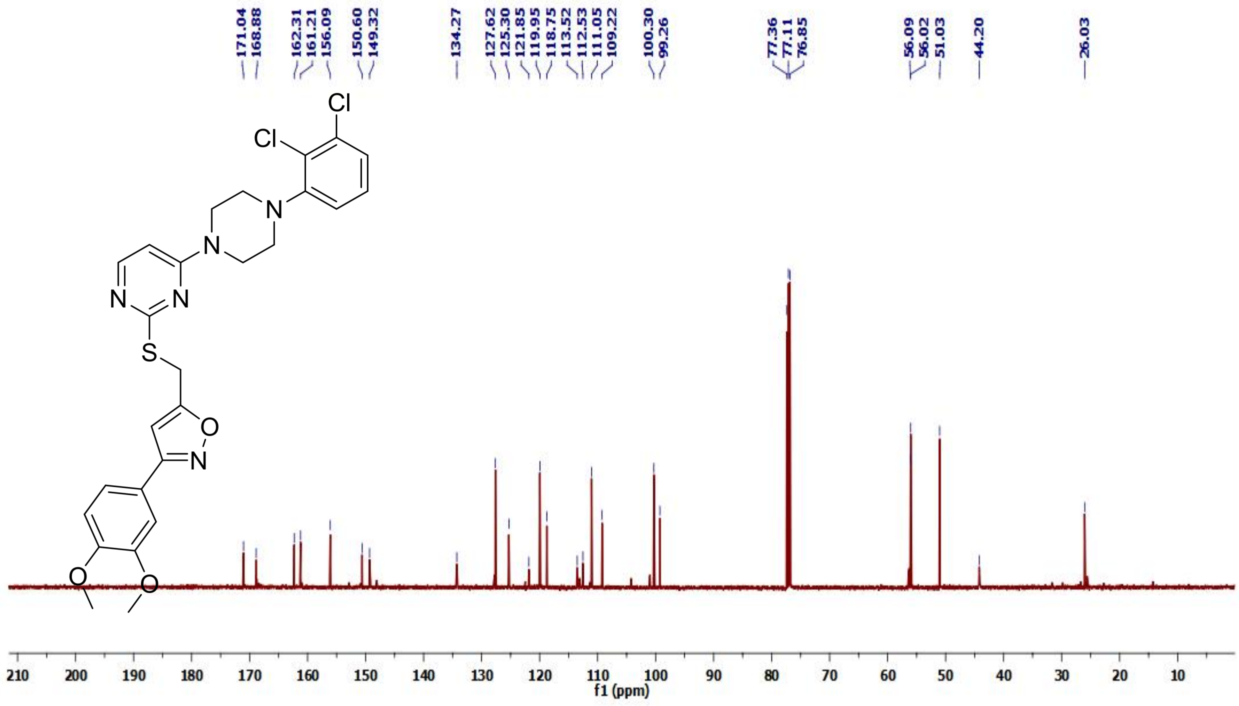

^{13}C NMR of 6b

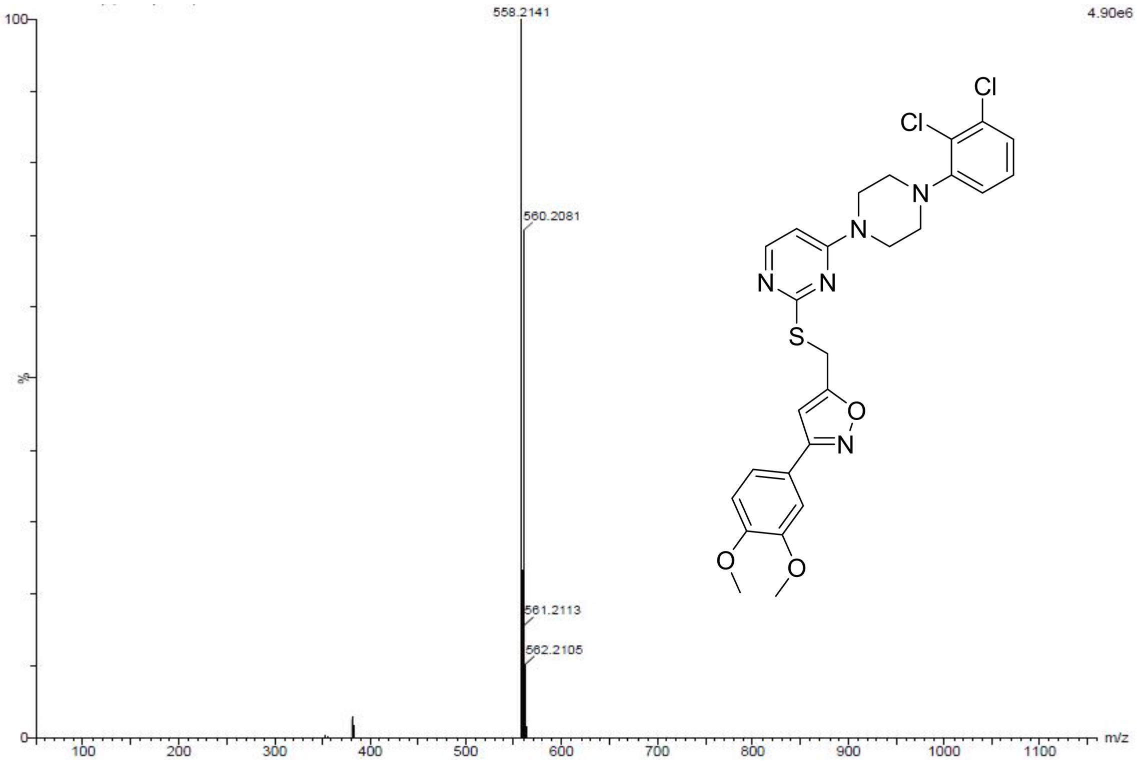

Mass spectrum of 6b

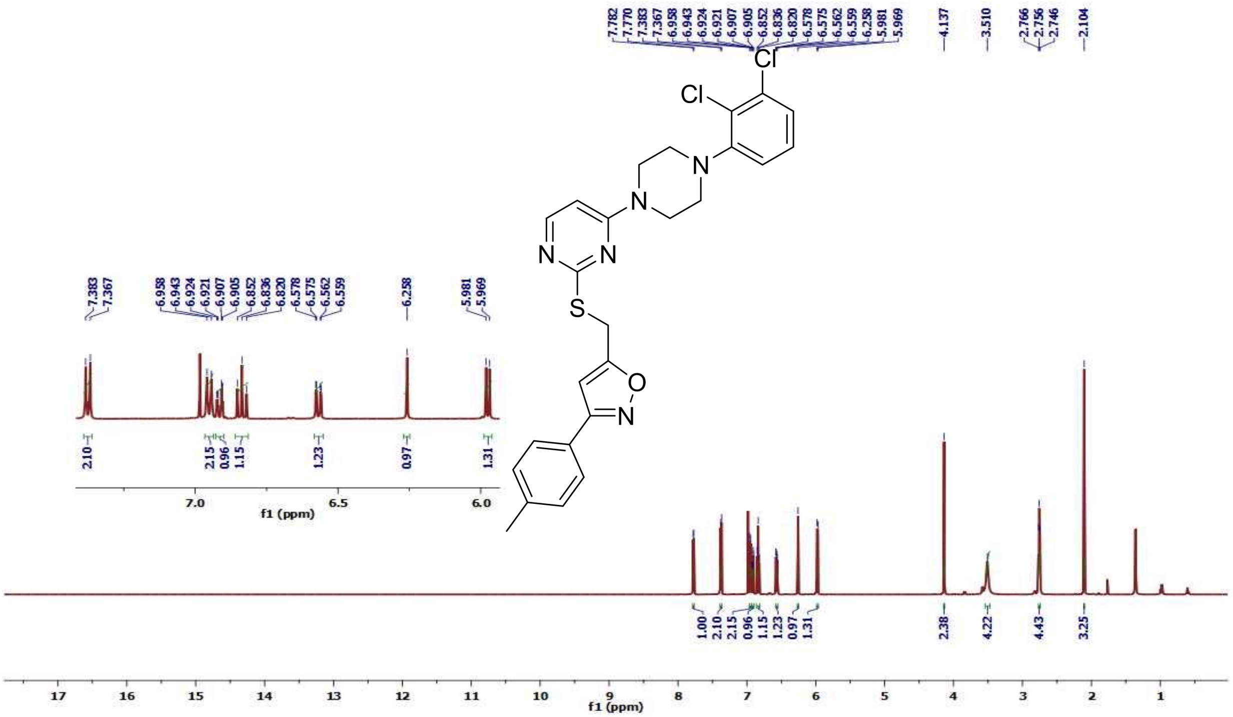

^{1}H NMR of 6c

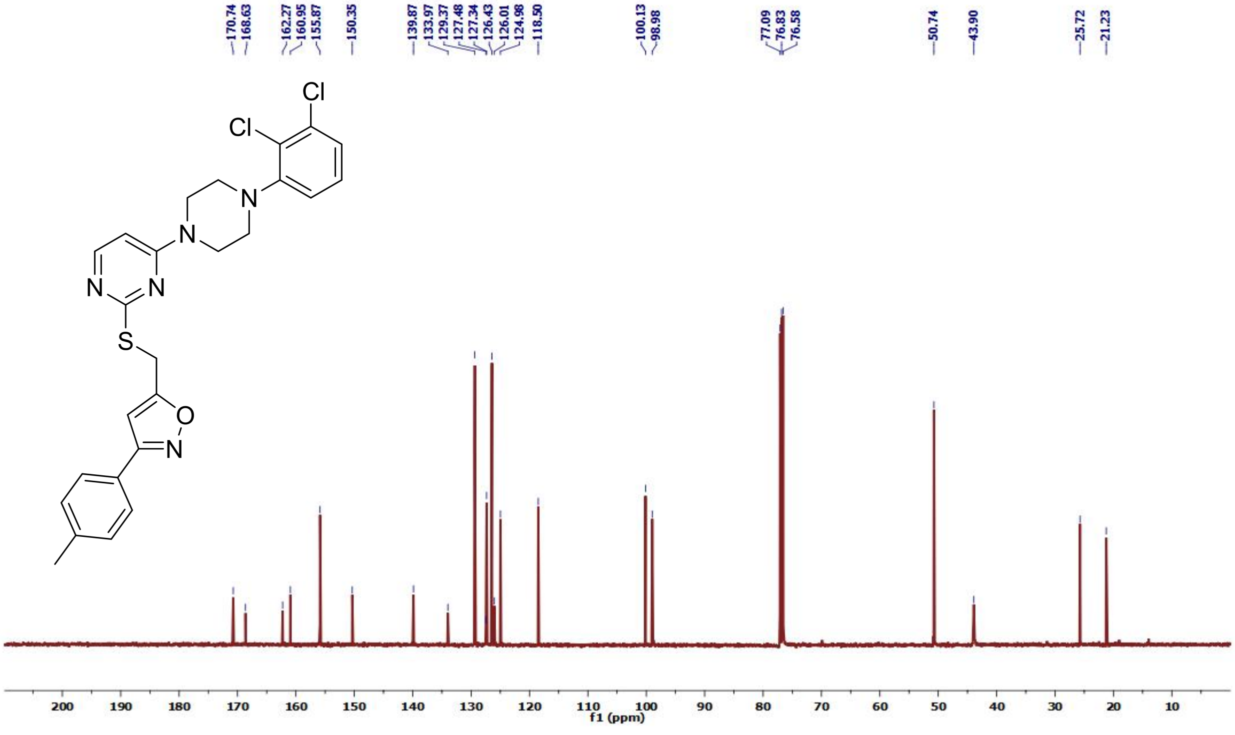

^{13}C NMR of 6c

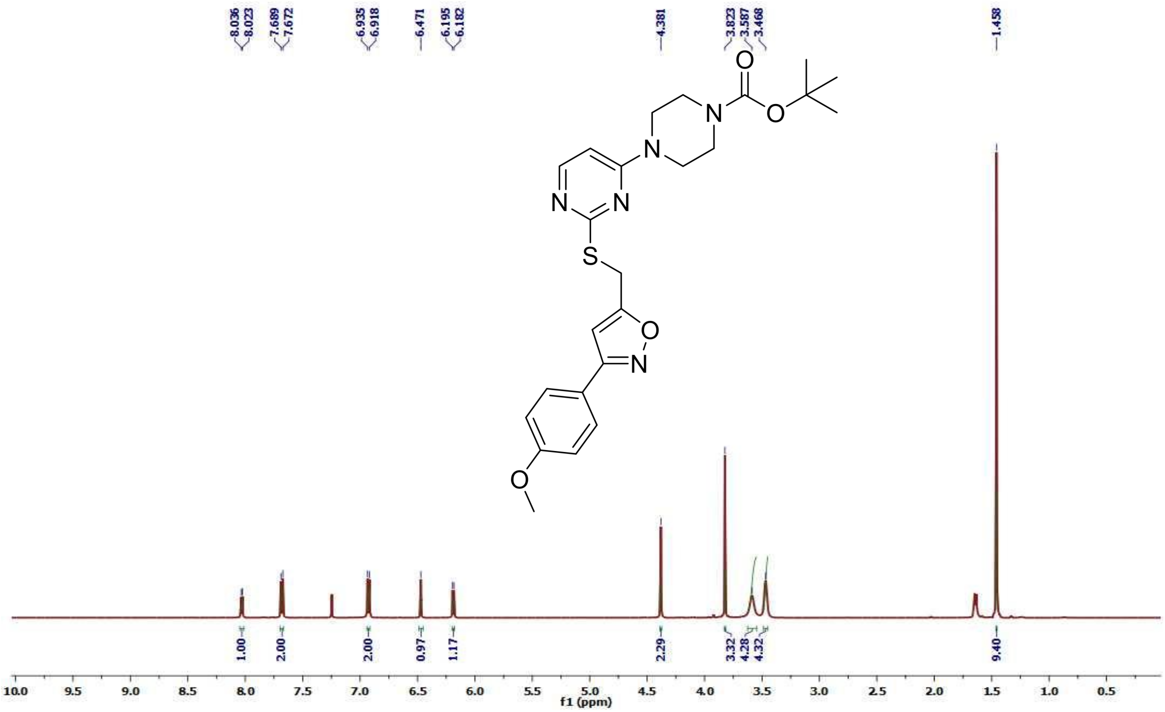

^{1}H NMR of 6d

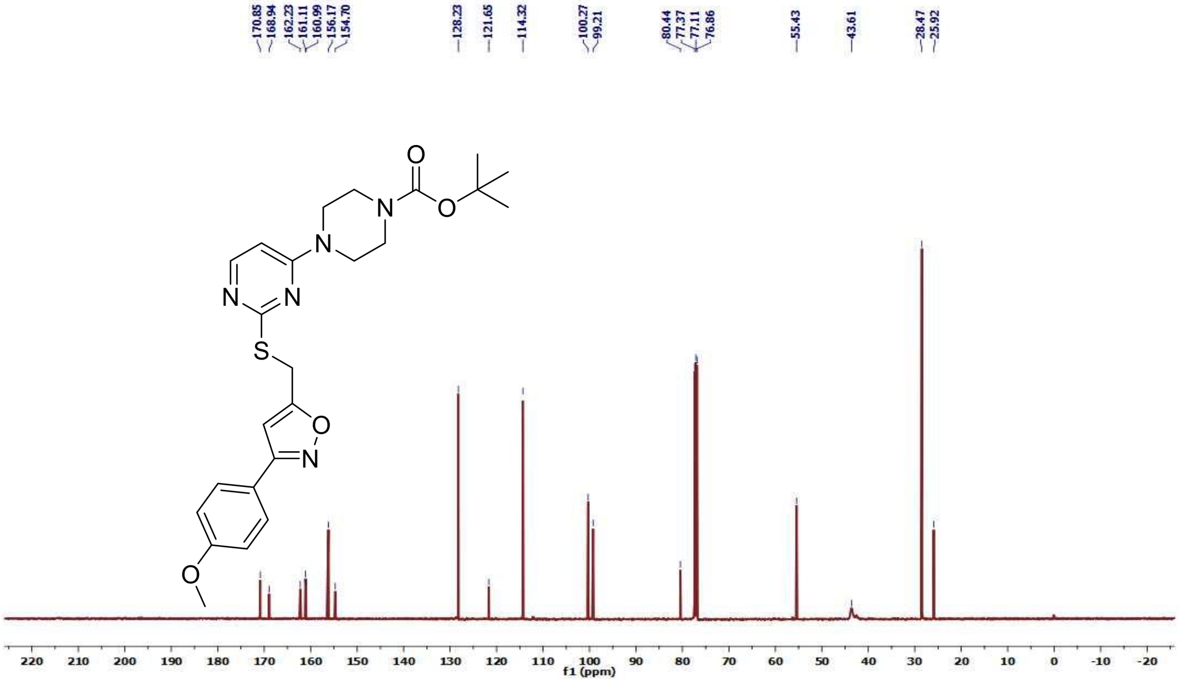

^{13}C NMR of 6d

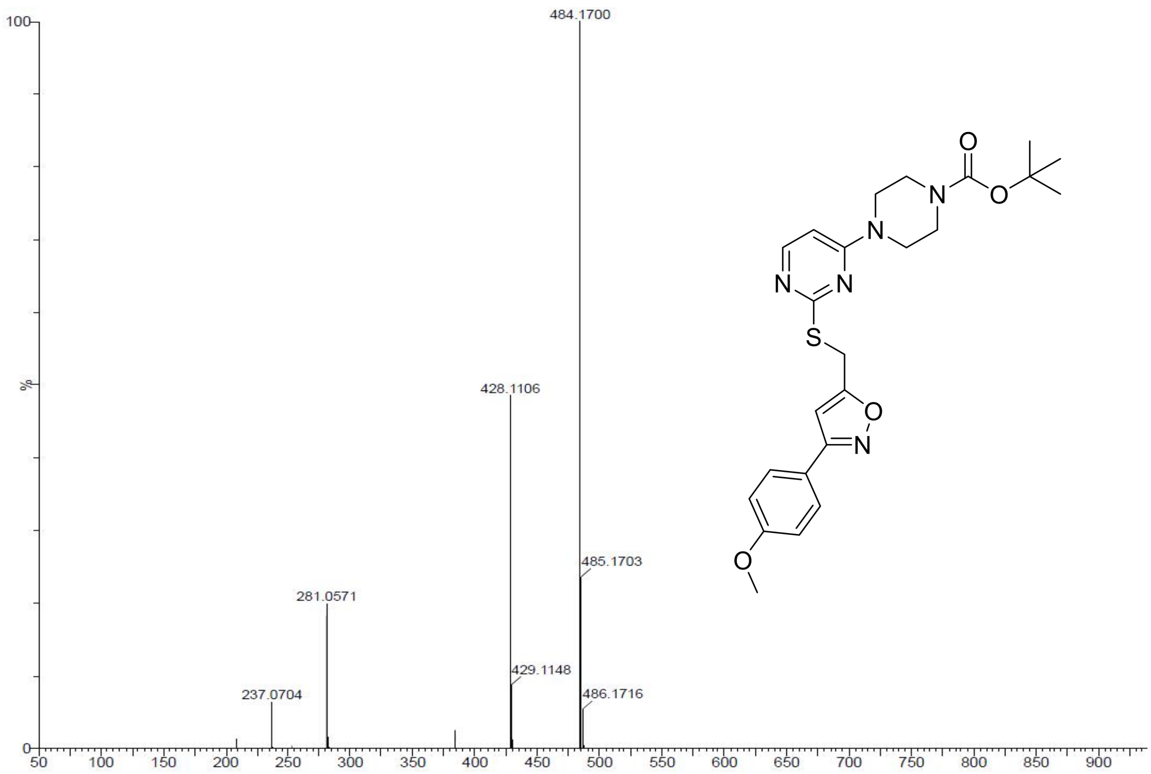

Mass spectrum of 6d

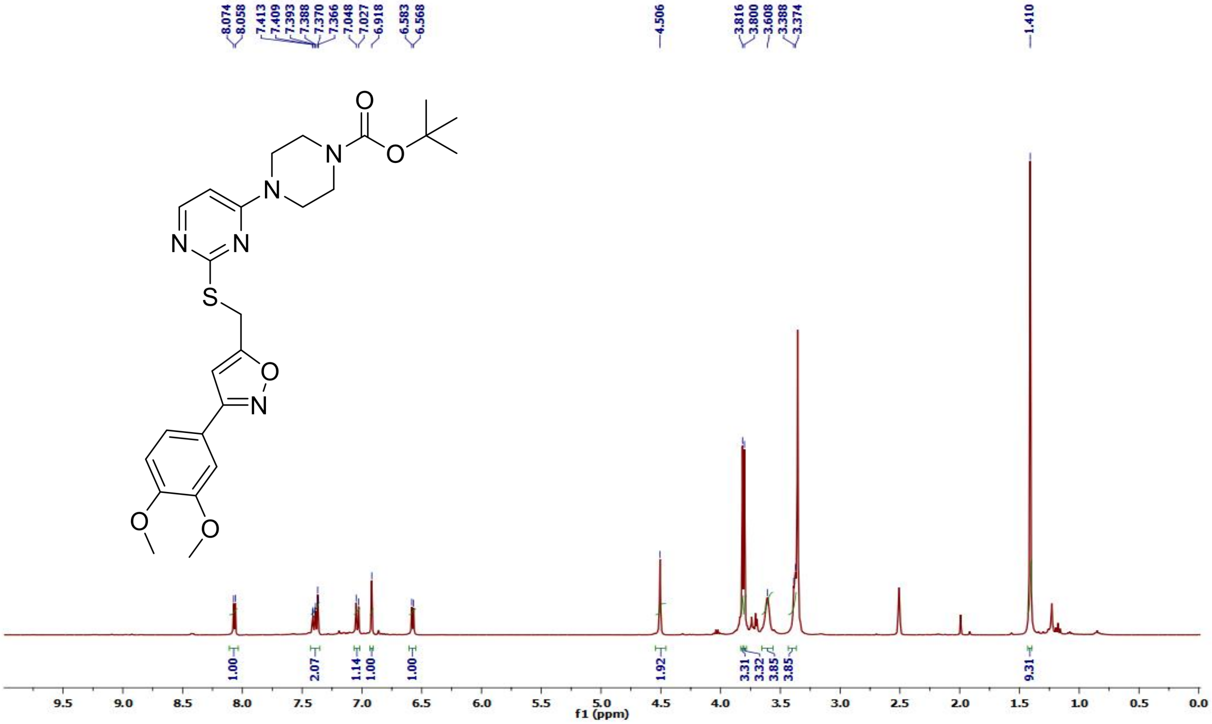

^{1}H NMR of 6e

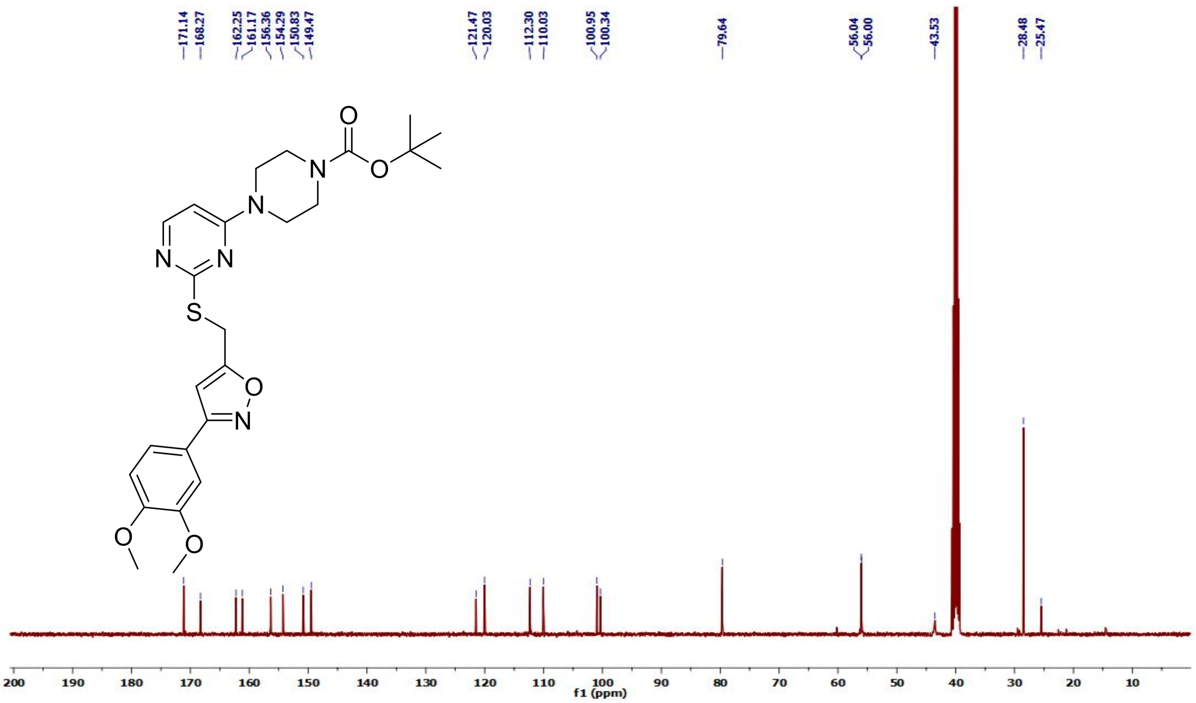

^{13}C NMR of 6e

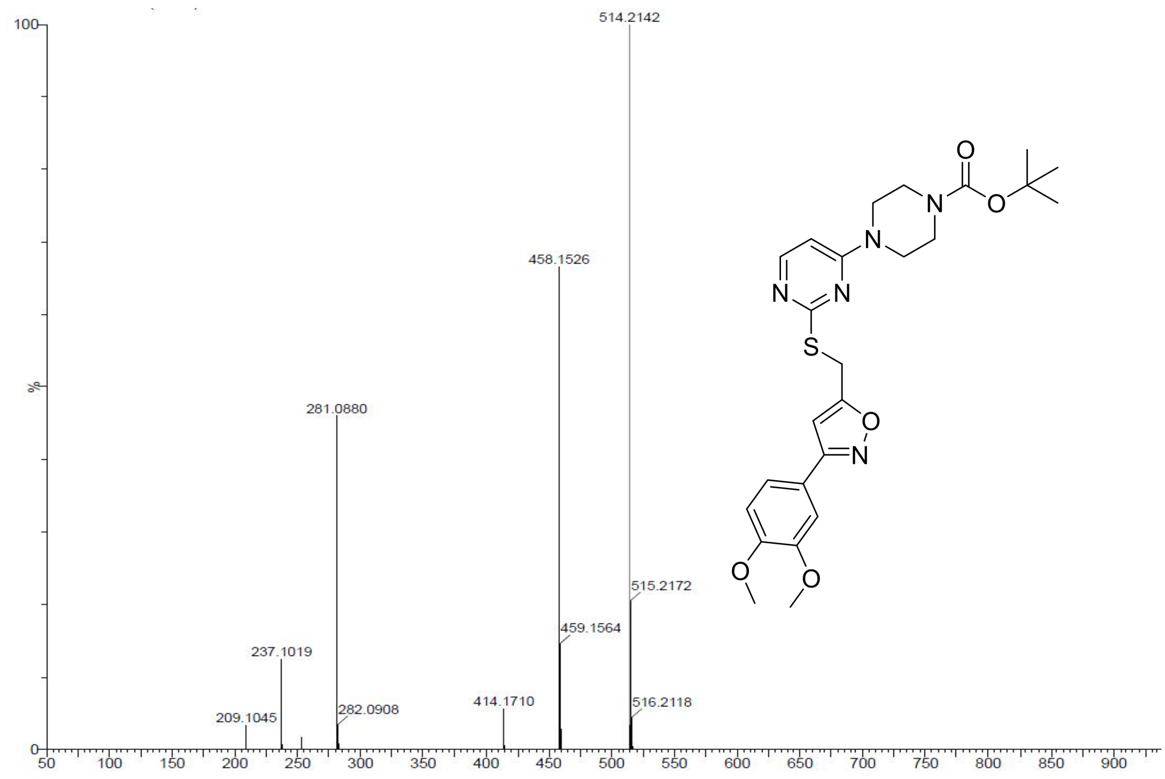

Mass spectrum of 6e

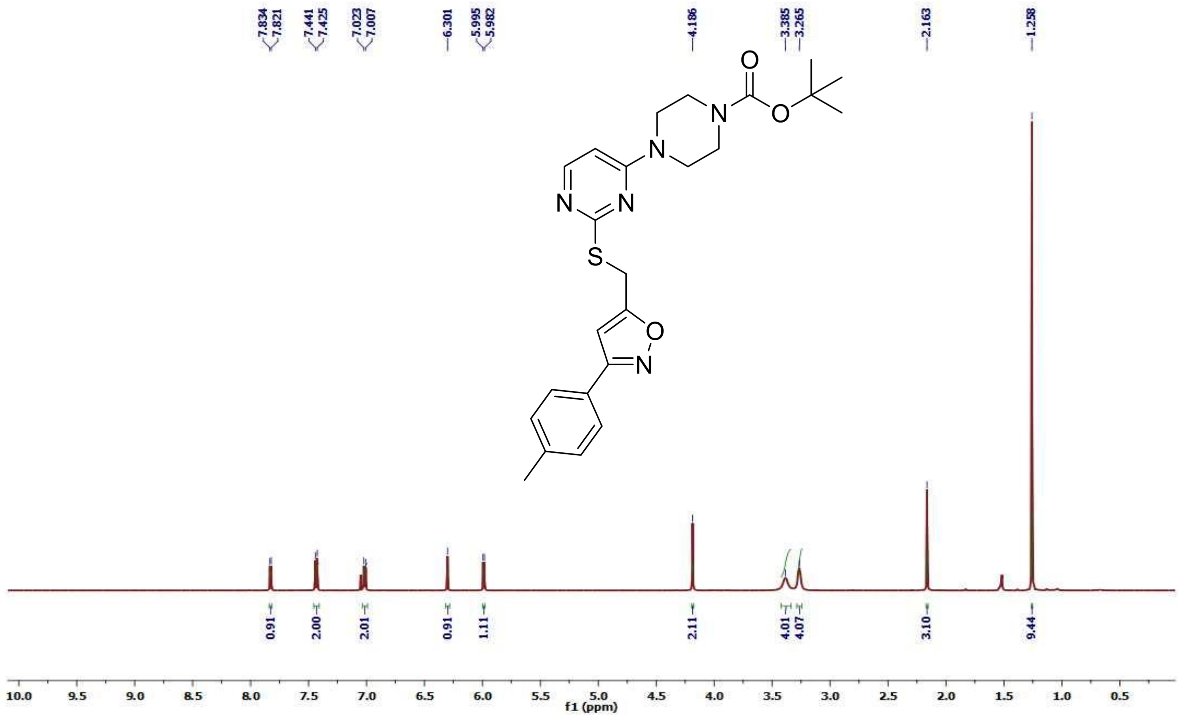

^{1}H NMR of 6f

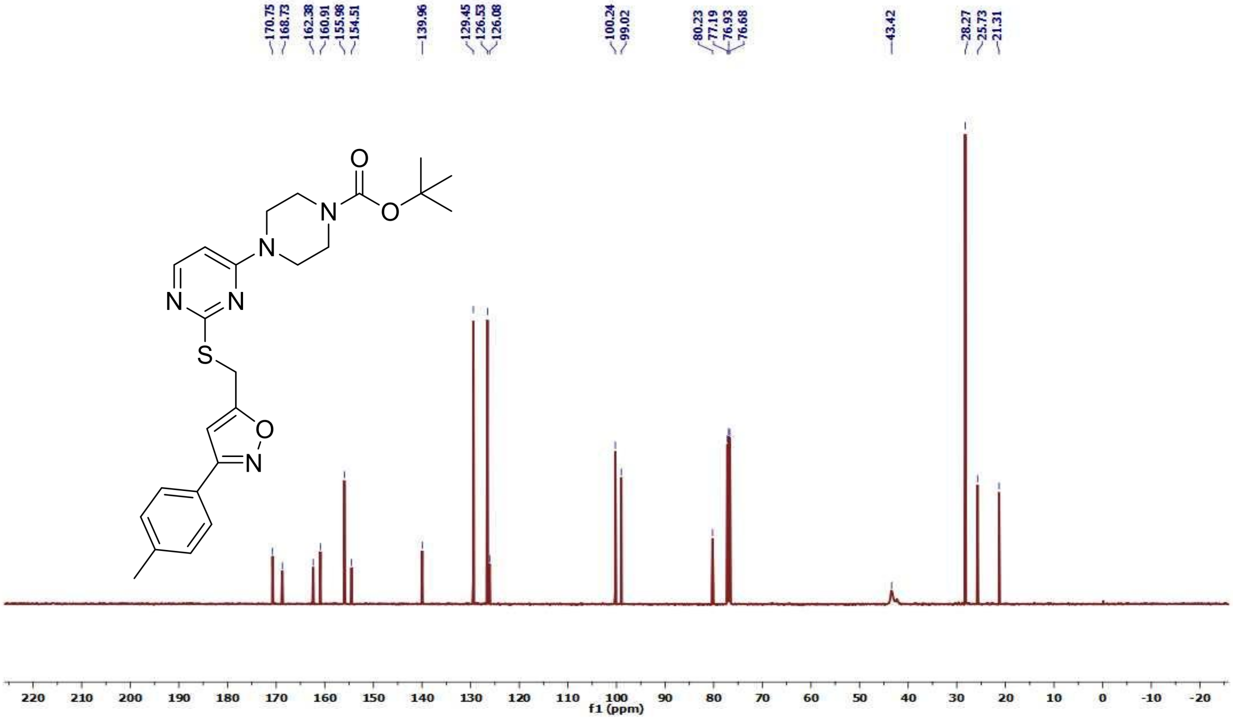

^{13}C NMR of 6f

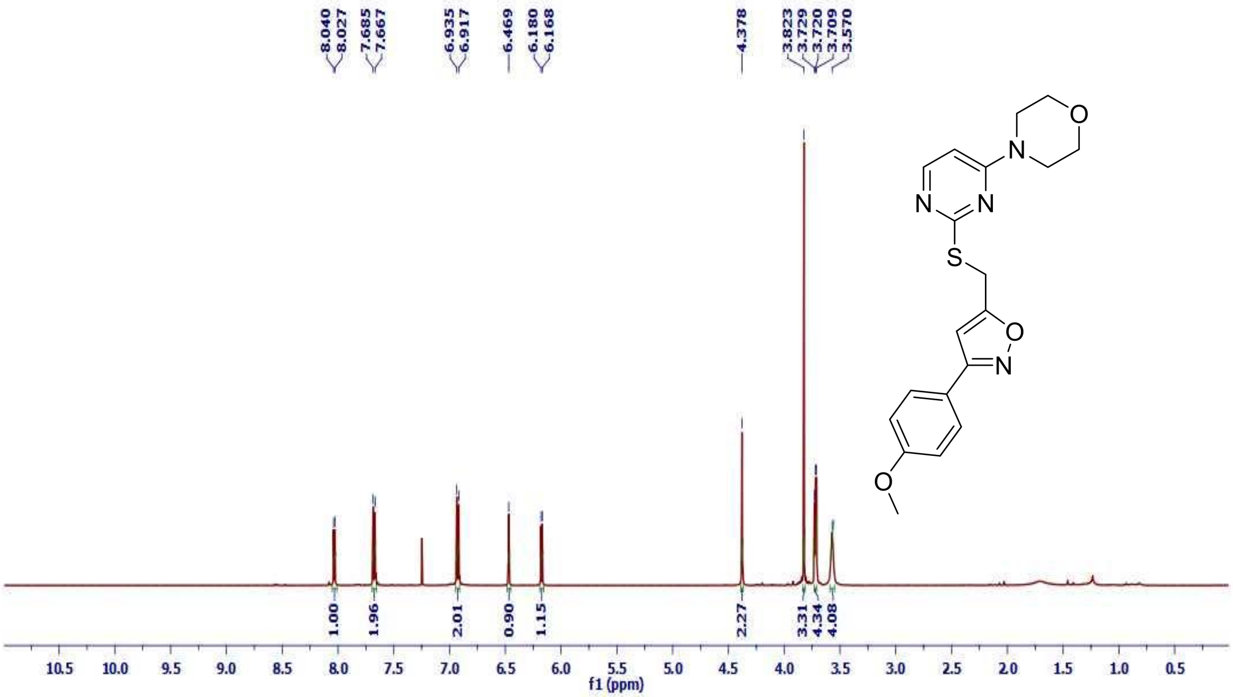

^{1}H NMR of 6g

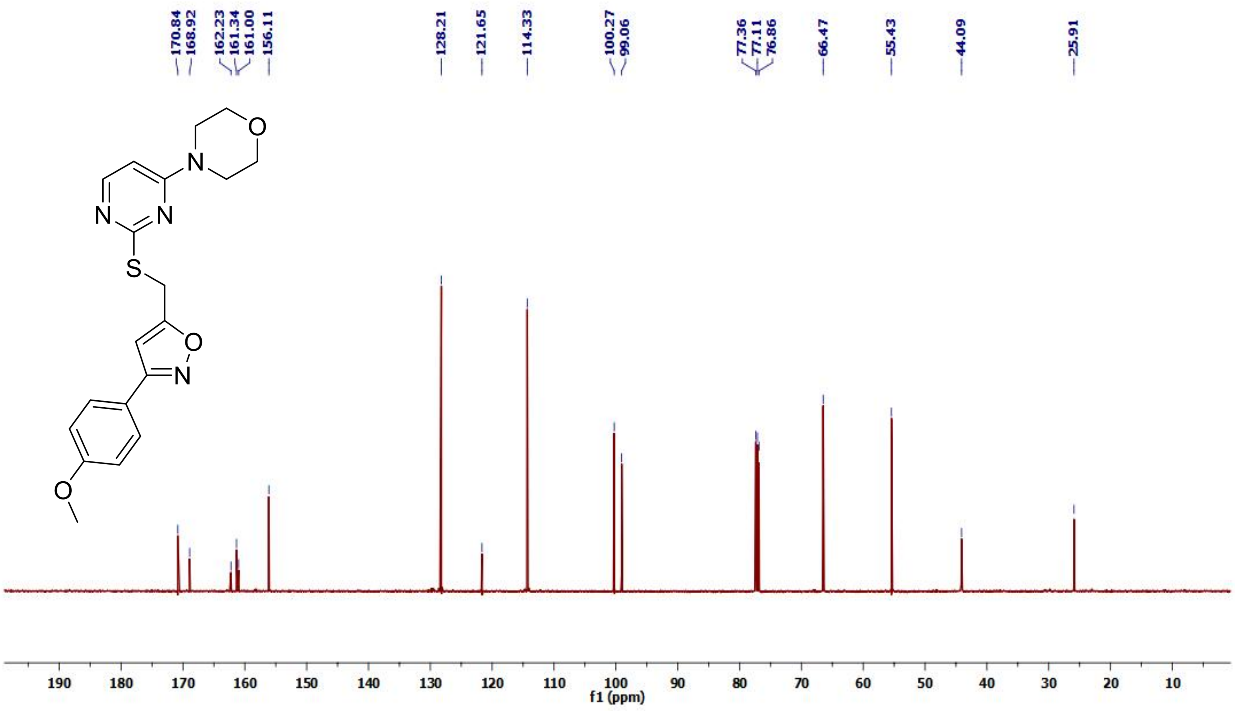

^{13}C NMR of 6g

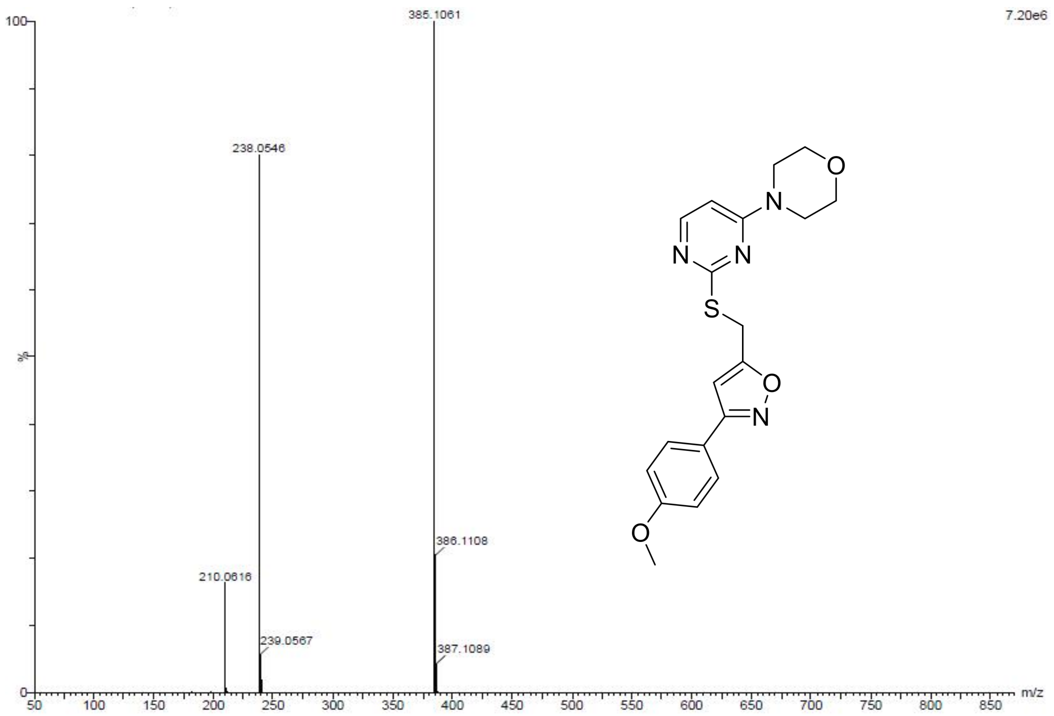

Mass spectrum of 6g

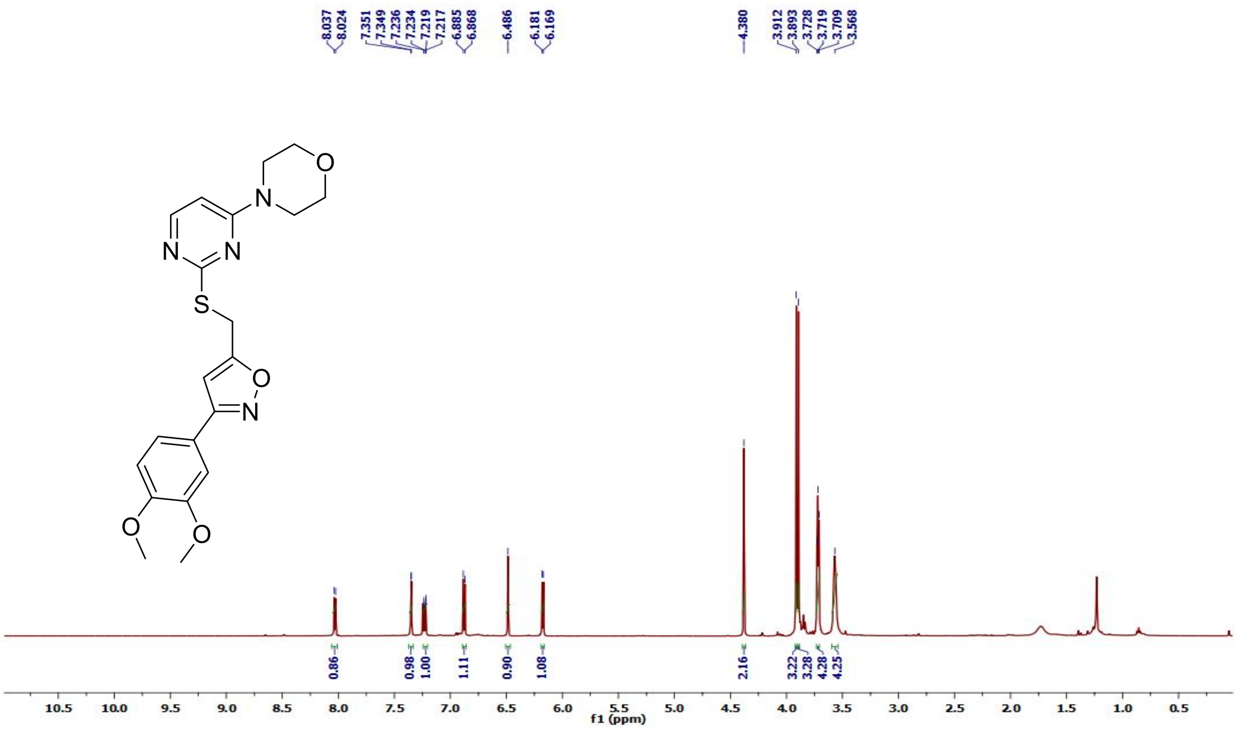

^{1}H NMR of 6h

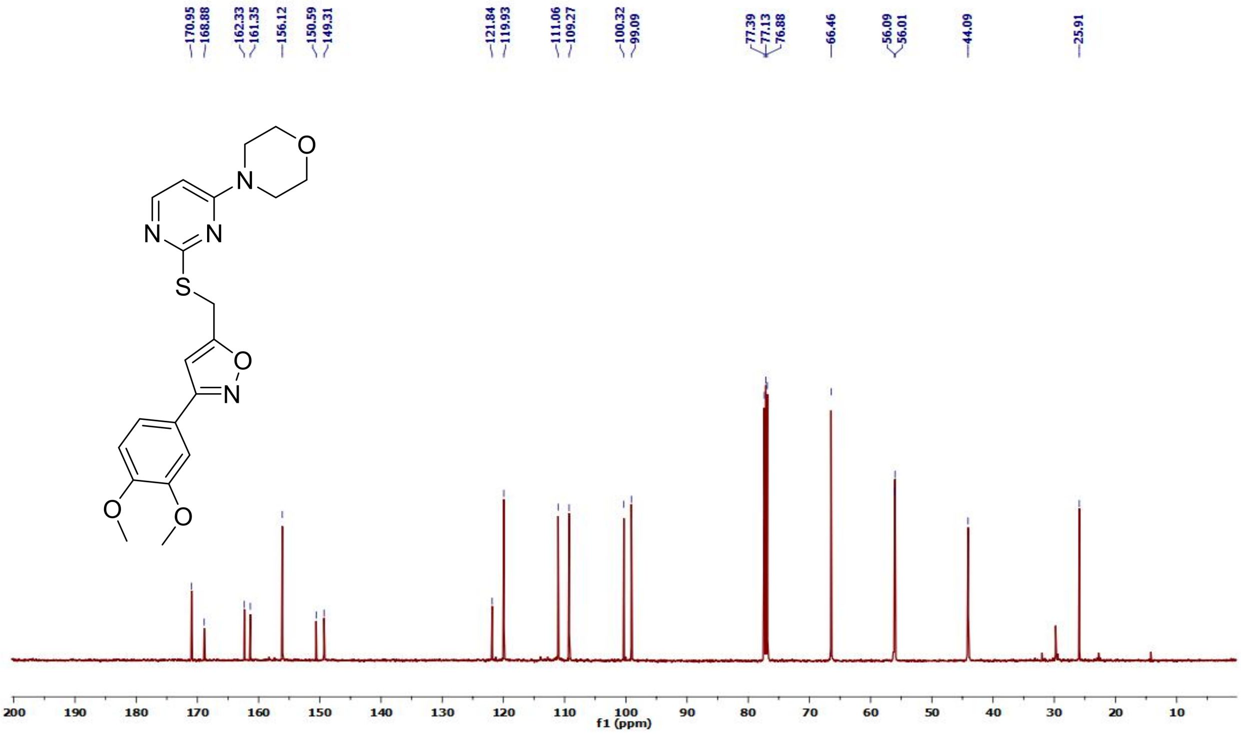

^{13}C NMR of 6h

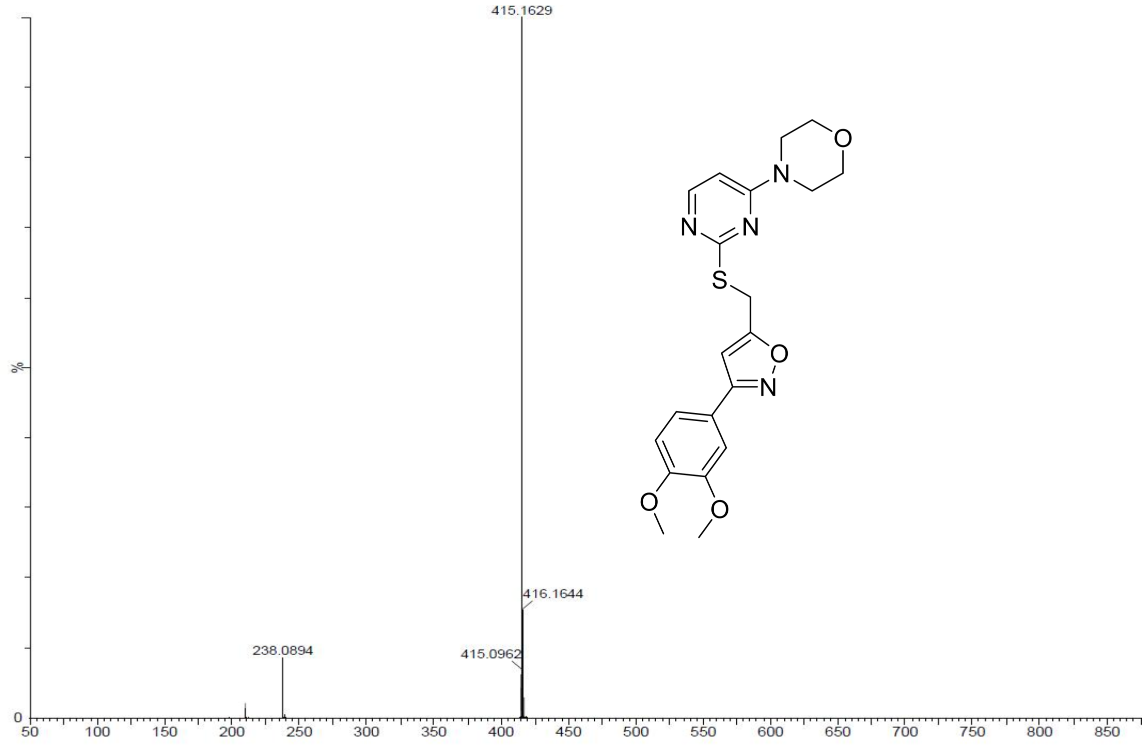

Mass spectrum of 6h

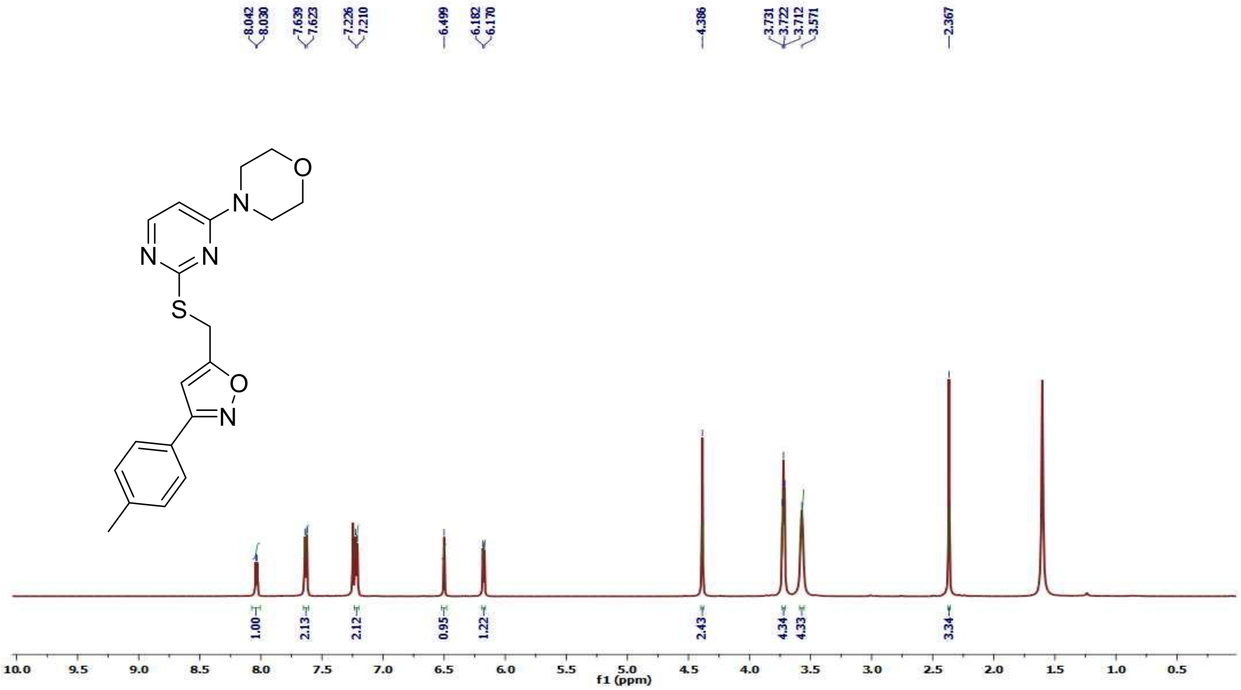

^{1}H NMR of 6i

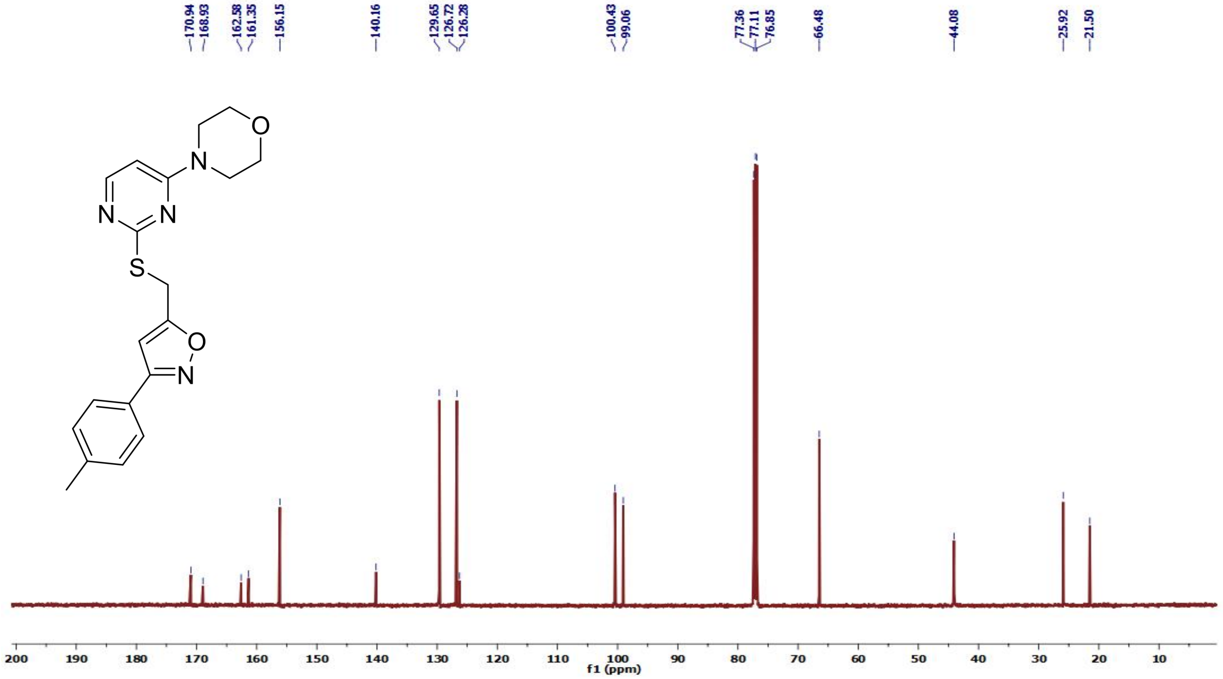

^{13}C NMR of 6i

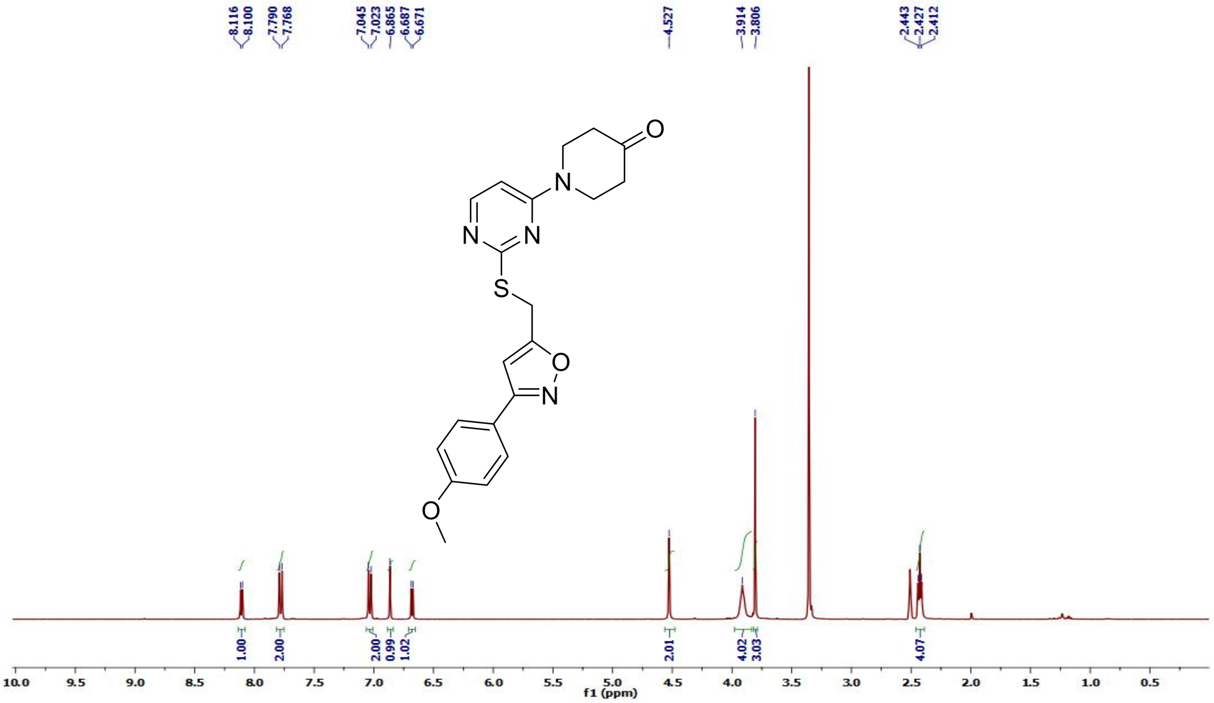

^{1}H NMR of 6j

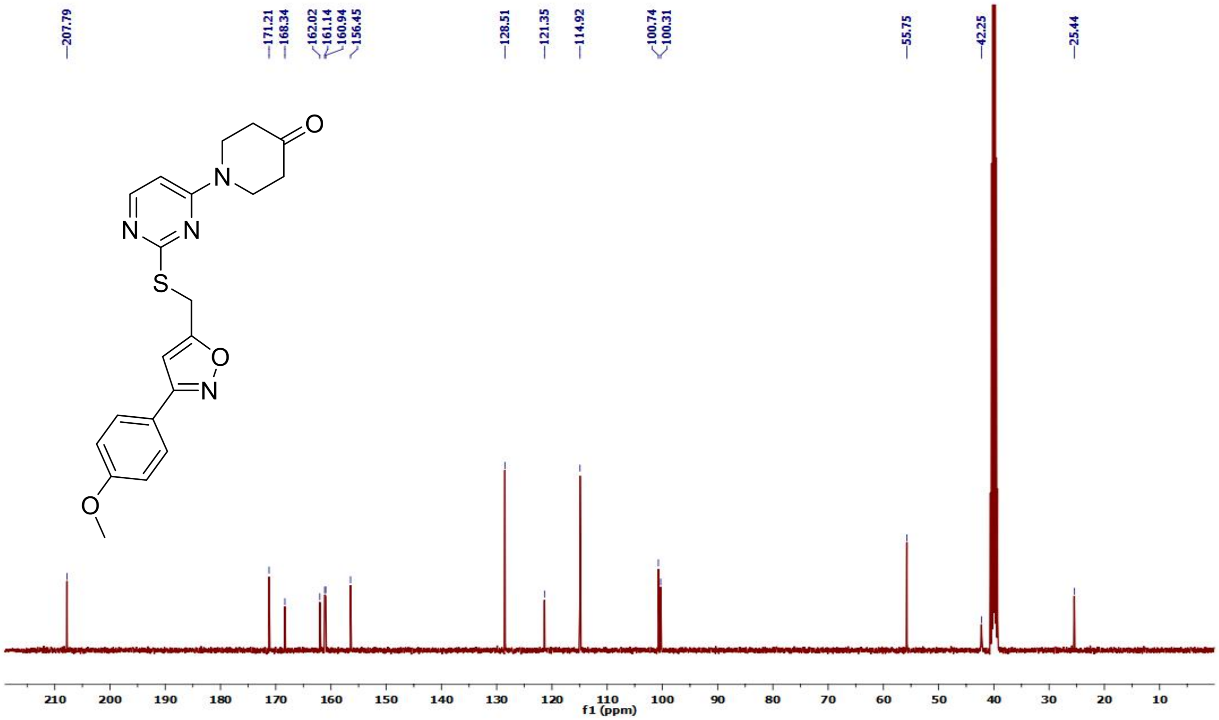

^{13}C NMR of 6j

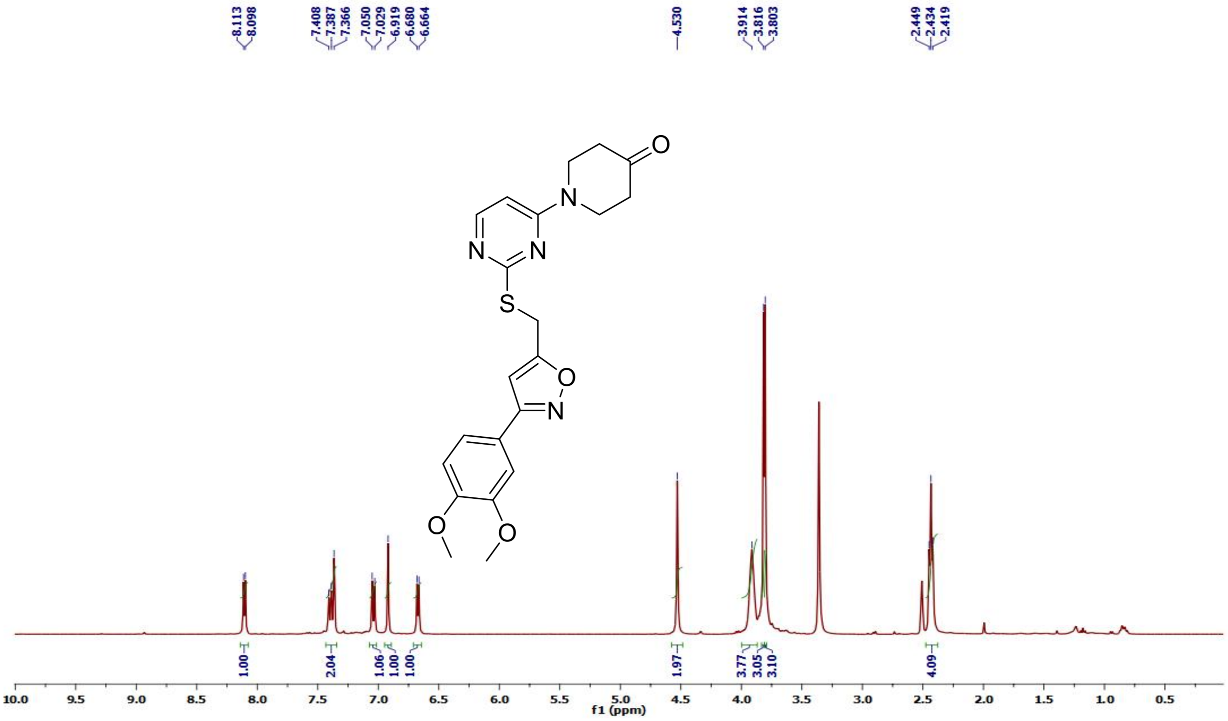

^{1}H NMR of 6k

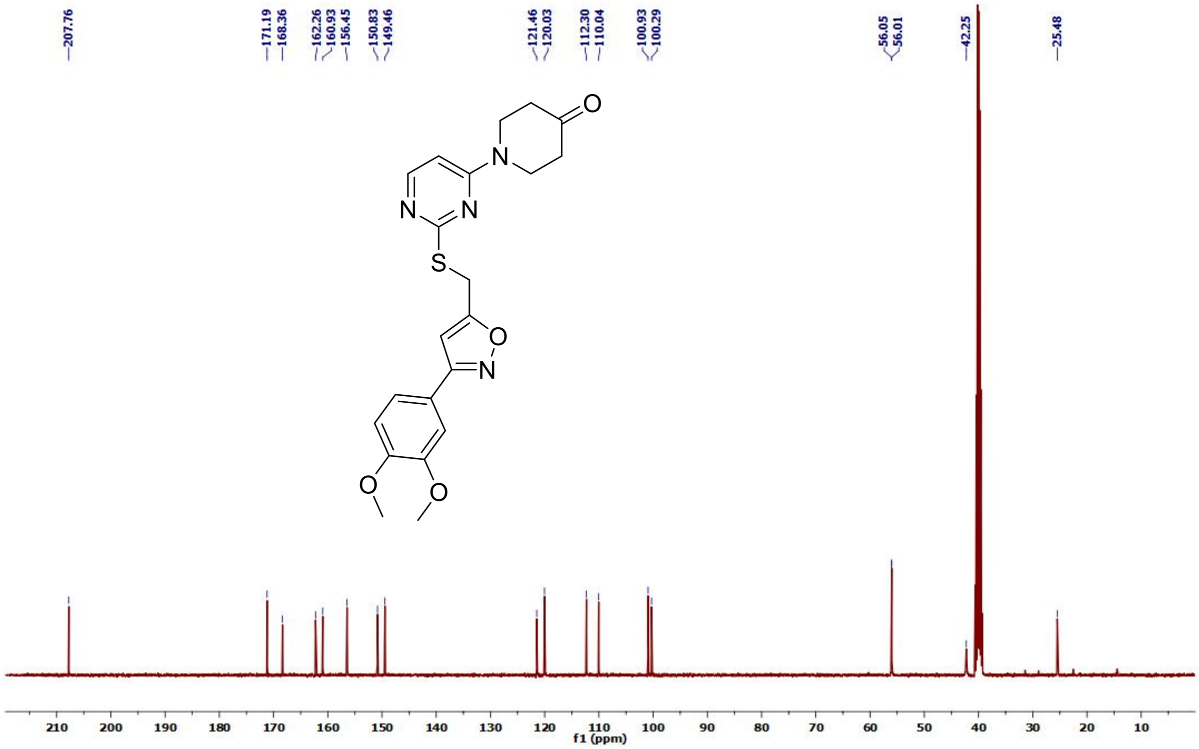

^{13}C NMR of 6k

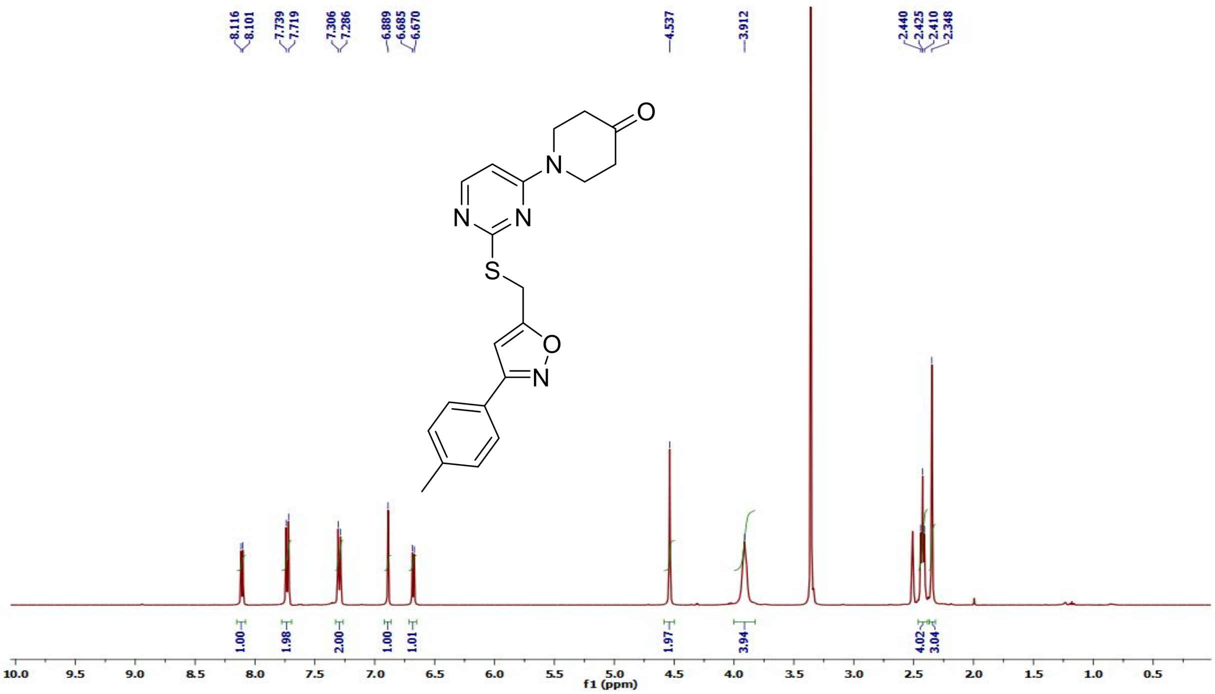

^{1}H NMR of 6l

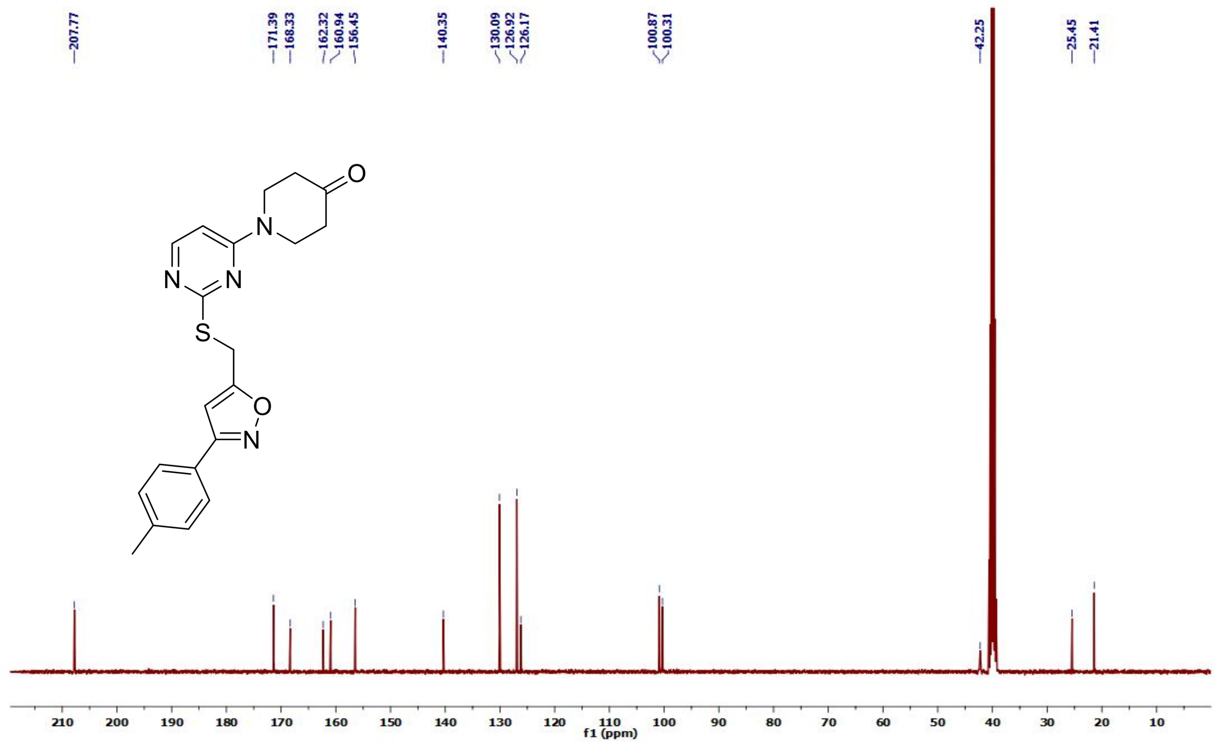

^{13}C NMR of 6l

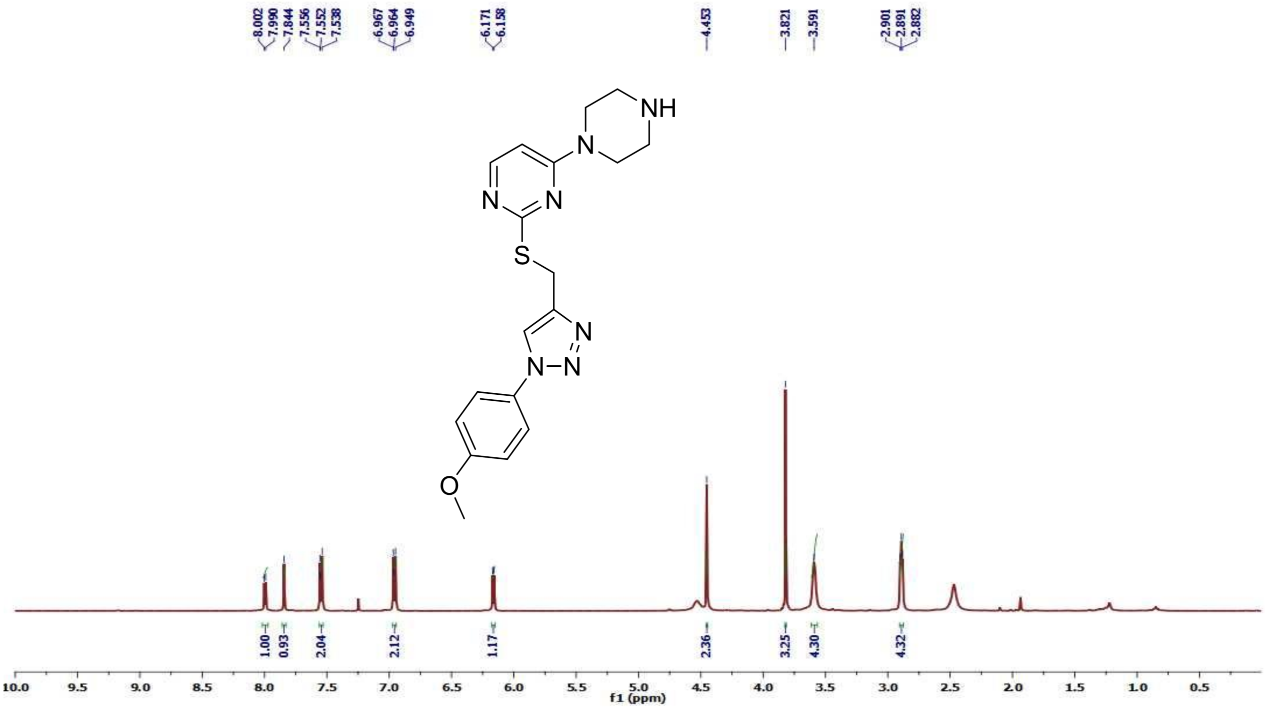

^{1}H NMR of 7a

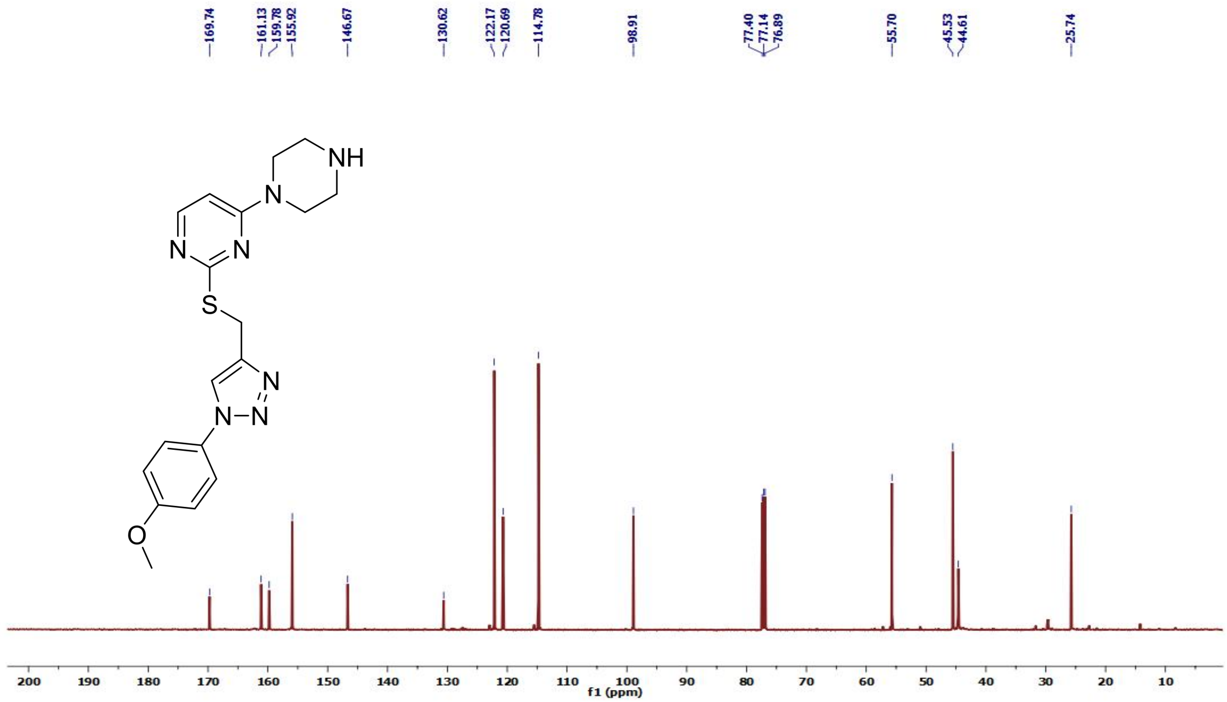

^{13}C NMR of 7a

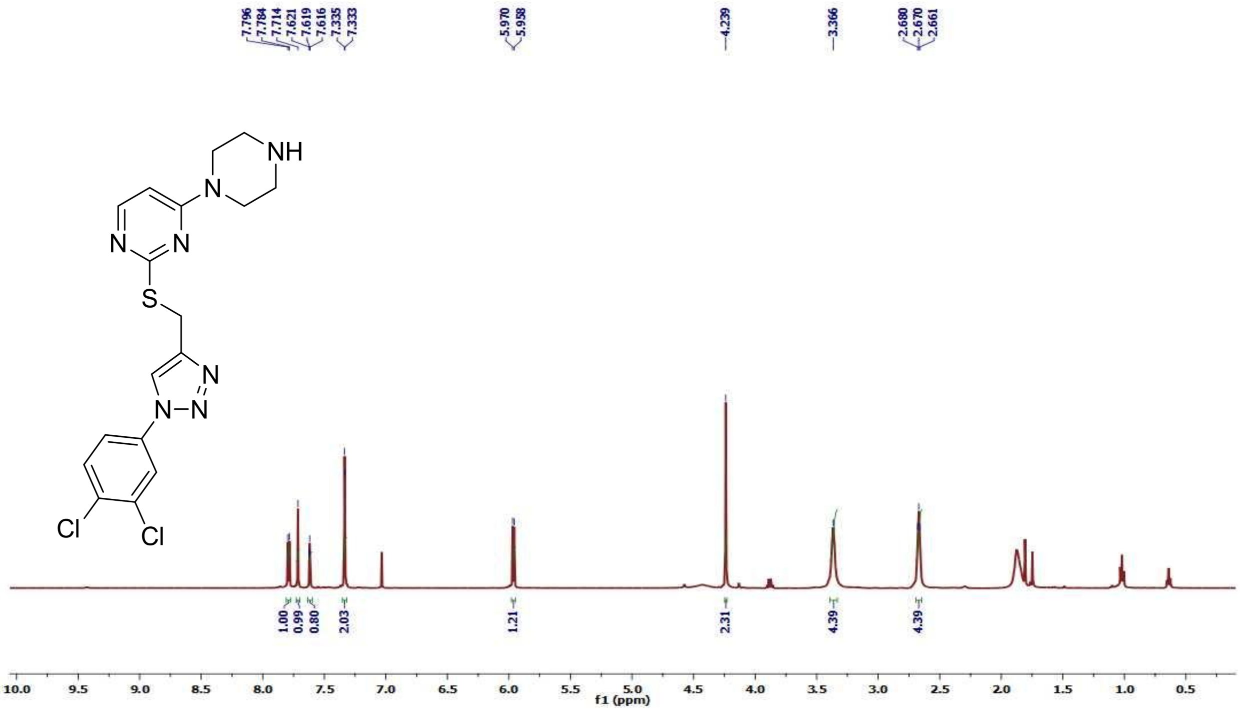

^{1}H NMR of 7b

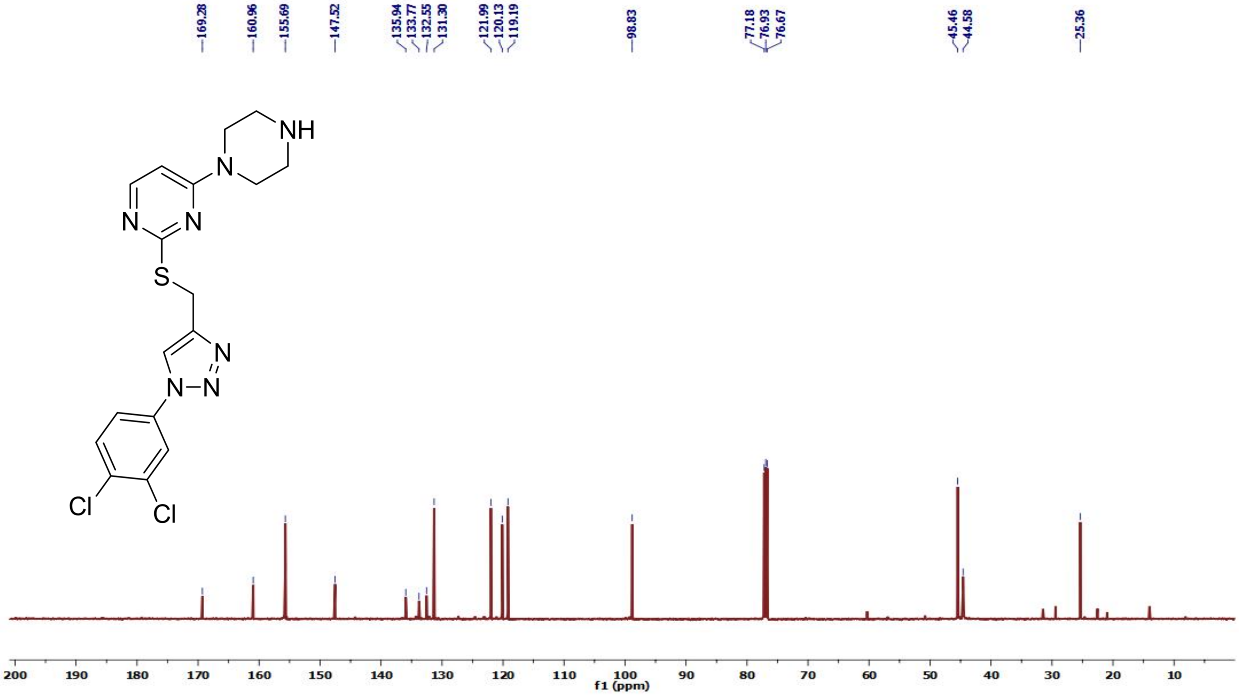

^{13}C NMR of 7b

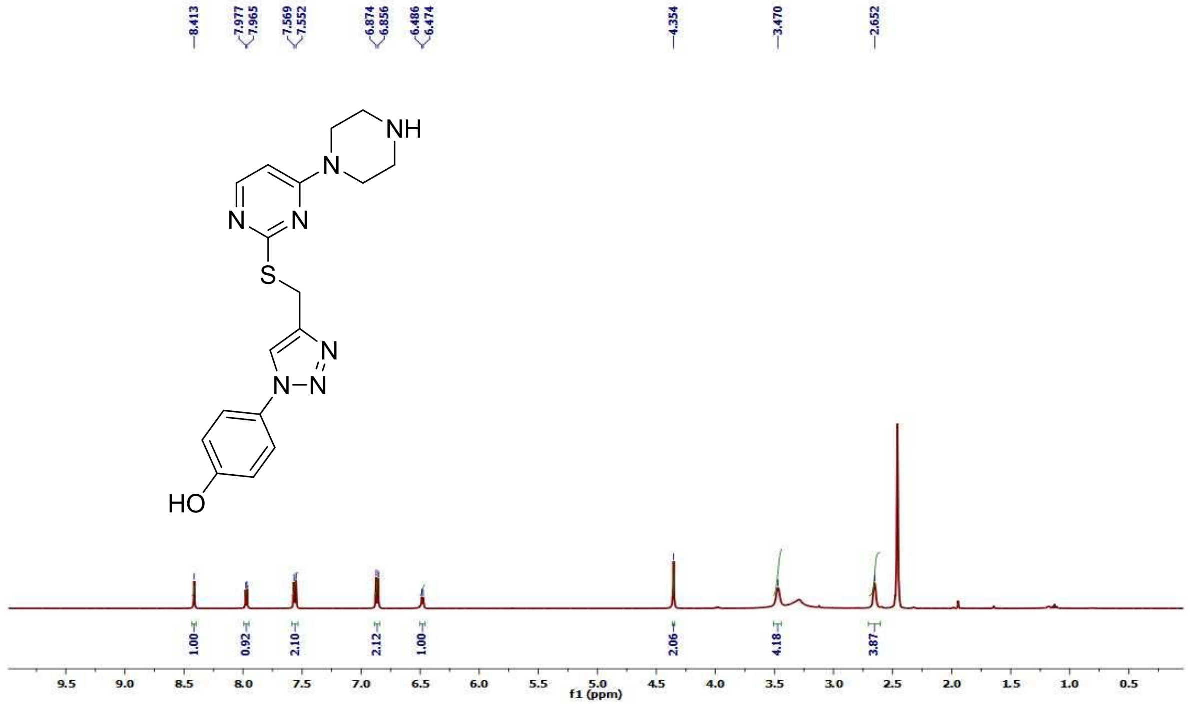

^{1}H NMR of 7c

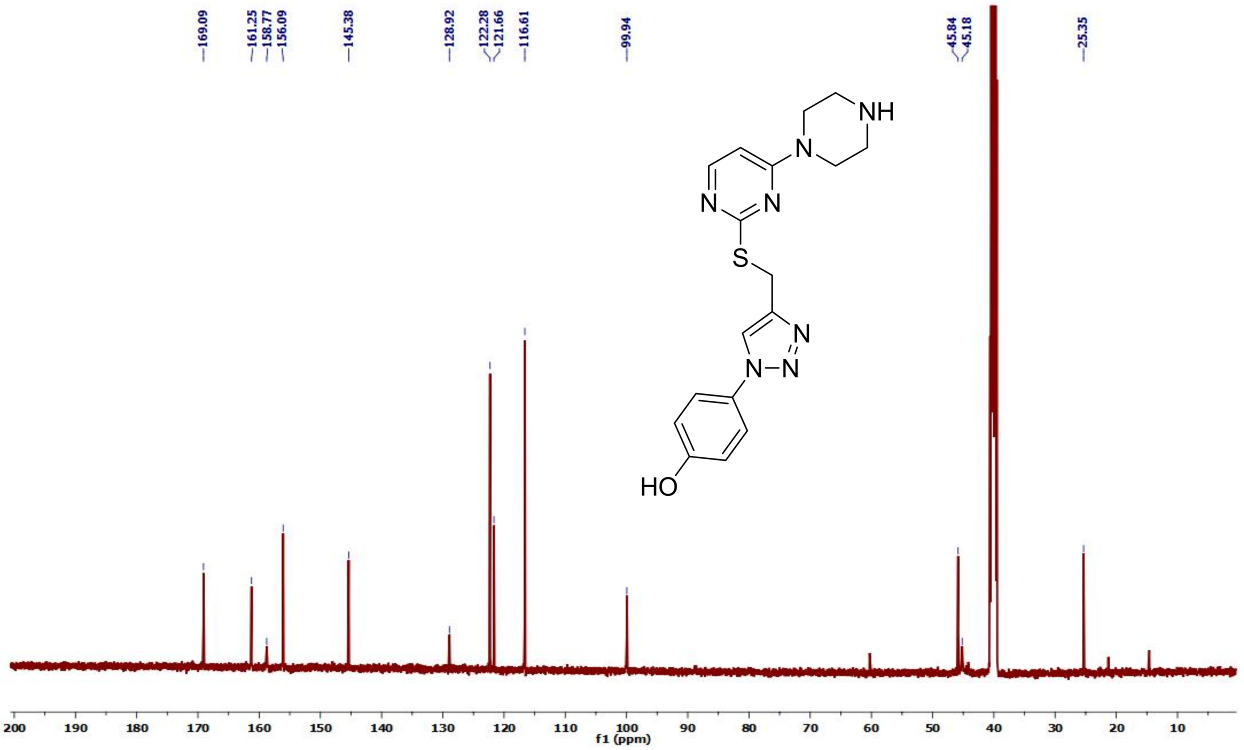

^{13}C NMR of 7c

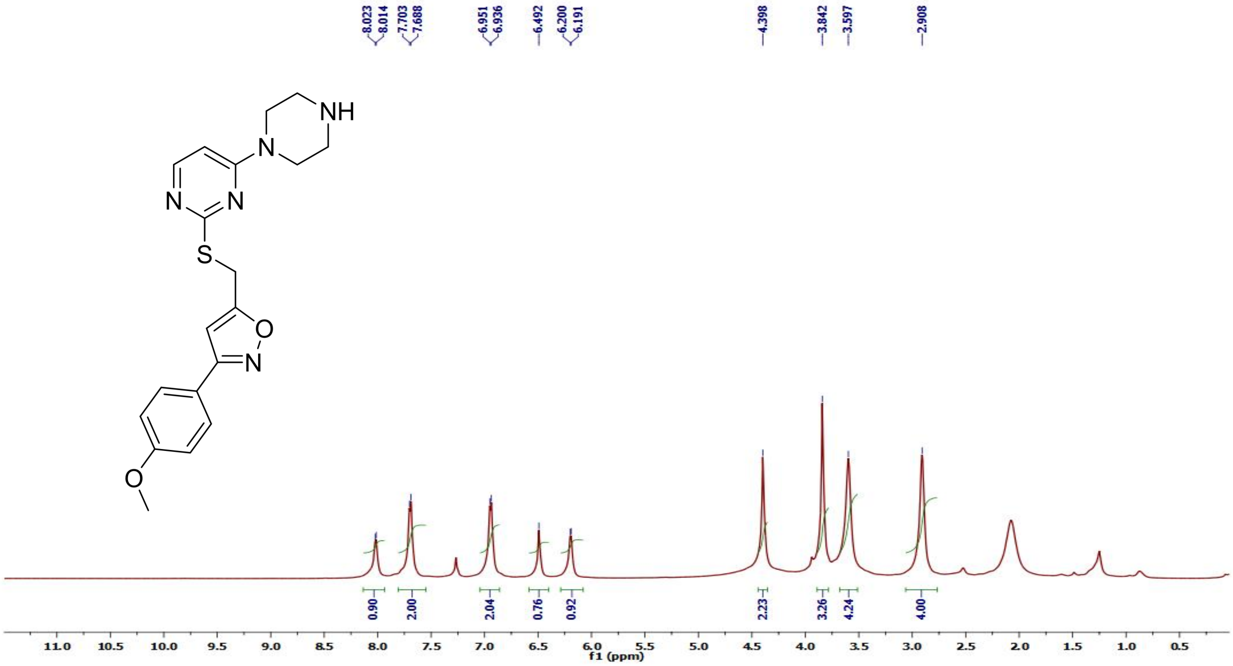

^{1}H NMR of 7d

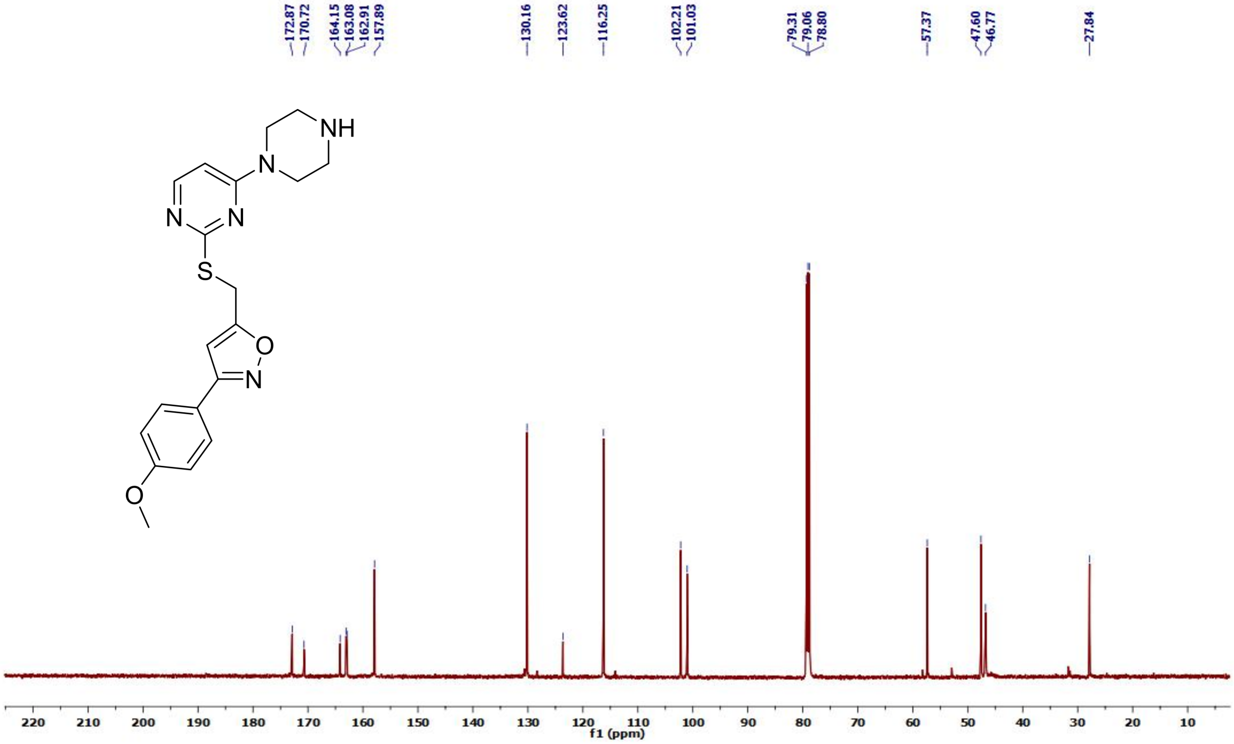

^{13}C NMR of 7d

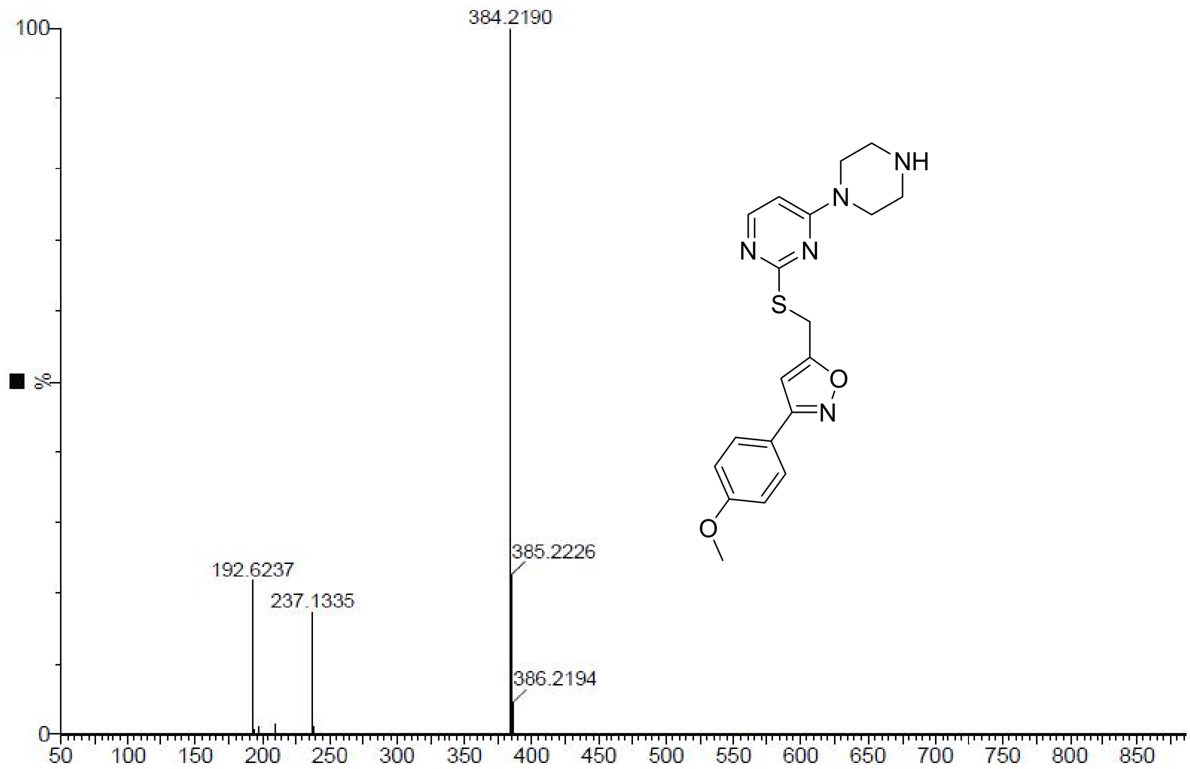

Mass spectrum of 7d

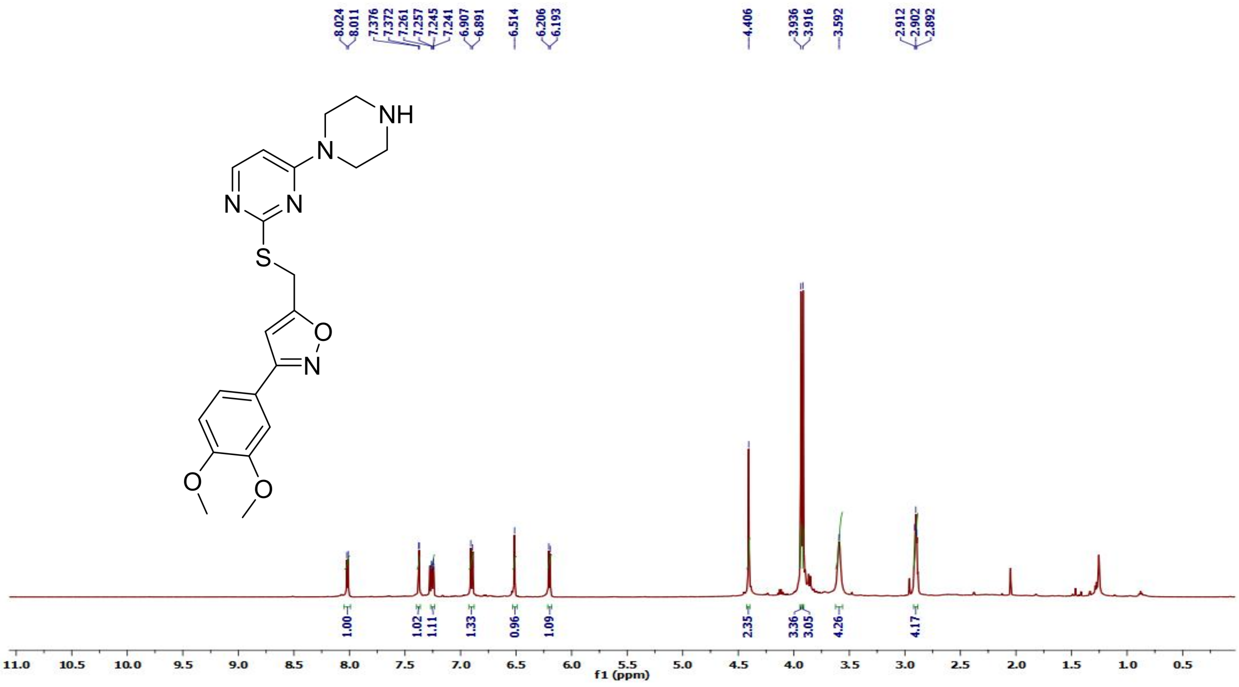

^{1}H NMR of 7e

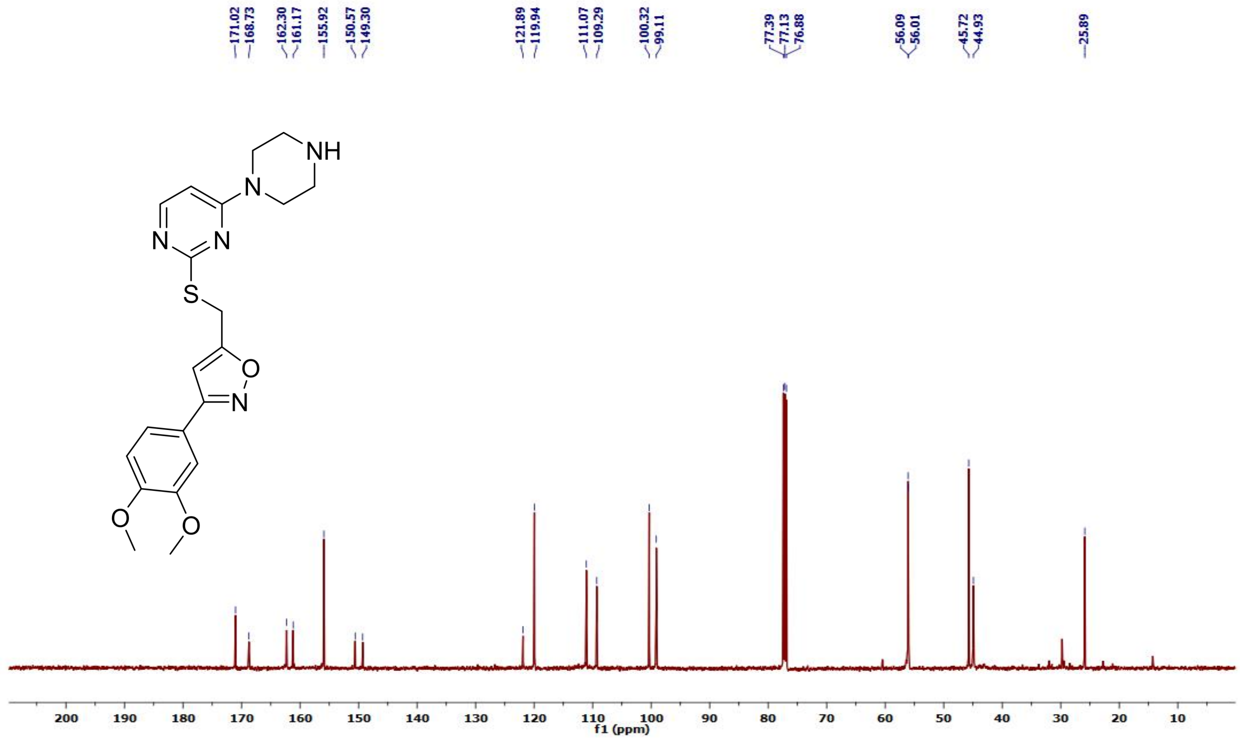

^{13}C NMR of 7e

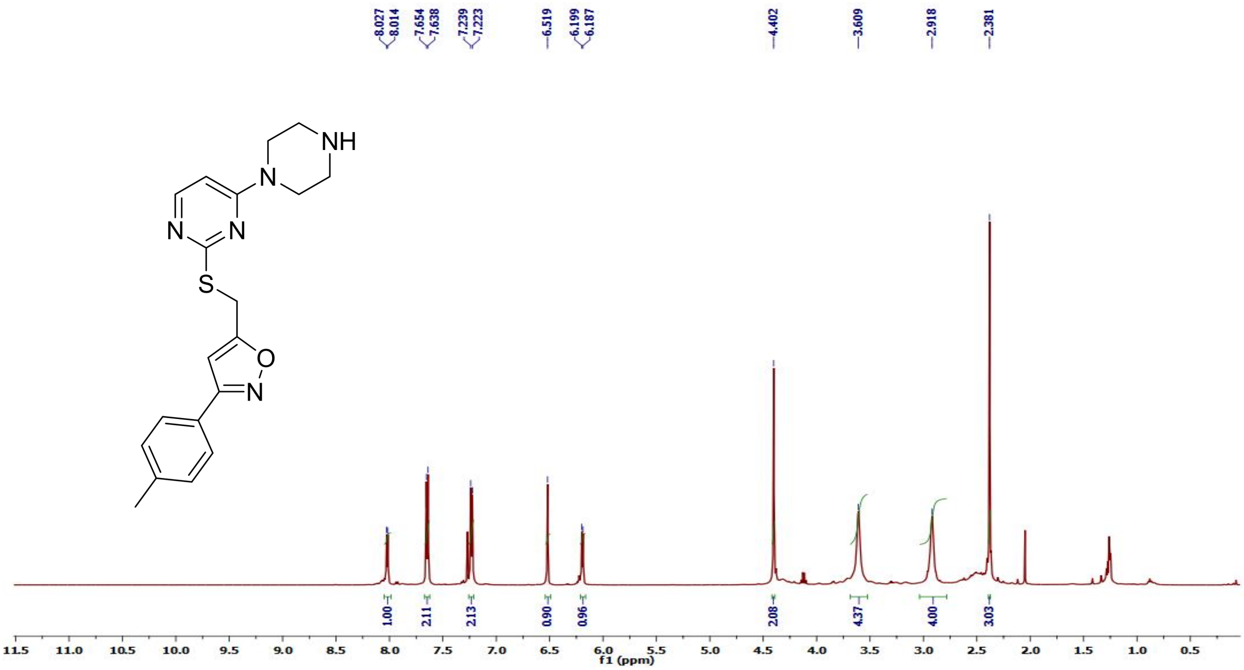

^{1}H NMR of 7f

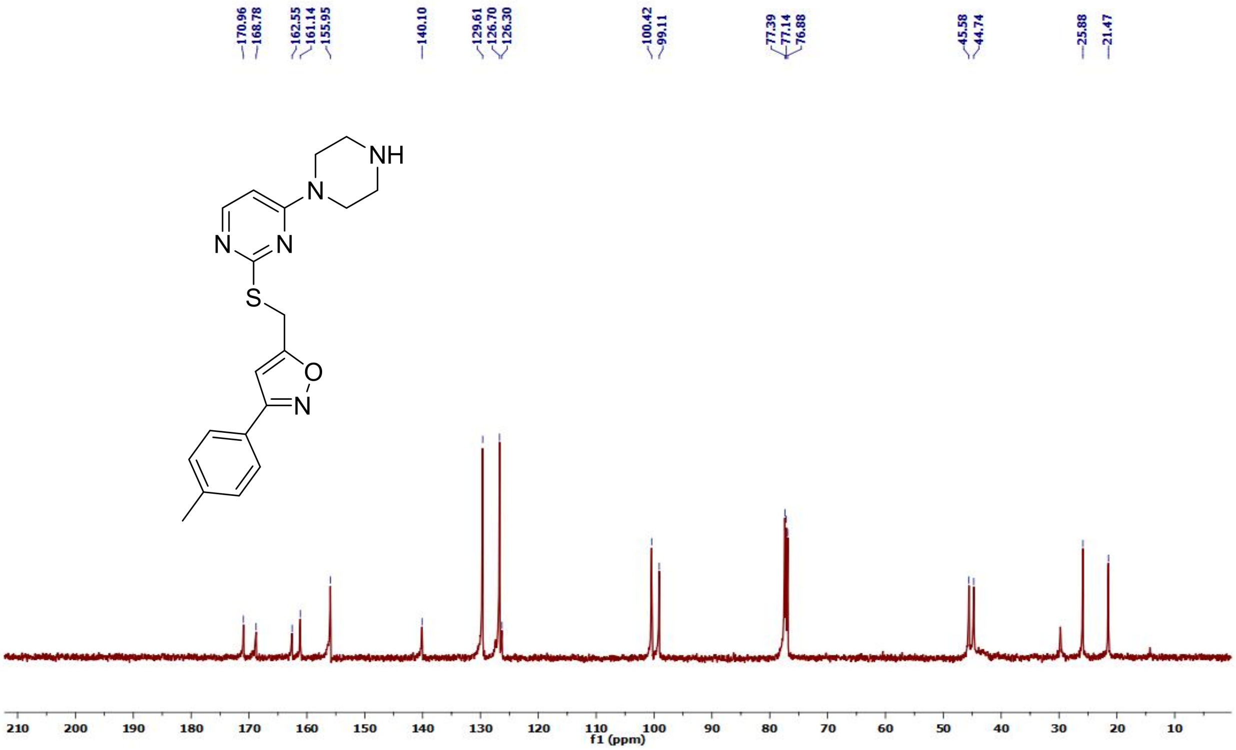

^{13}C NMR of 7f

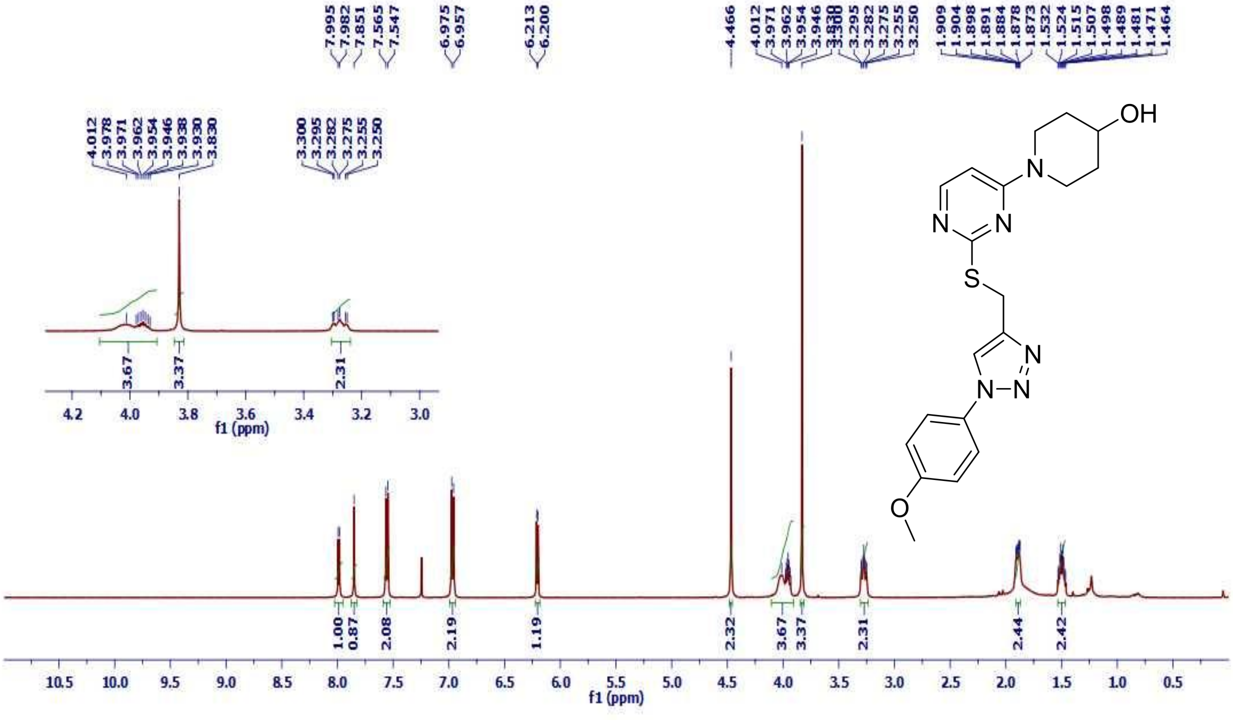

^{1}H NMR of 8a

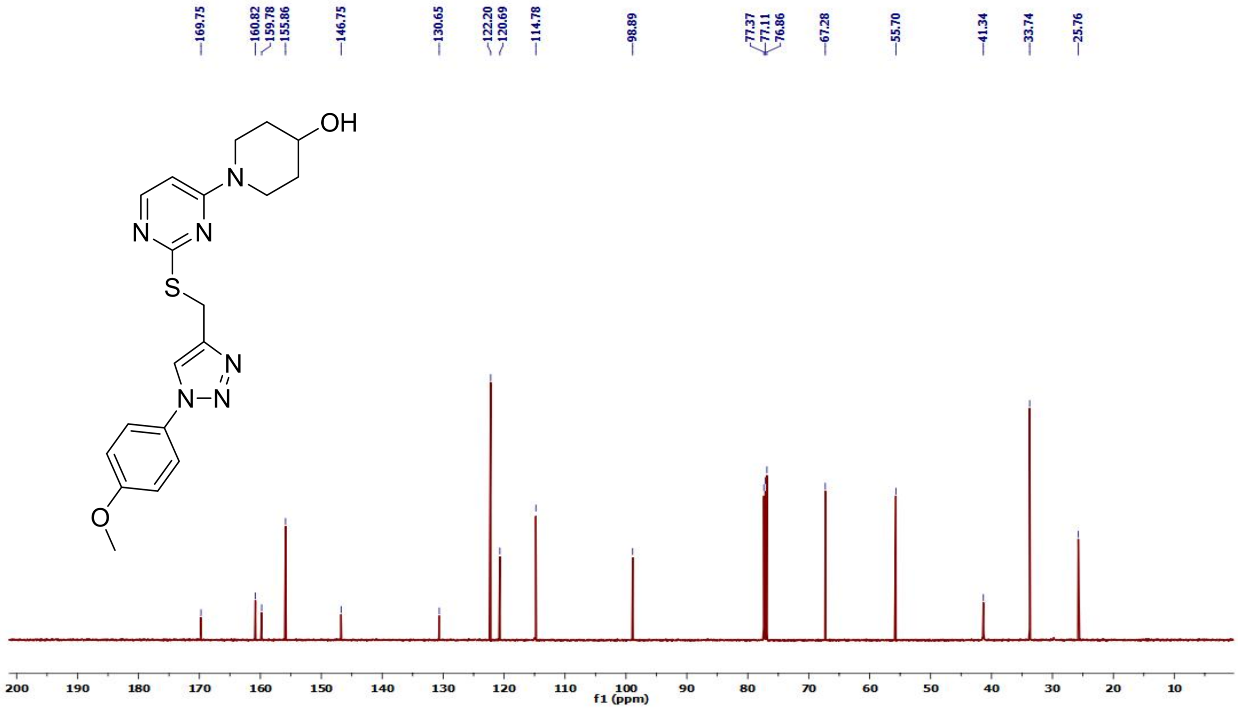

^{13}C NMR of 8a

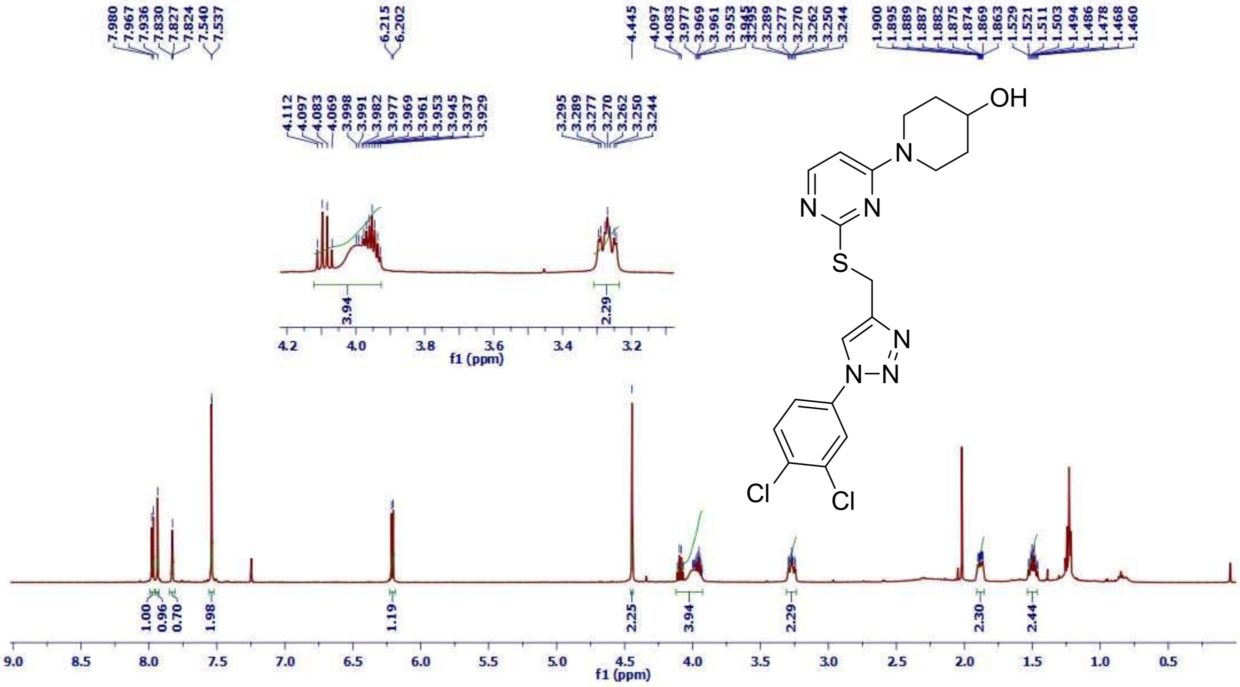

^{1}H NMR of 8b

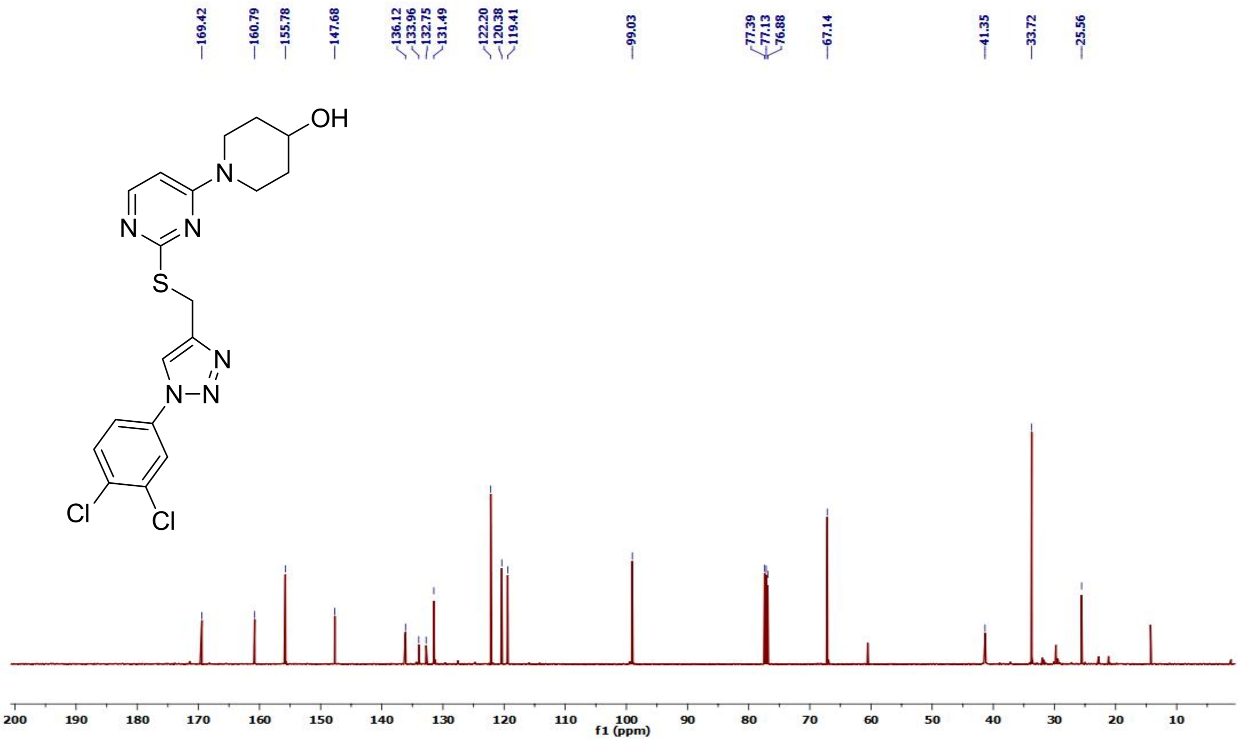

^{13}C NMR of 8b

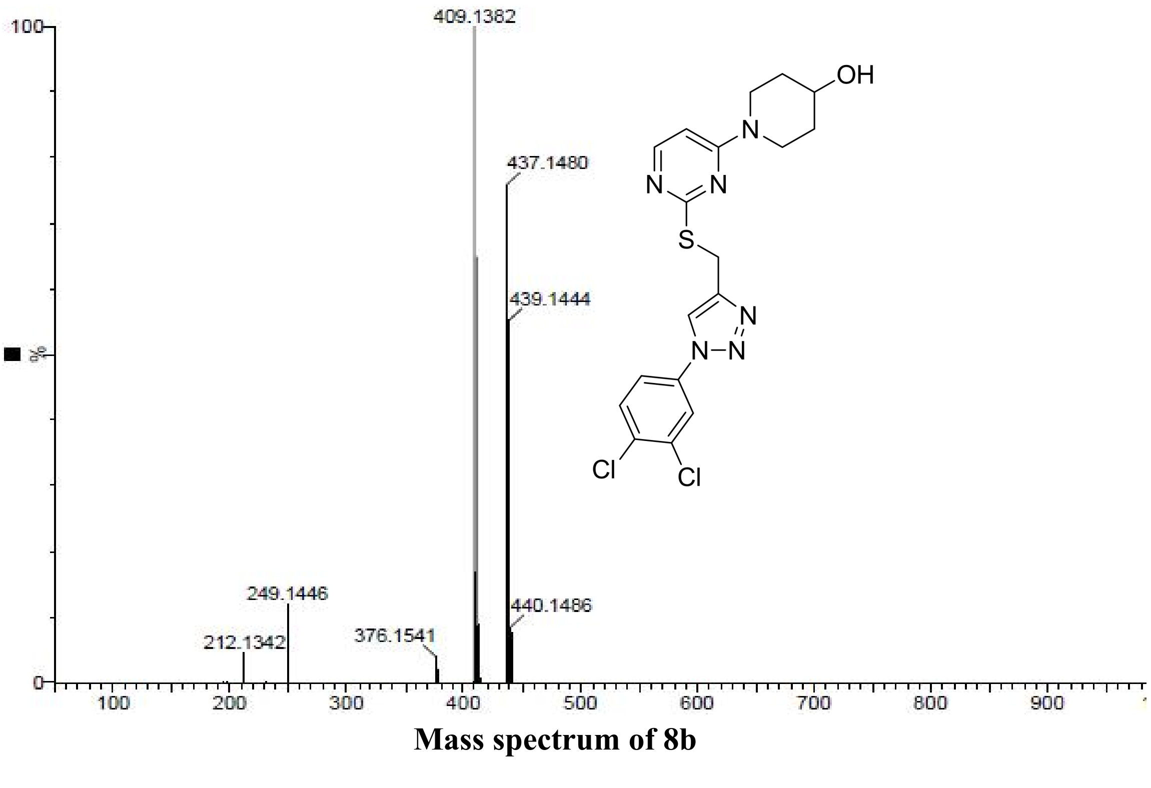

Mass spectrum of 8b

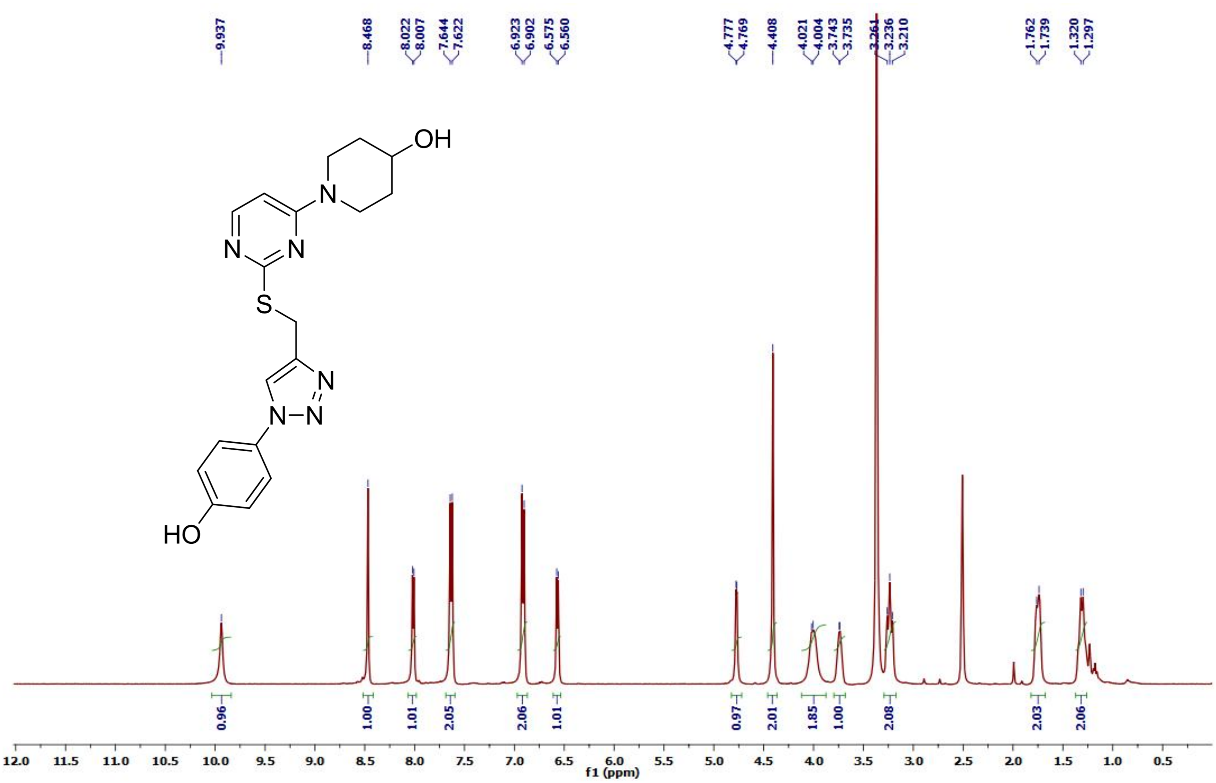

^{1}H NMR of 8c

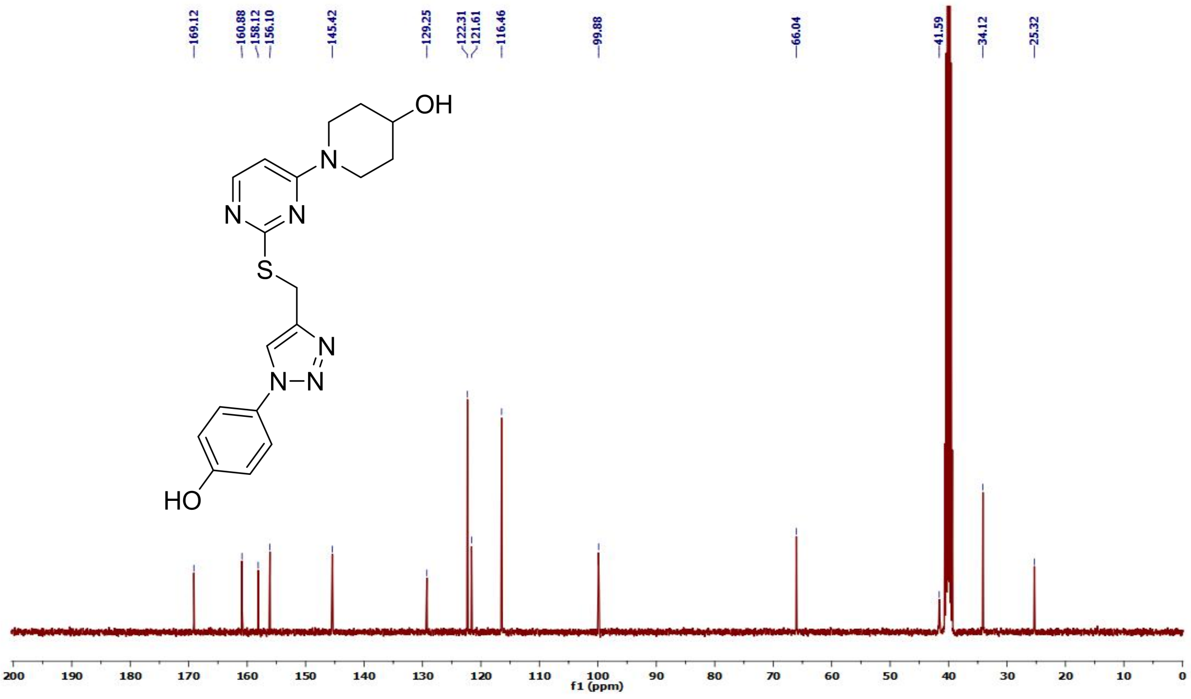

^{13}C NMR of 8c

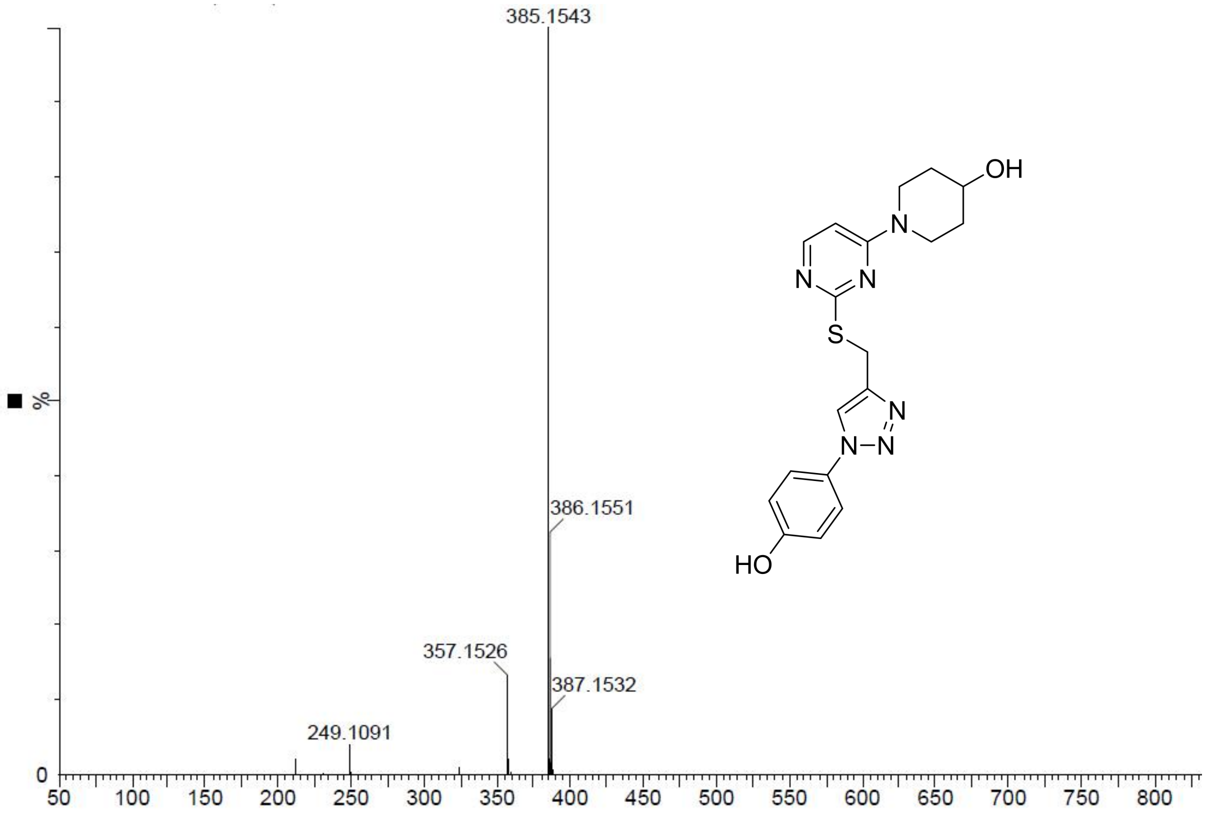

Mass spectrum of 8c

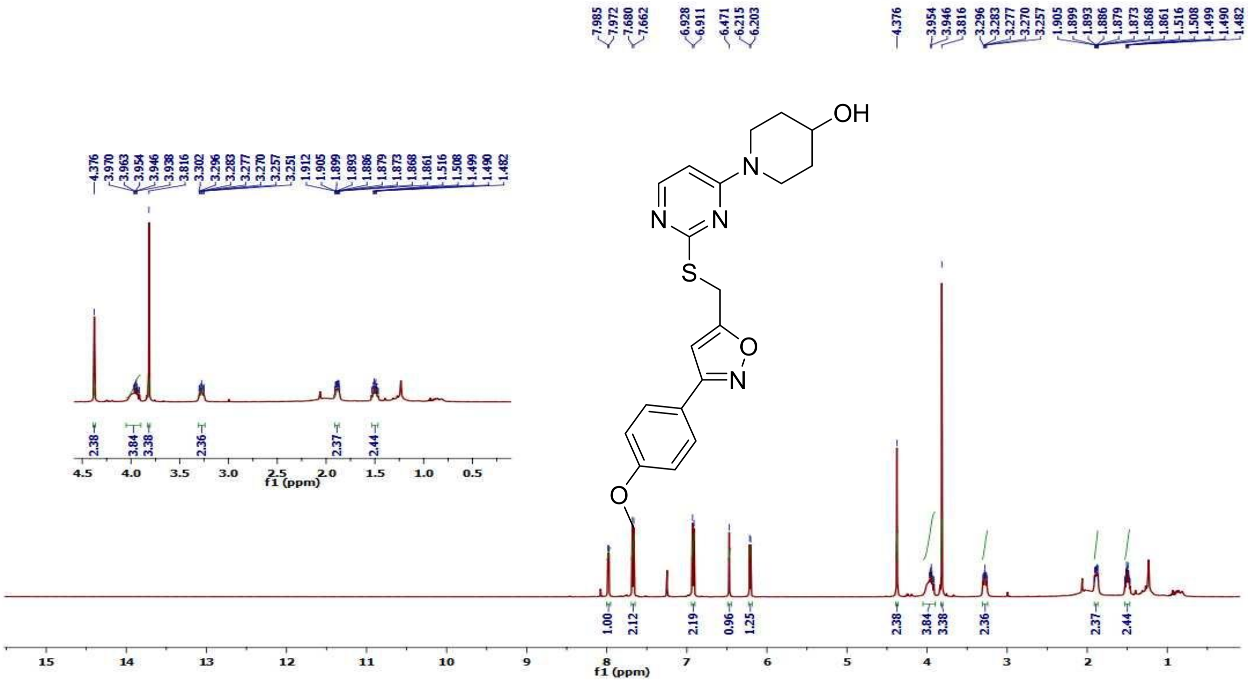

^{1}H NMR of 8d

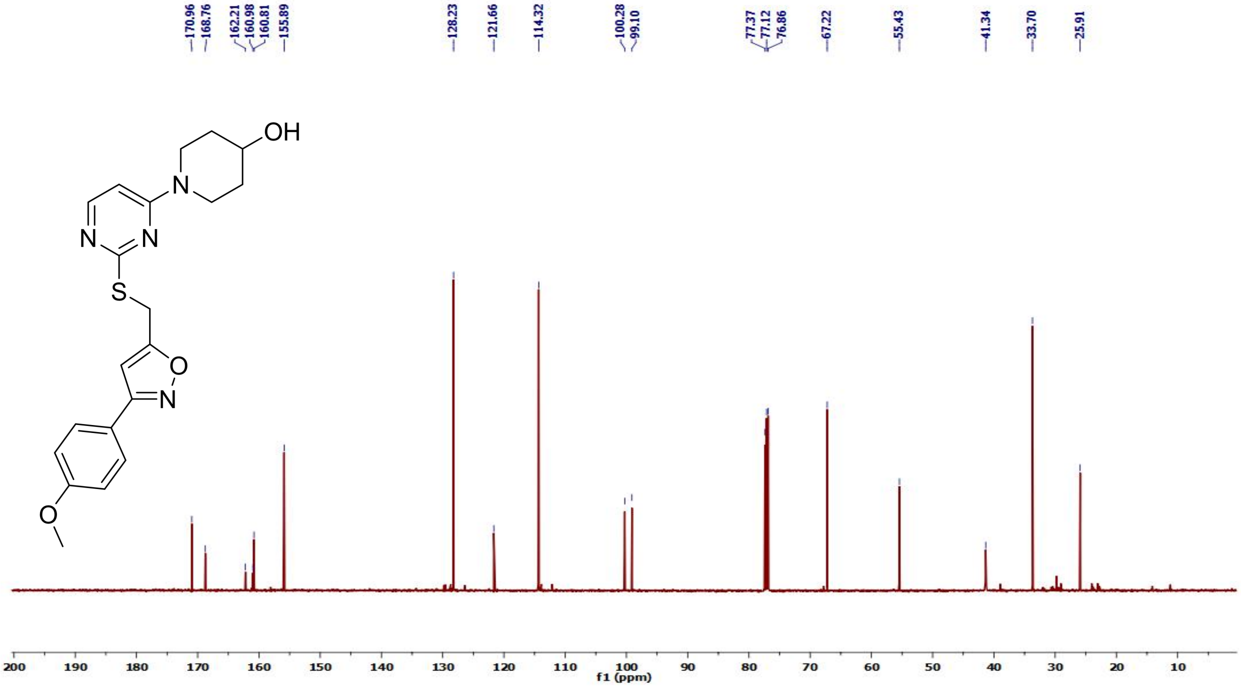

^{13}C NMR of 8d

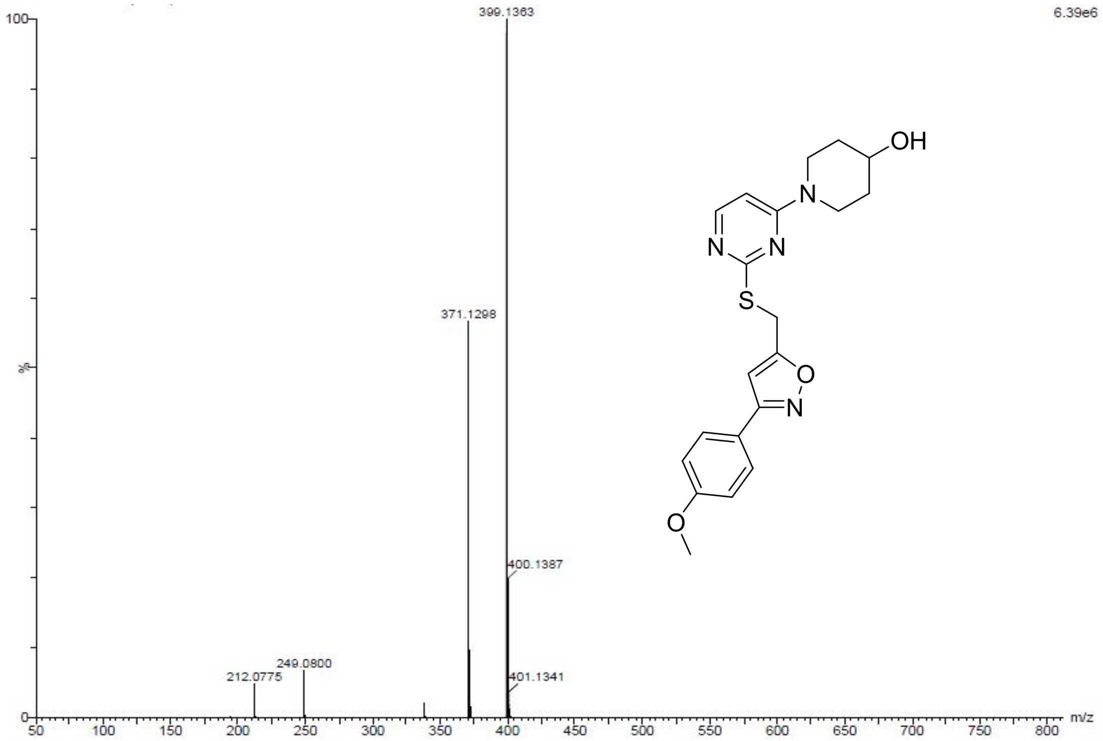

Mass spectrum of 8d

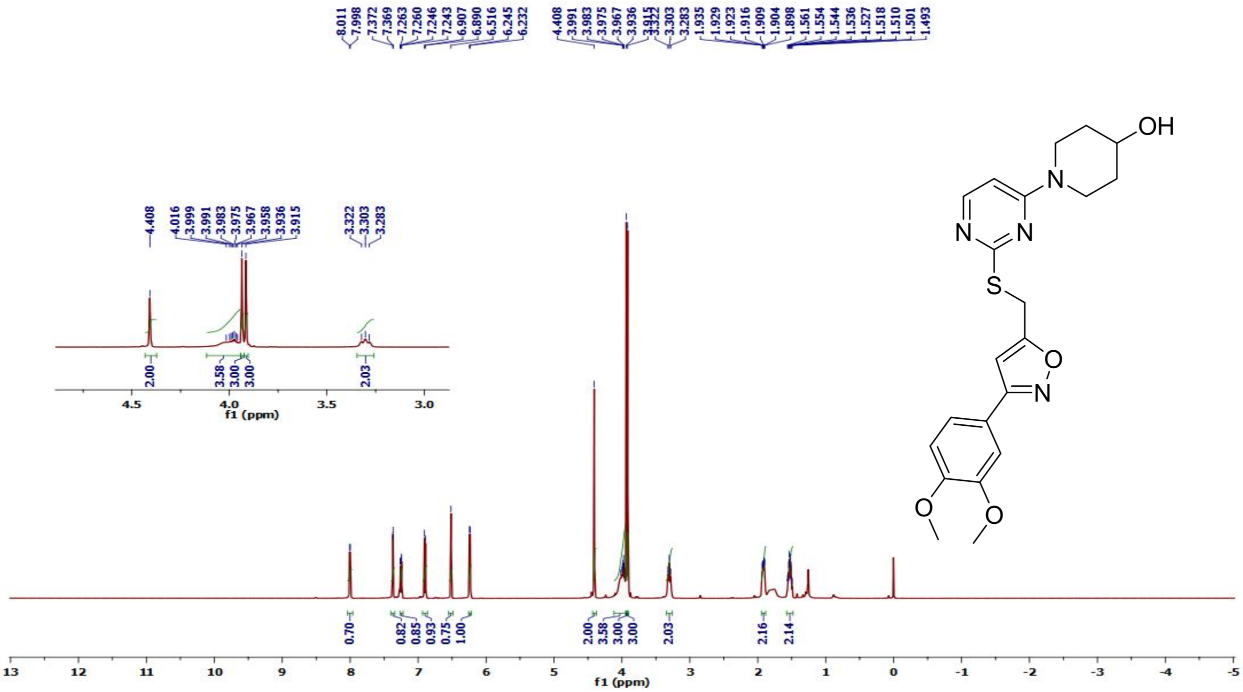

^{1}H NMR of 8e

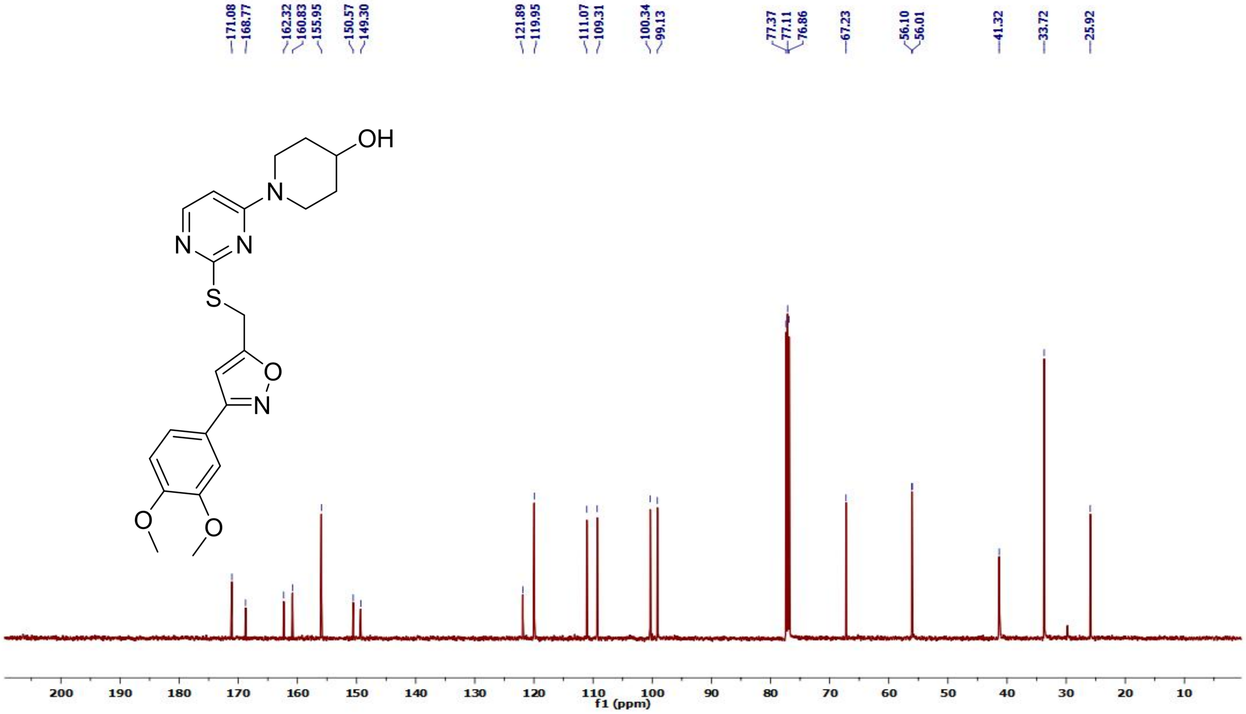

^{13}C NMR of 8e

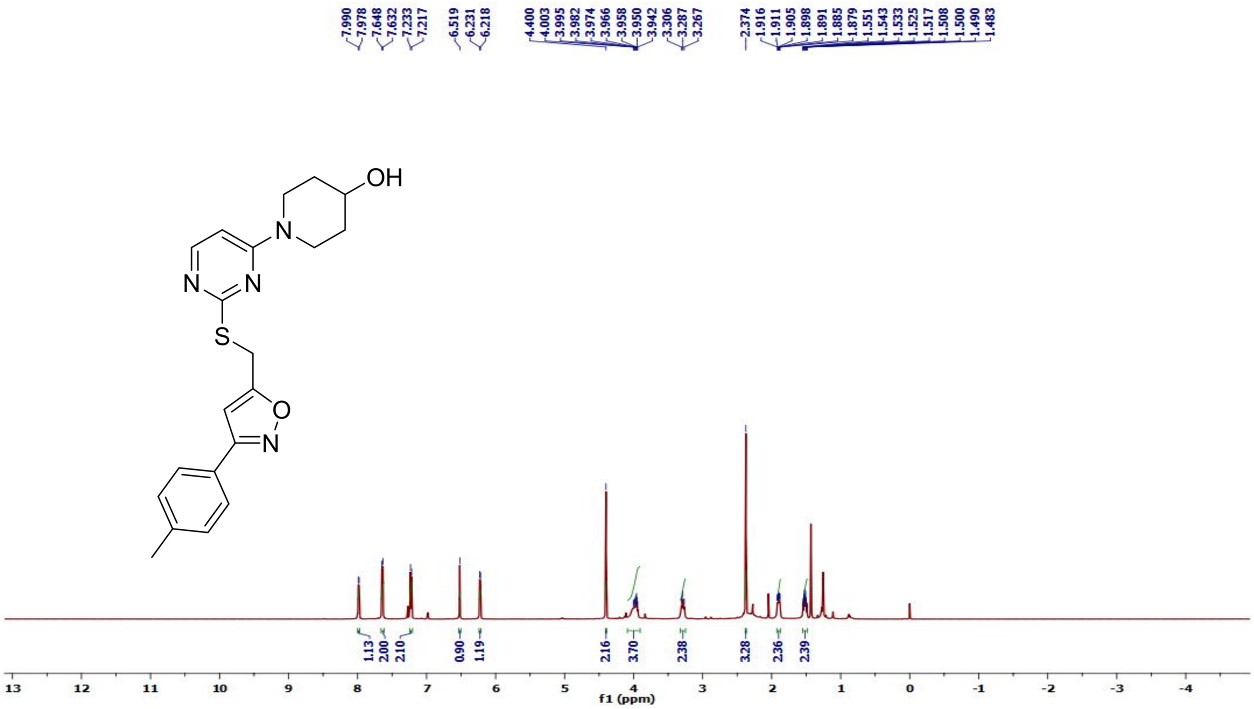

^{1}H NMR of 8f

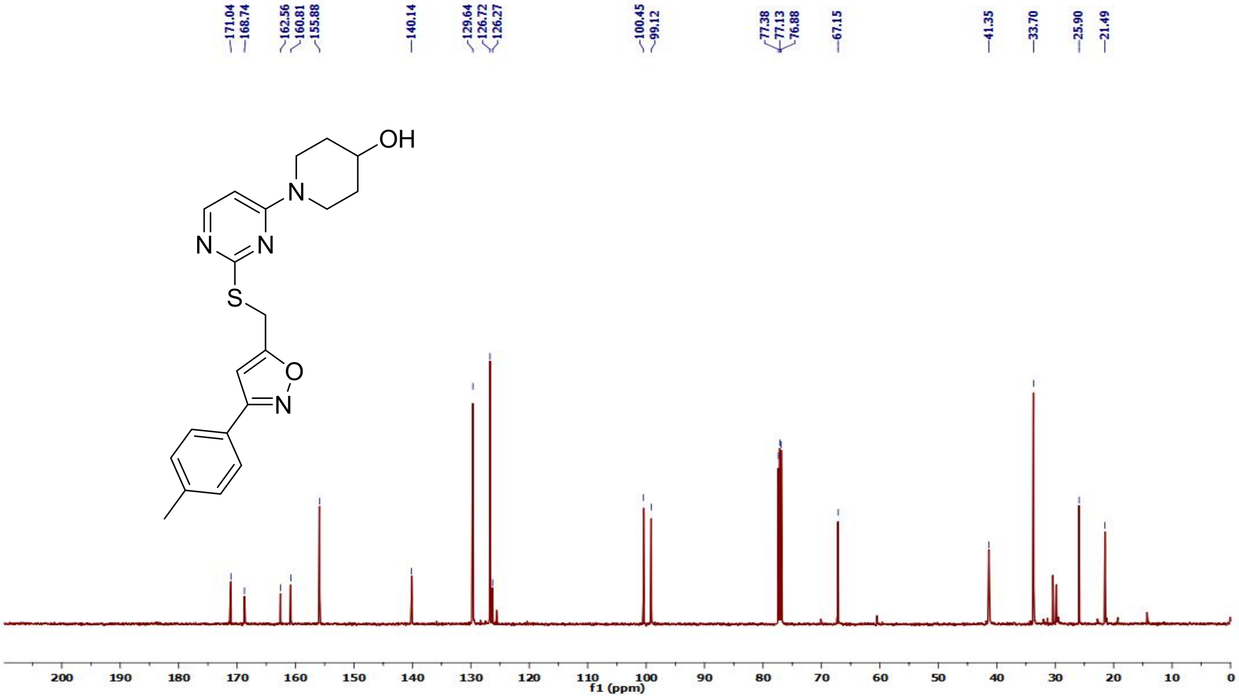

^{13}C NMR of 8f

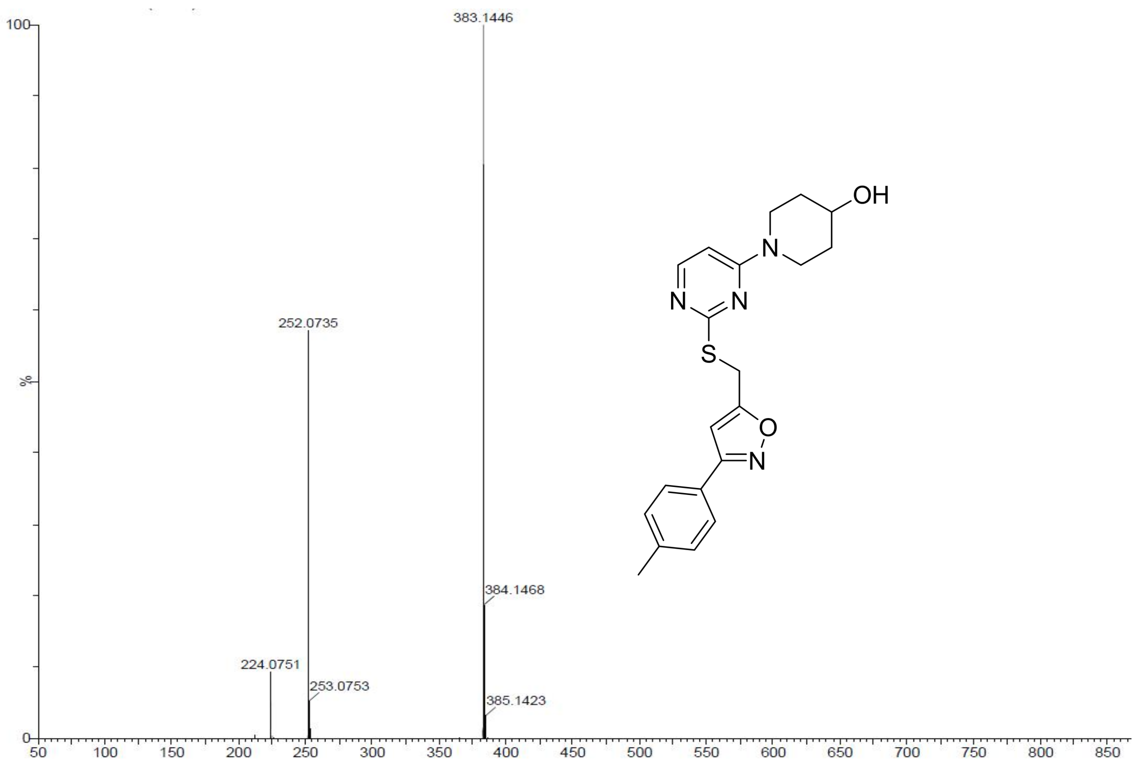

Mass spectrum of 8f

Log curves of synthesized compounds on MCF-7 cells

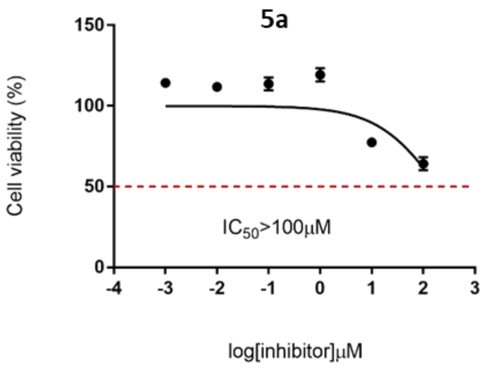

Conc.(μM)	Viability (%)	
	AVE.	±SD
0	100.0	2.5
0.01	111.8	0.8
0.1	113.6	4.0
1	119.4	4.1
10	77.5	1.4
100	64.2	4.1

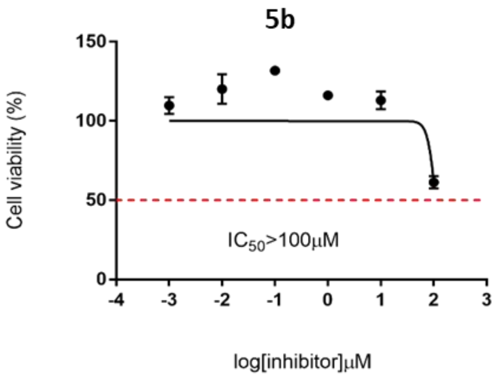

Conc.(μM)	Viability (%)	
	AVE.	±SD
0	100.0	5.3
0.01	120.2	9.2
0.1	131.8	0.2
1	116.2	1.3
10	113.0	5.7
100	61.3	3.7

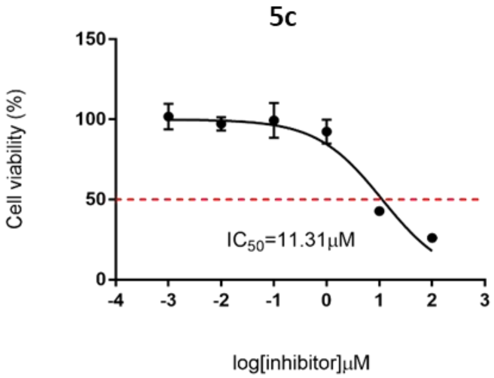

Conc.(μM)	Viability (%)	
	AVE.	±SD
0	100.0	8.0
0.01	97.3	4.2
0.1	99.4	10.9
1	92.4	7.5
10	42.8	2.1
100	26.1	2.6

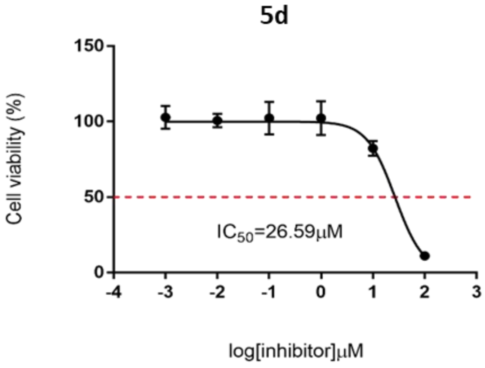

Conc.(μM)	Viability (%)	
	AVE.	±SD
0	100.0	7.5
0.01	100.7	4.4
0.1	102.4	10.9
1	102.4	11.2
10	82.3	4.9
100	11.0	1.4

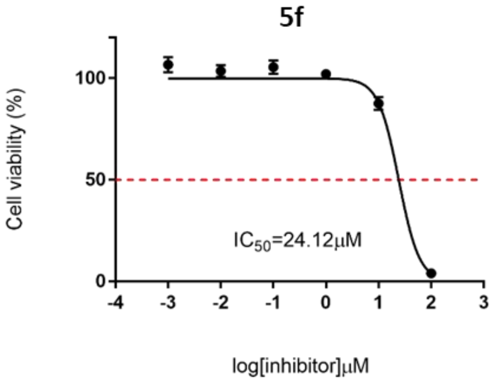

Conc.(μM)	Viability (%)	
	AVE.	±SD
0	100.0	3.8
0.01	103.6	2.9
0.1	105.6	3.3
1	102.2	1.2
10	87.7	3.2
100	4.1	0.9

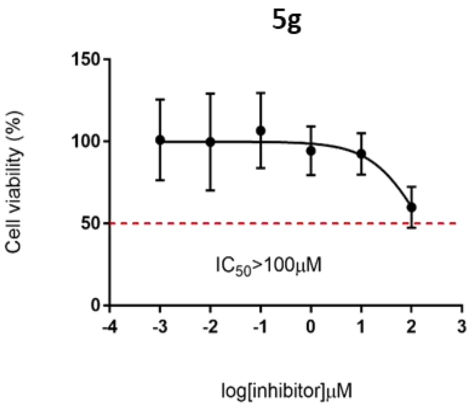

Conc.(μM)	Viability (%)	
	AVE.	±SD
0	100.0	24.7
0.01	99.8	29.5
0.1	106.7	22.8
1	94.4	14.9
10	92.5	12.6
100	60.0	12.5

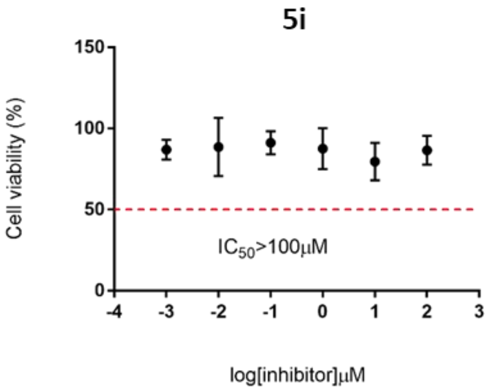

Conc.(μM)	Viability (%)	
	AVE.	±SD
0	100.0	6.2
0.01	88.5	18.0
0.1	91.2	7.2
1	87.5	12.7
10	79.6	11.5
100	86.6	8.9

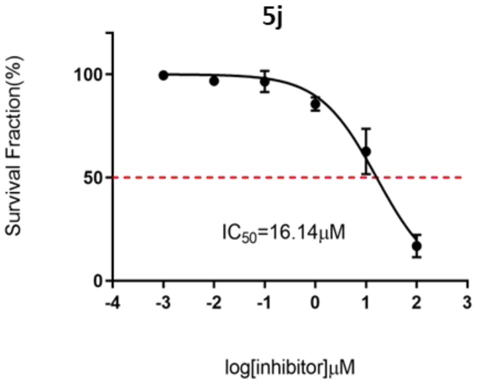

Conc.(μM)	Viability	
	AVE.	±SD
0	100.00	0.68
0.01	96.88	1.51
0.1	96.57	5.14
1	85.70	3.22
10	62.63	11.00
100	16.82	5.43

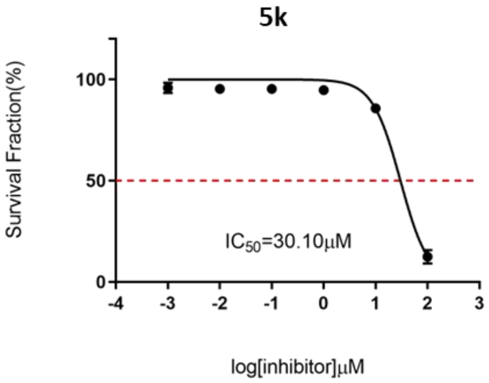

Conc.(μM)	Viability	
	AVE.	±SD
0	100.00	2.48
0.01	95.36	1.08
0.1	95.32	2.32
1	94.80	2.21
10	85.77	2.25
100	12.40	3.38

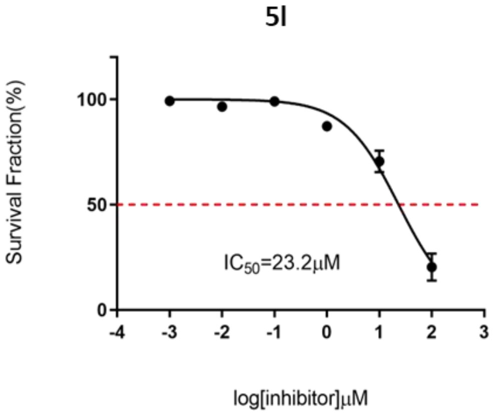

Conc.(μM)	Viability	
	AVE.	±SD
0	100.00	0.64
0.01	96.60	1.56
0.1	99.00	1.06
1	87.20	2.16
10	70.57	5.11
100	20.33	6.40

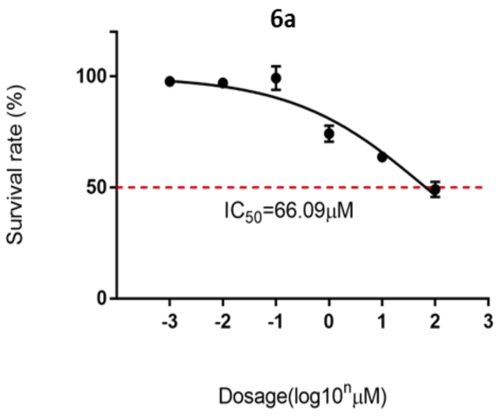

Conc.(μM)	Viability	
	AVE.	±SD
0	100.00	2.01
0.01	97.00	1.17
0.1	99.28	5.36
1	74.21	3.65
10	63.73	0.37
100	49.14	3.41

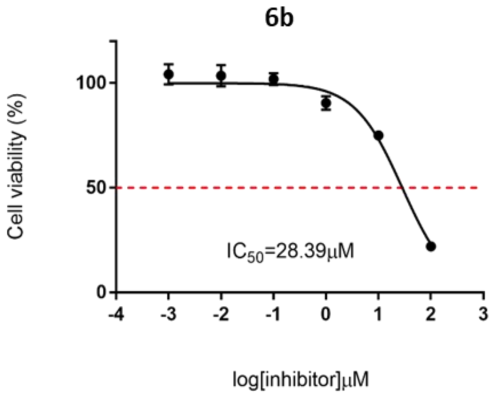

Conc.(μM)	Viability (%)	
	AVE.	±SD
0	100.0	4.8
0.01	103.5	5.2
0.1	102.0	2.9
1	90.5	3.2
10	75.0	1.5
100	22.0	1.4

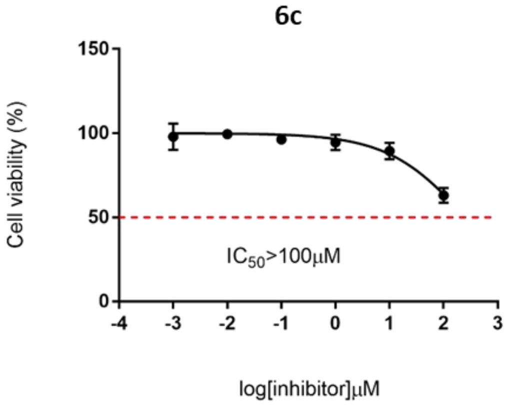

Conc.(μM)	Viability (%)	
	AVE.	±SD
0	100.0	7.7
0.01	99.5	1.9
0.1	96.3	1.3
1	94.6	4.5
10	89.4	4.9
100	63.1	4.5

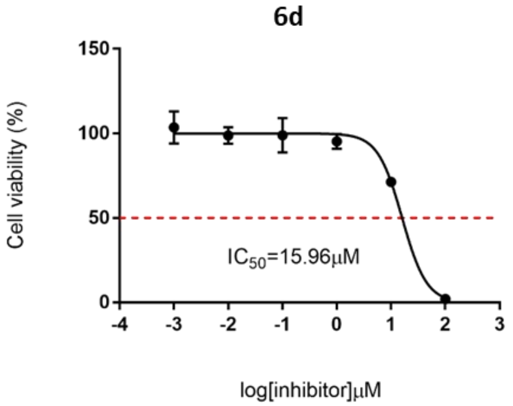

Conc.(μM)	Viability (%)	
	AVE.	±SD
0	100.0	9.5
0.01	98.8	4.9
0.1	98.9	10.1
1	95.3	4.4
10	71.4	0.5
100	2.1	0.5

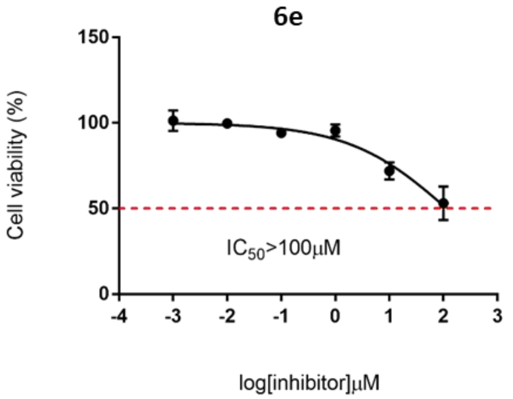

Conc.(μM)	Viability (%)	
	AVE.	±SD
0	100.0	6.0
0.01	99.7	2.6
0.1	94.1	1.0
1	95.6	3.5
10	72.0	5.1
100	53.1	9.7

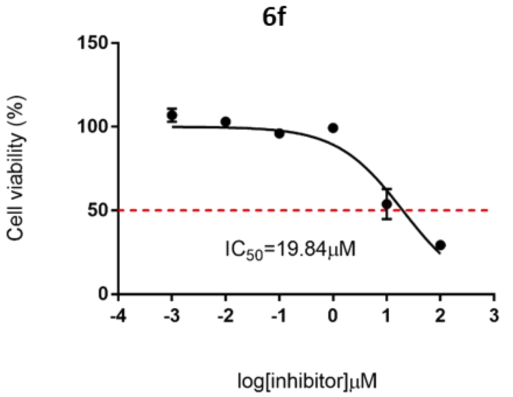

Conc.(μM)	Viability (%)	
	AVE.	±SD
0	100.0	4.0
0.01	103.1	2.8
0.1	96.1	2.7
1	99.3	2.0
10	53.8	9.0
100	29.3	1.9

6i

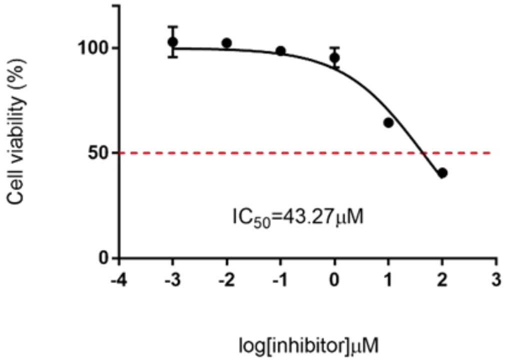

Conc.(μM)	Viability (%)	
	AVE.	±SD
0	100.0	7.2
0.01	102.4	1.5
0.1	98.7	1.0
1	95.5	4.8
10	64.6	1.0
100	40.7	0.8

6j

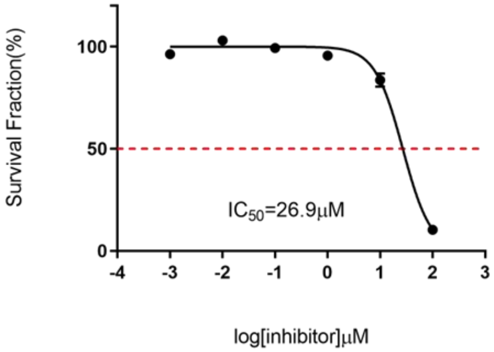

Conc.(μM)	Viability	
	AVE.	±SD
0	100.00	1.53
0.01	103.00	1.00
0.1	99.33	0.58
1	95.67	0.58
10	83.67	3.21
100	10.34	0.15

6k

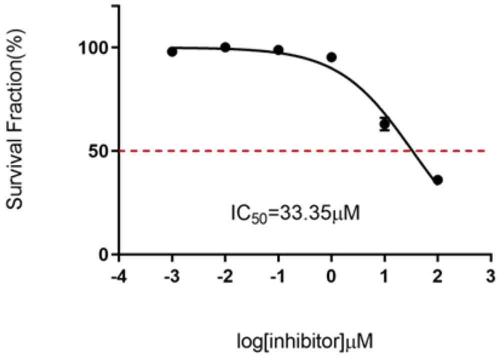

Conc.(μM)	Viability	
	AVE.	±SD
0	100.00	2.00
0.01	100.17	0.15
0.1	98.83	0.68
1	95.33	0.58
10	63.00	3.00
100	36.00	2.00

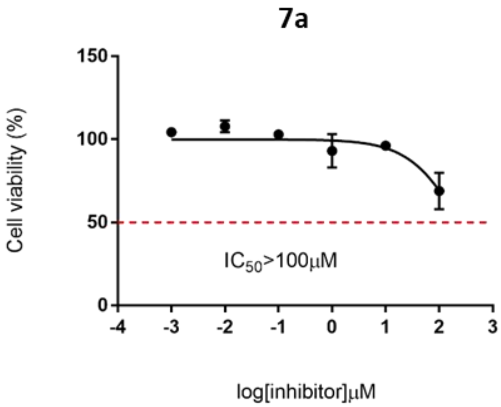

Conc.(μM)	Viability (%)	
	AVE.	±SD
0	100.0	2.4
0.01	107.9	3.6
0.1	103.0	1.7
1	93.0	10.0
10	96.2	1.5
100	68.9	11.0

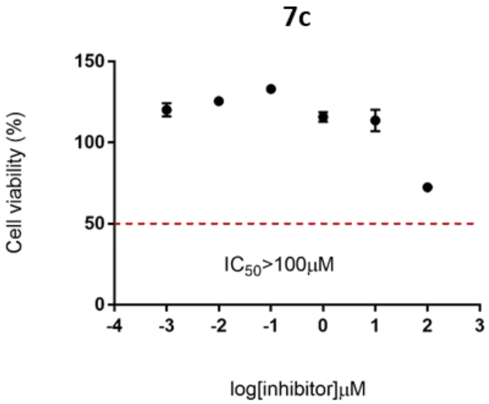

Conc.(μM)	Viability (%)	
	AVE.	±SD
0	100.0	4.0
0.01	125.6	1.1
0.1	133.1	1.2
1	115.9	3.0
10	113.7	6.7
100	72.4	2.5

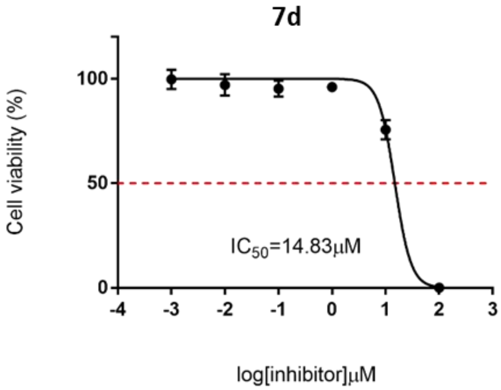

Conc.(μM)	Viability (%)	
	AVE.	±SD
0	100.0	4.7
0.01	97.1	5.1
0.1	95.2	3.9
1	96.0	1.7
10	75.7	4.6
100	0.0	0.0

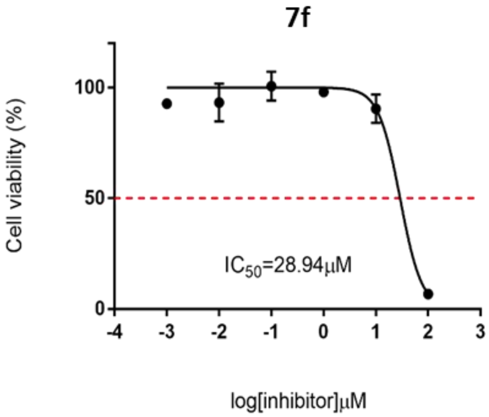

Conc.(μM)	Viability (%)	
	AVE.	±SD
0	100.0	2.2
0.01	93.3	8.5
0.1	100.6	6.5
1	98.1	1.9
10	90.4	6.4
100	6.8	1.0

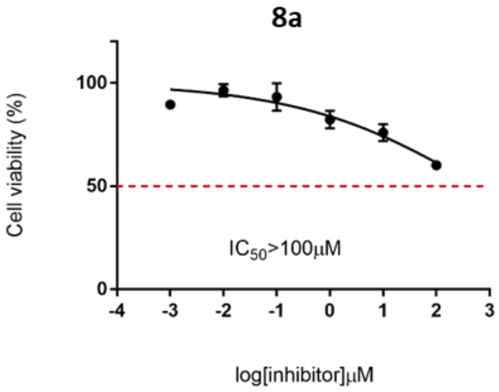

Conc.(μM)	Viability (%)	
	AVE.	±SD
0	100.0	1.3
0.01	96.5	3.0
0.1	93.2	6.6
1	82.3	4.2
10	76.0	4.2
100	60.2	1.8

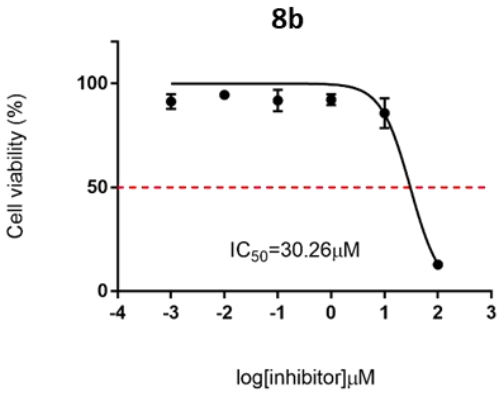

Conc.(μM)	Viability (%)	
	AVE.	±SD
0	100.0	3.5
0.01	94.6	0.8
0.1	91.9	5.1
1	92.2	2.6
10	85.7	7.2
100	12.8	0.8

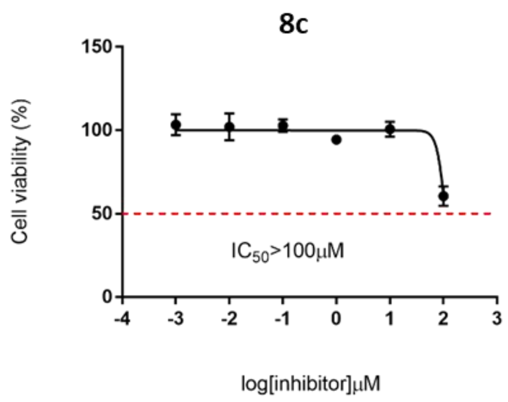

Conc.(μM)	Viability (%)	
	AVE.	±SD
0	100.0	6.3
0.01	102.1	8.1
0.1	102.8	3.8
1	94.4	2.9
10	100.7	4.5
100	60.6	5.8

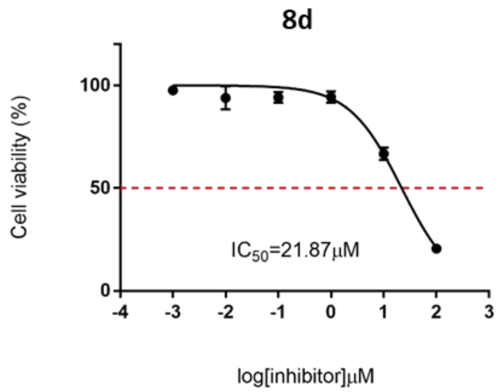

Conc.(μM)	Viability (%)	
	AVE.	±SD
0	100.0	2.3
0.01	93.9	5.7
0.1	94.1	2.6
1	94.4	2.7
10	66.8	3.0
100	20.5	2.3

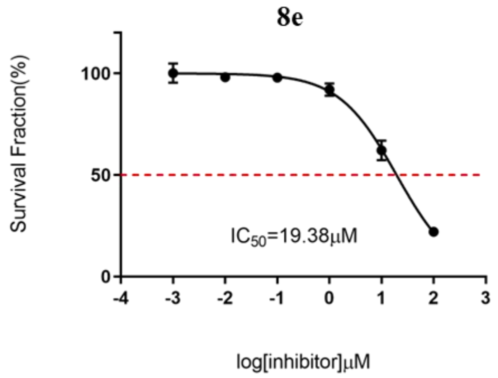

Conc.(μM)	Viability	
	AVE.	±SD
0	100.00	4.71
0.01	98.20	2.39
0.1	97.93	2.08
1	92.07	3.06
10	62.13	4.79
100	22.00	1.00

Log curves of active compounds on MCF-10A cells

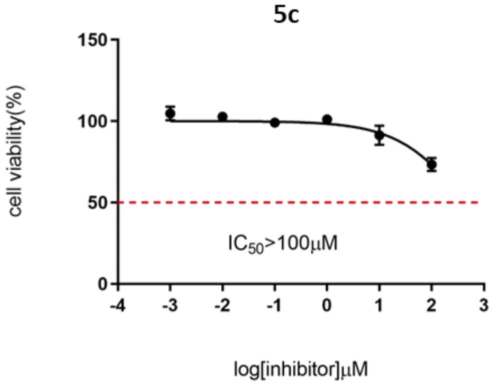

Conc.(μM)	Viability	
	AVE.	±SD
0	100.00	4.16
0.01	102.67	2.52
0.1	99.00	1.00
1	101.00	2.65
10	91.33	5.86
100	73.33	4.04

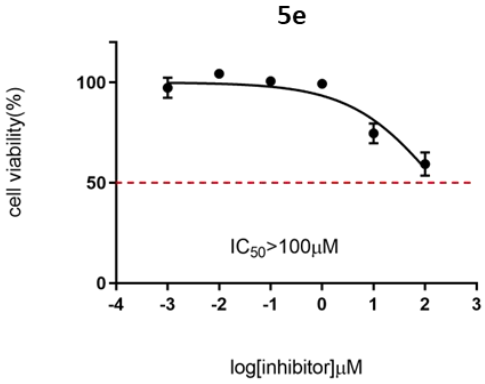

Conc.(μM)	Viability	
	AVE.	±SD
0	100.00	5.03
0.01	104.33	2.08
0.1	100.67	1.15
1	99.32	0.78
10	74.67	4.93
100	59.33	5.77

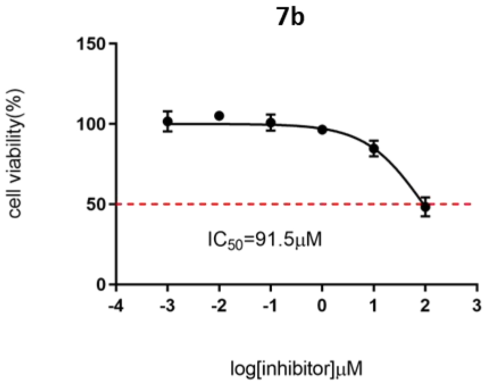

Conc.(μM)	Viability	
	AVE.	±SD
0	100.00	6.33
0.01	105.12	2.90
0.1	100.80	5.00
1	96.42	1.66
10	84.67	4.93
100	48.33	5.86

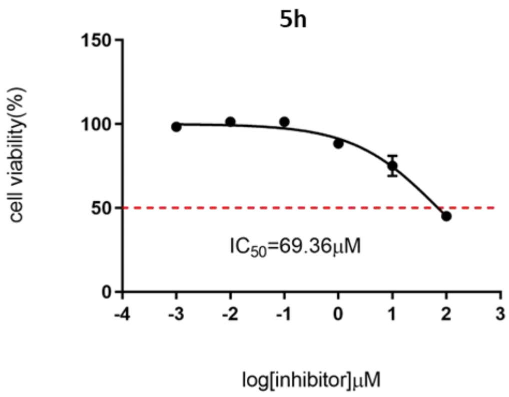

Conc.(μM)	Viability	
	AVE.	±SD
0	100.00	1.08
0.01	101.31	1.14
0.1	101.33	0.58
1	88.33	1.15
10	75.00	6.08
100	45.00	1.00

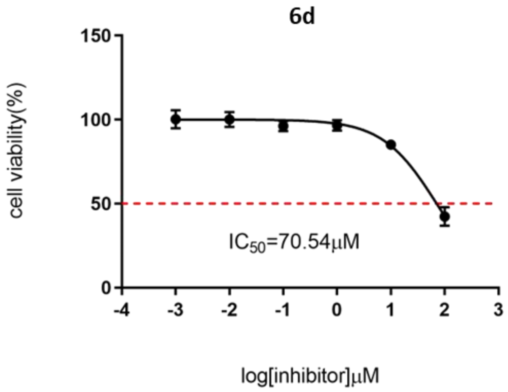

Conc.(μM)	Viability	
	AVE.	±SD
0	100.00	5.41
0.01	100.10	4.46
0.1	96.23	3.06
1	96.50	3.13
10	85.00	1.00
100	42.33	5.51

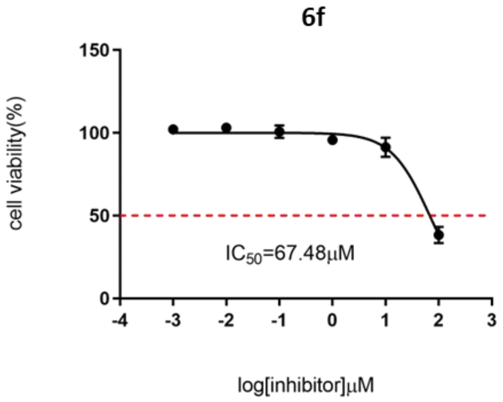

Conc.(μM)	Viability	
	AVE.	±SD
0	100.00	2.65
0.01	103.07	2.79
0.1	100.67	3.79
1	95.67	1.15
10	91.33	5.77
100	38.33	4.93

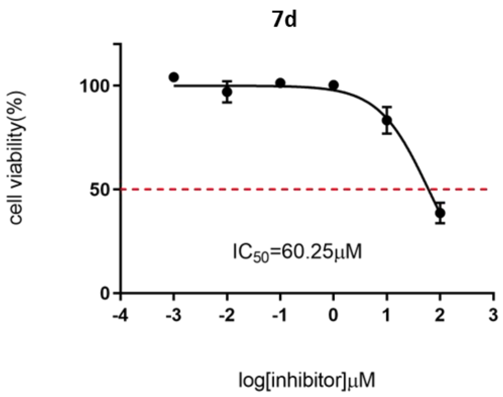

Conc.(μM)	Viability	
	AVE.	±SD
0	100.00	1.06
0.01	97.08	5.09
0.1	101.33	1.15
1	100.33	1.53
10	83.33	6.43
100	38.67	4.93

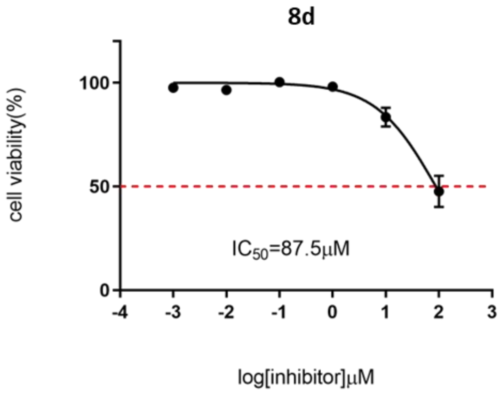

Conc.(μM)	Viability	
	AVE.	±SD
0	100.00	2.34
0.01	96.59	1.51
0.1	100.33	1.53
1	98.19	1.72
10	83.47	4.52
100	47.60	7.51

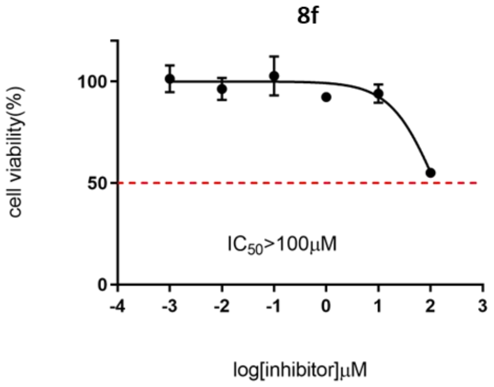

Conc.(μM)	Viability	
	AVE.	±SD
0	100.00	6.63
0.01	96.34	5.39
0.1	102.74	9.63
1	92.36	1.92
10	94.00	4.58
100	55.00	1.00
